# Transcriptome Analysis of Fusarium–Tomato Interaction Based on an Updated Genome Annotation of *Fusarium oxysporum* f. sp. *lycopersici* Identifies Novel Effector Candidates That Suppress or Induce Cell Death in *Nicotiana benthamiana*

**DOI:** 10.3390/jof8070672

**Published:** 2022-06-26

**Authors:** Xizhe Sun, Xiangling Fang, Dongmei Wang, David A. Jones, Lisong Ma

**Affiliations:** 1 State Key Laboratory of North China Crop Improvement and Regulation, Hebei Agricultural University, Baoding 071001, China; xizhesun@gmail.com (X.S.); dongmeiwang63@126.com (D.W.); 2 Division of Plant Science, Research School of Biology, the Australian National University, Canberra 2601, Australia; 3 Hebei Key Laboratory of Plant Physiology and Molecular Pathology, College of Life Science, Hebei Agricultural University, Baoding 071001, China; 4 State Key Laboratory of Grassland Agro-Ecosystems, Key Laboratory of Grassland Livestock Industry Innovation, Ministry of Agriculture and Rural Affairs, College of Pastoral Agriculture Science and Technology, Lanzhou University, Lanzhou 730020, China; xlf@lzu.edu.cn; 5 College of Horticulture, Hebei Agricultural University, Baoding 071001, China

**Keywords:** Fusarium, tomato, novel effector candidates, cell death, *Nicotiana benthamiana*

## Abstract

*Fusarium oxysporum* f. sp. *lycopersici* (*Fol*) causes vascular wilt disease in tomato. Upon colonization of the host, *Fol* secretes many small effector proteins into the xylem sap to facilitate infection. Besides known SIX (secreted in xylem) proteins, the identity of additional effectors that contribute to *Fol* pathogenicity remains largely unexplored. We performed a deep RNA-sequencing analysis of *Fol* race 2-infected tomato, used the sequence data to annotate a published genome assembly generated via PacBio SMRT sequencing of the *Fol* race 2 reference strain Fol4287, and analysed the resulting transcriptome to identify *Fol* effector candidates among the newly annotated genes. We examined the *Fol*-infection expression profiles of all 13 *SIX* genes present in *Fol* race 2 and identified 27 new candidate effector genes that were likewise significantly upregulated upon *Fol* infection. Using Agrobacterium-mediated transformation, we tested the ability of 22 of the new candidate effector genes to suppress or induce cell death in leaves of *Nicotiana benthamiana*. One effector candidate designated *Fol-EC19*, encoding a secreted guanyl-specific ribonuclease, was found to trigger cell death and two effector candidates designated *Fol-EC14* and *Fol-EC20*, encoding a glucanase and a secreted trypsin, respectively, were identified that can suppress *Bax*-mediated cell death. Remarkably, *Fol-EC14* and *Fol-EC20* were also found to suppress *I-2*/*Avr2*- and *I*/*Avr1*-mediated cell death. Using the yeast secretion trap screening system, we showed that these three biologically-active effector candidates each contain a functional signal peptide for protein secretion. Our findings provide a basis for further understanding the virulence functions of *Fol* effectors.

## 1. Introduction

Plants continuously face biotic stresses associated with fungal pathogens. To combat fungal infections, plants have evolved multiple cell-surface and intracellular receptors able to perceive pathogen molecules and activate plant immune responses [1]. Conversely, adapted fungal pathogens must avoid or suppress plant immune responses to establish compatible infections [2]. One of the strategies employed by fungal pathogens is the secretion of effectors that can suppress plant immune responses and manipulate plant cell physiology to facilitate infection and fungal proliferation [2,3]. However, some effectors can be recognized by plant receptors to activate effector-triggered immunity (ETI) [2,4]. ETI often results in a rapid and localized cell death that can impede the proliferation of biotrophic fungal pathogens [5]. In turn, fungal pathogens have evolved strategies to avoid ETI via genetic changes in effector genes (also termed *Avr* or avirulence genes) recognized by corresponding *R* (resistance) genes in the host or to suppress ETI by deploying other effectors. For example, the hemibiotrophic fungal pathogen *Leptosphaeria maculans* has been shown to use AvrLm3 and AvrLm5-9 effectors to suppress race-specific resistance mediated by Rlm4-7, which perceives the intracellularly localized AvrLm4-7 effector [6,7,8]. The wheat powdery mildew effector SvrPm3^a1/f1^ suppresses Pm3-mediated race-specific resistance triggered by the distantly related AvrPm3s [9]. Similarly, the *Fusarium oxysporum* f. sp. *lycopersici* Avr1 effector contributes to pathogenicity by suppressing resistance mediated by the I-2 and I-3 R proteins [10]. Nine wheat rust effectors have been shown to suppress the cell-death response caused by the transient co-expression of different *Avr*/*R* gene combinations in *Nicotiana benthamiana*, implying a possible role in suppressing ETI [11].

The large-scale identification of putative fungal effectors remains challenging due to a lack of sequence conservation among known fungal effectors. However, the availability of fungal genome sequences and RNA-seq techniques have facilitated high throughput and accurate identification of putative effectors, thereby accelerating our understanding of the molecular interactions between plants and pathogens [12]. Comprehensive and enhanced bioinformatics pipelines have been developed to successfully predict effector candidates from plant pathogen genomes. For example, a bioinformatics pipeline was used to identify 725 candidate effectors from the genome and infection transcriptome of the flax rust pathogen *Melampsora lini* [13]. With a similar strategy and additional selection criteria, 78 effector candidates were identified from the necrotrophic plant pathogen *Sclerotinia sclerotiorum* [14]. Similarly, 80 effector candidates were identified via a transcriptomic analysis of the hemibiotrophic fungal pathogen *Leptosphaeria maculans* during the colonization of *Brassica napus* cotyledons [15] and 32 during infection of *Arabidopsis thaliana* by *Plasmodiophora brassicae* [16].

Unlike bacterial effectors, large-scale determinations for the virulence roles of putative fungal effectors remain limited owing to the difficulties in obtaining sufficient or multiple gene knockout mutants [17]. Because host cell death plays an important role in plant–pathogen interaction, the identification of effectors with the ability to suppress or induce cell death has been of long-standing interest. *Agrobacterium*-mediated transient expression in planta has been demonstrated as an effective high-throughput tool to rapidly identify fungal proteins with the ability to suppress host cell death associated with plant immunity or to induce cell death [18]. Cell death triggered by the pro-apoptotic mouse protein Bax in plants represents a similar defense-related cell-death response [19]. The suppression of Bax-induced cell death by co-expression of effectors in *N. benthamiana* with *Bax* has been widely used as an efficient screening tool to identify many putative fungal effectors [11,15,20,21,22,23,24,25]. A growing number of fungal effectors have also been found to induce cell death by transient expression in planta, and they can be initially considered “elicitors” or “toxins” or potential avirulence determinants recognized by plant resistance proteins [26,27]. However, their roles in plant–fungal interactions remain controversial.

*Fusarium oxysporum* f. sp. *lycopersici* (*Fol*) is a soil-borne hemibiotrophic fungal pathogen that causes vascular wilt of tomato and severe yield losses in tomato production [28]. *Fol* enters the tomato root through natural wounds or by direct penetration of epidermal cells. Following the colonization of the root, *Fol* invades the xylem where it produces microconidia and rapidly proliferates to spread throughout the xylem. The accumulation of fungal biomass results in typical wilt disease symptoms [29]. Because the xylem serves as the primary interface between the host and pathogen, 14 small secreted *Fol* proteins (<25 kDa), named SIX (secreted in xylem) proteins, have been identified in the xylem sap of *Fol*-infected tomato plants by proteomic analysis [30,31,32]. Most of the *SIX* genes are located on the *Fol* pathogenicity chromosome 14 [32,33,34]. Knockout mutants of five *SIX* genes, namely *SIX1* (*Avr3*) [30], *SIX2* [35], *SIX3* [36], *SIX5* [37], and *SIX6* [38], are compromised in pathogenicity on susceptible tomato plants, showing that they are required for virulence. Further studies showed that the complete loss of the long arm of chromosome 14, containing *SIX6*/*9*/*11*, and part of the short arm, including *SIX7*/*10*/*12*, did not abolish *Fol* pathogenicity [34,39]. Recently, a conserved *SIX8-PSE1* (*pair with SIX eight 1*) gene pair in *F. oxysporum* isolates infecting Arabidopsis was found to suppress phytoalexin-based immunity [40]. A *PSE1* homolog designated *PSL1* (*PSE1*-*like 1*) has also been found paired with *SIX8* in *Fol* [40]. *PSL1* also encodes a small, secreted protein likely to be secreted into the xylem of *Fol*-infected tomato plants.

Currently, four resistance genes have been introgressed into commercial tomato cultivars to protect against *Fol* infection and they have been named *I* (for Immunity), *I-2*, *I-3,* and *I-7* [41]. Avr2 (Six3) is recognized by the intracellular coiled-coil nucleotide-binding leucine-rich repeat (CC-NB-LRR) receptor I-2 [36,42], whereas Avr1 (Six4) and Avr3 (Six1) are recognized by the cell-surface receptors I (a LRR receptor protein; LRR-RP) and I-3 (an S-receptor-like kinase; SRLK), respectively [10,30,43,44]. The heterologous co-expression of *I-2*/*Avr2* triggers cell death in *N. benthamiana* and tomato [36]. Similarly, heterologous co-expression of *I*/*Avr1* triggers cell death in *N. benthamiana* [44]. The arms race between *Fol* and tomato results in the emergence of new pathogenic races able to overcome the resistance conferred by *I* genes and is driven by the evolutionary adaption of *Fol* effector genes to the host [28,45]. Therefore, the discovery of novel *Fol* effectors is required to completely understand the virulence mechanisms evolved by *Fol* and counter this threat. Newly identified effectors can be utilized as probes to identify new resistance genes or to determine susceptibility loci in vulnerable crops [46,47].

To identify new effectors contributing to *Fol* virulence and to obtain a comprehensive expression profile of known *SIX* genes and new effector candidates during *Fol* infection, we performed a genome-wide transcriptomic analysis to identify genes encoding small, secreted proteins upregulated during infection of tomato at 2, 4, and 6 days post inoculation (dpi). We identified 40 effector candidates and obtained expression profiles for known *SIX* genes and novel effector candidates. We validated the secretion signal peptide of selected effector candidates and characterized the ability of effector candidates to suppress *Bax*-, *Avr2*/*I-2*-, and *Avr1*/*I*-induced cell death in *N. benthamiana*. In addition, we examined their ability to induce cell death in *N. benthamiana*. Our findings provide strong evidence that some of the new effector candidates contribute to *Fol* pathogenicity.

## 2. Materials and Methods

### 2.1. Plant Material and Growth Conditions

The tomato cultivar Moneymaker, which is susceptible to all *Fol* races, was used in this study. Moneymaker seedlings were germinated and grown in a growth chamber at 22 °C, 16 h days (100 μE m^−2^ s^−1^), and 18 °C, 8 h nights. *Nicotiana benthamiana* plants were grown in a growth chamber at 25 °C with a 16 h (100 μE m^−2^ s^−1^) day length.

### 2.2. Fol Strain and Inoculations

*Fol* race 2 isolate Fol007 (carrying *Avr2* and *Avr3*) was used in this study. Ten-day-old Moneymaker seedlings were inoculated with *Fol* spores according to the root dip method [48]. After inoculation, the plants were kept in a growth chamber at 22 °C with a 16 h day length, as described above.

### 2.3. Extraction of RNA for RNA-Seq and qRT-PCR Analysis

For RNA extraction, mock- or *Fol*-inoculated Moneymaker seedlings were grown on vermiculite supplemented with nutrients. The roots of six plants per treatment at 2, 4, and 6 dpi were harvested and frozen in liquid nitrogen. This experiment was repeated three times to generate biological replicates. For RNA extraction from mycelium, Fol007 mycelia were grown in minimal growth medium (3% *w*/*v* sucrose, 1% *w*/*v* KNO_3_, and 0.17% *w*/*v* yeast nitrogen base without amino acids or ammonia) at 25 °C and 175 rpm for five days, and mycelium was collected, quickly dried, and frozen in liquid nitrogen. RNA was extracted as described previously [15]. Briefly, six roots per sample or 1 mg of mycelium were ground in liquid nitrogen. The ground samples were extracted using TRIzol LS reagent (Invitrogen, Carlsbad, CA, USA) and purified using the PureLink™ RNA Mini Kit following the manufacturer’s instructions (Invitrogen). DNA was removed with PureLink DNase Set (Invitrogen) using the on-column approach. RNA was quantified by a Qubit fluorometer (Invitrogen) and checked for quality by an Agilent Bioanalyzer 2100 (Agilent Technologies, Santa Clara, CA, USA) following the manufacturer’s protocol. Samples with integrity numbers above 8.0 were used for RNA-seq. 

For qRT-PCR analysis, cDNA was synthesized using the iScript™ cDNA Synthesis Kit (Bio-Rad, Hercules, CA, USA). qRT-PCR primers were designed using Primer-Blast [49] and checked against the *Fol* genome for specificity (Table S1). qRT-PCR was performed using Applied Biosystems ViiA™ 7 Real-Time PCR System (Applied Biosystems, Waltham, MA, USA) and SYBR Green Master Mix (Applied Biosystems). The *Fol ACTIN* gene was used as a reference. The quantification of relative gene expression levels was performed using the 2^−△△Ct^ method [50].

### 2.4. RNA-Seq-Guided Genome Annotation and Genome Alignment

RNA-seq libraries were prepared and sequenced by Novogene (Beijing, China) using a HiSeq 2500 platform (Illumina, Inc., San Diego, CA, USA). To predict and annotate genes, we aligned RNA-seq reads (150 bp paired end) to the recent assembly of the Fol4287 genome generated by SMRT (single molecule real time) PacBio sequencing (GCA_001703175.2 [34]) using HISAT2.2 with the parameter exon [51]. Scallop v0.10.4 [52] was used to assemble the paired reads into transcripts. All assembled transcripts were used for gene prediction by CodingQuarry v2.0 (-d parameter) [53] and aligned reads were used for gene prediction by BRAKER v2.1.6 with nondefault parameters: fungus–species = “fusarium_oxysporum” [54]. These two gene prediction outputs were combined using funannotate pipeline v 1.8.7 (https://github.com/nextgenusfs/funannotate/ accessed on 10 July 2021), which passes the combined gene predictions onto EVidenceModeler v.1.1.1 to generate a consensus annotation of protein-coding genes (https://zenodo.org/record/4054262#.X8MXPM0zaHs accessed on July 2021). The completeness of gene prediction was examined using BUSCO v.5.2.1 (benchmarking universal single-copy ortholog) with the “fungi_odb10” library [55].

MCScanX toolkit was used to distinguish between collinear and lineage-specific (LS) regions in the Fol4287 genome assembly generated via SMRT PacBio sequencing (GCA_001703175.2) relative to the *Fusarium verticillioides* (*Fv*) genome [56]. Chromosome regions with at least ten orthologous gene pairs shared between the *Fol* genome and *Fv* genomes were identified as collinear. Orthologous genes were identified via BLASTp with an E-value cut-off of 1 × 10^−10^ [57]. 

The Fol4287 genome assembly generated via SMRT PacBio sequencing (GCA_001703175.2) was aligned with the previous Fol4287 genome assembly generated via Sanger sequencing (GCA_000149955.2 [33]) using MUMmer 3.23 with maxmatch [58]. LS regions, the locations of candidate effector genes, and the relationship between the two genome assemblies were visualized via Circos plots constructed using Advanced Circos in TBtools [59].

### 2.5. Secretome Prediction

The pipeline used in this study for the prediction of the *Fol* secretome was modified from previous studies [13,15,60]. Briefly, SignalP 5.0 [61] was used to predict signal peptides with a cut-off of 0.8. Mature protein sequences (with signal peptides removed) were used as inputs for TMHMM v2.0c [62] to identify proteins with transmembrane domains, which were then removed. All protein sequences remaining after this step were checked by TargetP 1.1 [63] to remove proteins targeted to mitochondria. ScanProsite [64] was then used to remove proteins targeted to the endoplasmic reticulum. Finally, PredGPI [65] was used to remove proteins with glycosylphosphatidylinositol anchors with a false positive rate ≤ 0.01.

### 2.6. Gene Expression Analysis

The expression of newly assembled transcripts was quantified via Salmon 1.5.1 [66], which was set with the argument KeepDuplicates in the indexing step and validateMappings and numBootstraps 100 in the quantification step. Differential gene expression analysis was performed using the Bioconductor package DESeq2 v. 1.6.3 [67] by comparing the gene expression at different time points in Fol007-inoculated samples with gene expression in the mycelium sample. Clustered heatmaps showing the expression profiles of candidate effector genes were generated using TBtools [59].

### 2.7. Plant Transformation Vector Construction

To generate constructs for transient expression in *N. benthamiana*, 22 effector candidate genes were amplified using primers listed in Supplementary Table S1. The resulting PCR products were cloned into the in-house ligation-independent cloning (LIC) vector pSL, which was derived from pGreenII [68] and carries a CaMV 35S promoter and tobacco PR1a signal peptide sequence, using the protocol described previously [69]. The resulting pSL: *effector–candidate–gene* constructs were used for Agrobacterium transformation, as described previously [70].

### 2.8. Agrobacterium-Mediated Transient Assays in N. benthamiana

The Agrobacterium-mediated transient transformation of *N. benthamiana* was performed according to methods described previously [70]. Briefly, Agrobacterium cultures were grown to an absorbance of 0.8 at 600 nm in LB-mannitol medium supplemented with 20 µM acetosyringone and 10 mM MES pH 5.6. Cells were pelleted by centrifugation at 2800× *g* for 20 min and then resuspended in infiltration medium (10 mM MES pH 5.6, 2% *w*/*v* sucrose, 200 µM acetosyringone) to an absorbance at 600 nm of 1. Infiltrations were conducted on leaves of 4–5-week old *N. benthamiana* plants. The resulting responses were photographed 5 days after infiltration.

### 2.9. Yeast Secretion Trap Assay

The predicted coding sequences of the signal peptides of selected effector candidates were amplified from cDNA using the primers listed in Table S1 and cloned into the yeast secretion trap vector pSUC2 using *Eco*RI and *Xho*I restriction sites. The signal peptide coding sequence of *N. benthamiana* pathogenesis-related protein 1a (PR1a) was also cloned into pSUC2 for use as a positive control. The resulting constructs were transformed into the yeast strain YTK12 to examine secretion following the method described previously [71,72]. Briefly, yeast transformants able to grow on selective CMD-W media (6.7 g yeast nitrogen base without amino acids, 20 g sucrose, 1 g glucose, and 0.74 g minus tryptophan dropout supplement in 1000 mL distilled water with 15 g agar) [72] were transferred to fresh CMD-W and YPRAA plates (1% *w*/*v* yeast extract, 2% *w*/*v* peptone, 2% *w*/*v* raffinose, and 2 µg/mL antimycin A). The secretory function of a putative signal peptide is determined by the growth of colonies on YPRAA plates after 3 days incubation at 30 °C and the reduction of colorless 2,3,5-triphenyltetrazolium chloride (TTC) to red-colored insoluble triphenylformazan, as described by Yin et al. [72].

## 3. Results

### 3.1. Identifying Candidate Effector Genes Expressed during Fol Infection

To identify novel *Fol* effector candidates expressed during *Fol* infection, we performed RNA-seq analysis using RNA samples prepared from Moneymaker tomato plants at 2, 4 and 6 dpi with *Fol* race 2 and from mycelium grown in vitro. The Fol4287 reference genome was recently re-sequenced using PacBio SMRT sequencing to improve the quality of the genome sequence and assembly [34]. We aligned the previous Fol4287 genome assembly (GCA_000149955.2 [33]) based on Sanger sequencing, here designated as the *Fol* 2010 genome, with the newly assembled genome (GCA_001703175.2), here designated as the *Fol* 2020 genome, and found the assembly of LS chromosomes differed markedly between the two assemblies (Figures S1 and S2). Based on this observation, we decided to map RNA-seq reads to the *Fol* 2020 genome. For RNA samples prepared from *Fol* infected tomato, 80–97 million raw reads were produced and after filtering out small reads, 0.35–1.31 million paired-end reads were mapped to the *Fol* 2020 genome, accounting for 0.39%–1.35% of the reads (Table S2). For RNA prepared from *Fol* growing in vitro, a total of 30.2 million raw reads were produced and 29.2 million paired-end reads were mapped to the *Fol* 2020 genome. Next, we annotated the *Fol* 2020 genome using our RNA-seq data, resulting in 26,826 gene models (Table S3). Genome annotation quality was assessed using the BUSCO tool, which produced 751 complete, 3 fragmented, and 4 missing BUSCOs from a total of 758 BUSCO groups based on the fungi_obd10 database, resulting in a 99.5% BUSCO completeness score (Table S4). The annotated protein-coding sequences were assigned FOXGR gene IDs and used to search against the old *Fol* 2010 proteome. Identical sequences with their old FOXG gene IDs were also listed (Table S3). In total, 26,826 predicted proteins were used as the basis for the prediction of the *Fol* secretome. 

**Figure 1:**
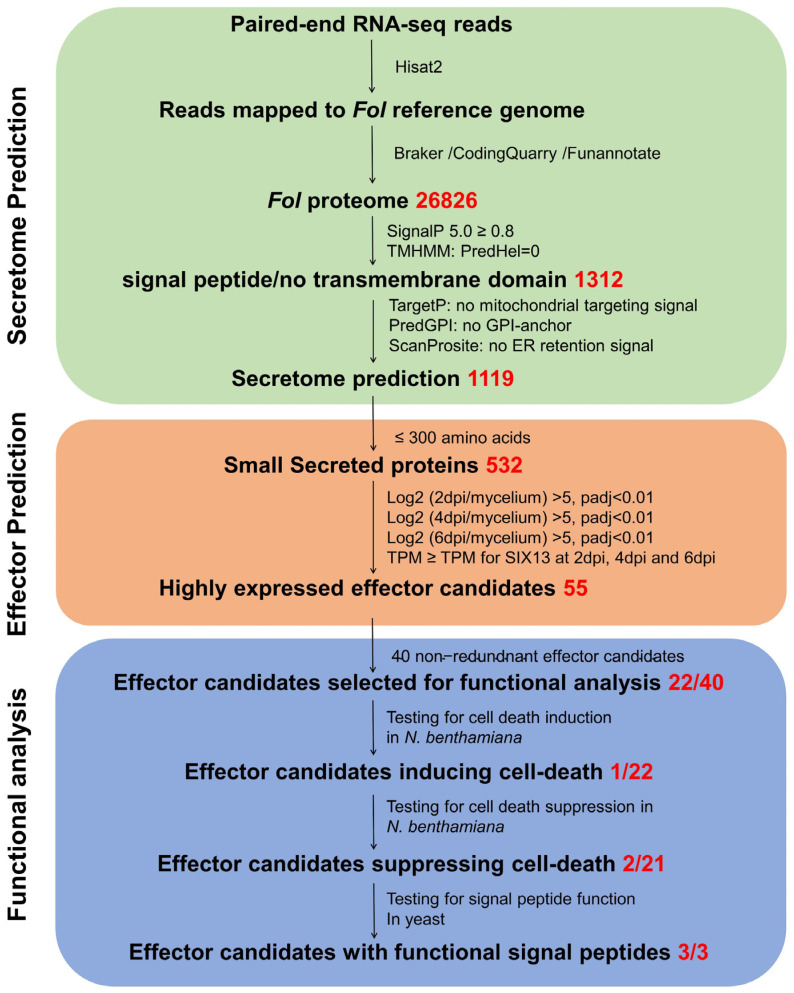
**Pipeline for the bioinformatic prediction and functional analysis of *Fol* effector candidates.** Three main steps are included in this pipeline: Prediction of the *Fol* secretome based on transcripts aligned to the PacBio-sequenced Fol4287 reference genome, identification of *Fol* genes encoding small, secreted proteins that were differentially expressed during *Fol* infection and functional analysis of novel effector candidates. Based on the expression profile of the *SIX13* gene, which had the lowest transcript per million (TPM) value among the 13 *SIX* genes present in *Fol* race 2, 55 genes were selected as effector candidates. Functional analysis was carried out on 22 of the 40 non-redundant effector candidates, excluding the 13 *SIX* genes present in *Fol* race 2.

Based on the effector prediction pipelines employed previously for other plant pathogens and the characteristics of known effectors of plant pathogenic fungi, e.g., less than 300 amino acids in length [13,15,60,73], a bioinformatics pipeline was adopted to identify the *Fol* effector candidates expressed during infection (Figure 1) This pipeline involved three main processes: secretome prediction, effector prediction, and functional analysis (Figure 1). As shown in Figure 1, 1312 proteins were identified with a signal peptide and no transmembrane domain, consistent with secretion via the classical secretory pathway. After removing proteins predicted to target the ER or mitochondria, and GPI anchored proteins, a total of 1119 proteins were included in the predicted *Fol* 2020 secretome, accounting for 4.17% of the predicted *Fol* 2020 proteome. Of these 1119 proteins, 532 were ≤300 amino acids and of these 77 proteins were encoded by genes that were significantly up-regulated at 2, 4, and 6 dpi during *Fol* infection in comparison to their expression in mycelium, including all of the *SIX* genes present in *Fol* race 2. As *SIX13* exhibited the lowest transcript per million (TPM) values among all *SIX* genes at 2, 4, and 6 dpi, we selected 55 secreted-protein-coding genes that had TPM values equal to or greater than that of *SIX13* at each time point as a set of *Fol* effector candidates (Table 1). These included two identical copies of *SIX13* and eight identical copies each of *SIX8* and *PSL1*, leaving a non-redundant total of 40 candidate effectors. Next, the sequence annotations of these candidate effector genes were validated by the alignment of reads to the reference genome.

**Figure 2:**
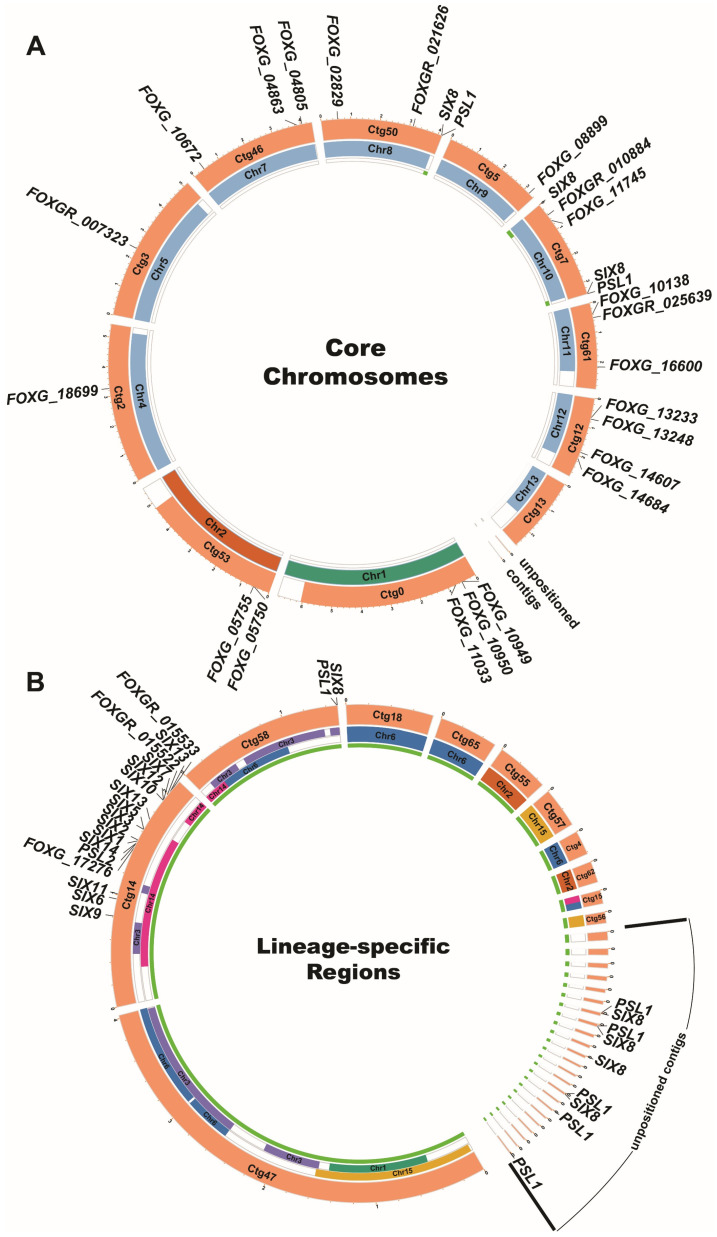
**Distribution of the 55 *Fol* effector candidate genes on the physical map of the Fol4287 genome.** Circos plots showing the distributions of *Fol* effector candidate genes on the core chromosomes (**A**) and the lineage-specific (LS) regions (**B**). In both Circos plots, the orange segments in the outer circle represent the contigs assembled following the PacBio sequencing of the Fol4287 genome. The colored segments in the middle circle represent the corresponding chromosomes assembled following Sanger sequencing of the Fol4287 genome. The various colors have been used to highlight the extensive rearrangement of the LS chromosomes and LS regions in the new PacBio assembly compared to the old assembly. The thin green segments on the inner circle represent the LS regions in the Fol4287 genome compared to the *Fusarium verticillioides* genome.

In addition, we generated Circos plots to visualise the chromosomal distribution of *Fol* effector candidates. As shown in Figure 2A, 22 effector candidates are located on *Fv*-collinear regions of the 11 core chromosomes in the *Fol* genome. However, effector candidate *FOXGR_025639* had no ortholog in *Fv* despite having orthologs in other *Fusarium* species, including *F. graminearum*. Four of these 22 effector candidates, *FOXGR_007323*, *FOXGR_010884*, *FOXGR_021626*, and *FOXGR_025639*, were either not predicted or predicted incorrectly in the annotation of the *Fol* 2010 genome assembly. As shown in Figure 2B, 33 effector candidates were located in LS regions compared to the *Fv* genome, most on chromosome 14. Two *SIX8*/*PSL1* gene pairs and one lone copy of *SIX8* were located in LS regions near the ends of core chromosomes 8 and 10 in the *Fol* 2020 assembly; one *SIX8*/*PSL1* gene pair on contig 58; a large chromosome-sized contig similar to LS chromosome 3 in the *Fol* 2010 genome assembly; and three *SIX8*/*PSL1* gene pairs, one lone copy of *SIX8* and two lone copies of *PSL1* on small unpositioned contigs (Figure 2). Four new effector candidates were identified on chromosome 14, including *FOXGR_015533*, which was completely absent from the *Fol* 2010 genome sequence; *FOXGR_015322* (a homologue of *PSE1* here designated *PSL2*), which was not predicted in the annotation of the *Fol* 2010 genome assembly; and *FOXGR_015522*, which was predicted incorrectly. All 13 of the *SIX* genes present in *Fol* race 2 and *PSL1* were identified in the *Fol* 2020 assembly, whereas *SIX7*, *SIX8*, *SIX10*, *SIX11*, *SIX12*, and *PSL1* were either not predicted or predicted incorrectly in the annotation of the *Fol* 2010 genome assembly. 

### 3.2. Expression Profiles of Fol Effector Candidates during Infection

**Figure 3:**
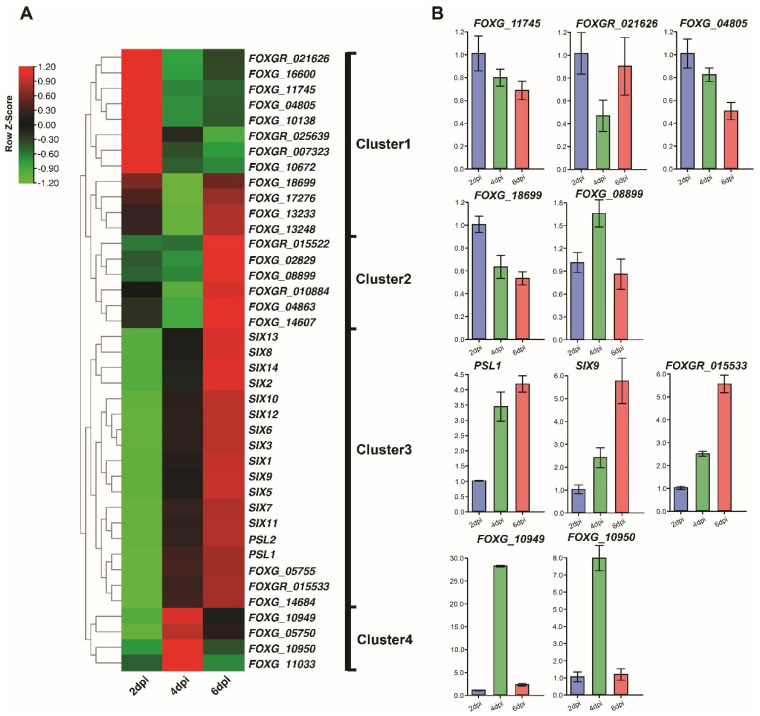
**Expression profiles of effector candidate genes and the validation of the expression of selected effector candidate genes during *Fol* infection by RT-qPCR.** (**A**) Clustered heatmaps of the 40 differentially-expressed *Fol* effector candidate genes at 2, 4, and 6 dpi based on RNA-seq data. The row color scale reflects the values of log2 (mean TPM at each time point), in which red represents higher expression and green represents lower expression. Genes are grouped into four clusters with distinct expression profiles over the three-time points. (**B**) Quantitative RT-PCR analysis showing the expression of effector candidate genes selected from each cluster in infected tomato roots at 2, 4 and 6 dpi. *Fol* actin was used as an internal reference gene and expression levels were normalized to actin. Values are means of three independent biological samples and error bars represent standard deviations.

To explore the expression profiles of *Fol* effector candidates upon infection, we performed a hierarchical clustering heatmap analysis of the 40 non-redundant effector candidates using their TPM values. Figure 3A shows that they grouped into four clusters correlated with their expression at three different *Fol* infection time points. Cluster 1 contains genes that were highly up-regulated at 2 dpi but less so at 4 and 6 dpi. Cluster 2 represents genes that were highly up-regulated at 2 and 6 dpi but less so at 4 dpi. All 13 *SIX* genes were included in Cluster 3, which contains genes up-regulated progressively from 2 to 6 dpi. Genes in Cluster 4 were highly up-regulated at 4 dpi but less so at 2 and 6 dpi. Our results indicate that all identified *Fol* effector candidate genes exhibit high expression upon *Fol* infection and that candidates in Clusters 2 and 3, which were most up-regulated at 6 dpi, might play important roles during *Fol* infection, given that all *SIX* genes that contribute to *Fol* pathogenicity were highly up-regulated at 6 dpi. 

To validate the quantitative gene expression detected in the RNA-seq analysis (Figure 3B), a total of ten differentially expressed effector candidates, including two or three genes from each cluster, were selected for validation. Their relative expression levels at 2, 4, and 6 dpi were quantified by qRT-PCR. The qRT-PCR expression profiles of all ten genes confirmed their upregulation during infection and seven of the ten showed similar patterns of expression to those evident from the RNA-seq data (Figure 3B).

### 3.3. Identification of Cell Death-Inducing Effector Candidates

**Figure 4:**
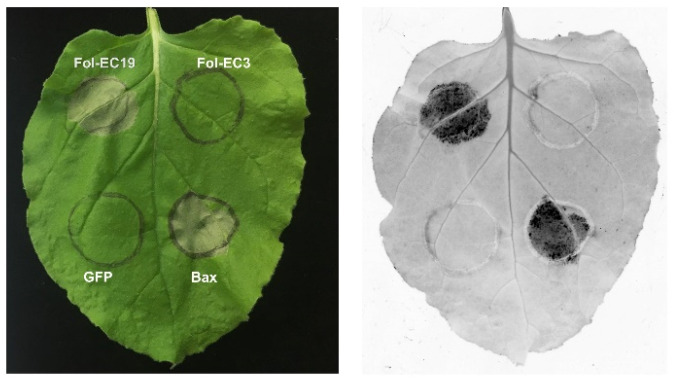
***Fol-EC19* induces cell death in the leaves of *N. benthamiana*.***N. benthamiana* leaves were agro-infiltrated with a construct containing the *Fol-EC19* effector candidate gene. *GFP* and *Fol-EC3* served as negative controls and *Bax* served as a positive control. Representative leaves were photographed 3 days after infiltration and the experiment was repeated at least three times with consistent results.

Host cell death is a hallmark of defense against a biotrophic fungal pathogen but could contribute to pathogenicity for a hemi-biotrophic pathogen like *Fol*. To identify effector candidates inducing cell death, 22 effector candidate genes were selected for cloning and transient expression via agroinfiltration of *N. benthamiana* leaves (Table 1). *GFP* was used as a negative control and *Bax* was used as a positive control in these experiments. We found that one of the 22 effector candidates, *Fol-EC19*, triggered cell death at 3 dpi (Figure 4). In addition, we used RT-PCR to show that all 22 candidate genes were expressed in *N. benthamiana*. (Figure S3). 

### 3.4. Identification of Effector Candidates That Suppress Bax-Induced Cell Death Using a Transient Expression Assay

**Figure 5:**
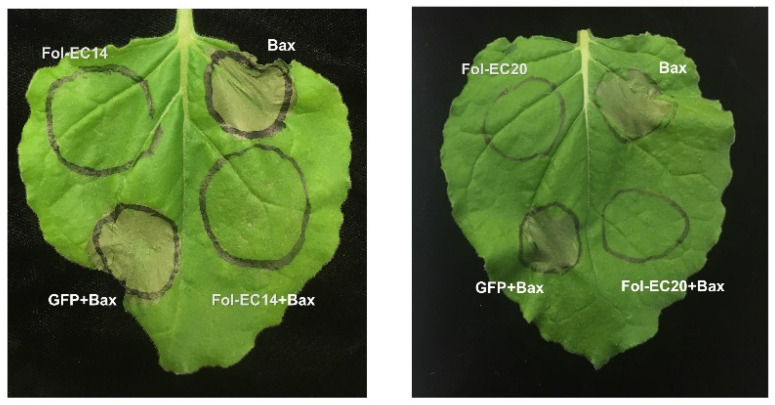
***Fol-EC14* and *Fol-EC20* can suppress *Bax*-induced cell death.***A. tumefaciens* carrying either *Fol-EC14* or *Fol-EC20* effector candidate gene was infiltrated into *N. benthamiana* leaves, followed 24 h later by infiltration with *A. tumefaciens* carrying the *Bax* gene. *GFP* served as a negative control. Representative leaves were photographed 3 days after infiltration and the experiment was repeated at least three times with consistent results.

Many fungal effectors suppress host cell-death to facilitate pathogen infection [74]. To identify effector candidates that can suppress *Bax*-induced cell death, we co-agroinfiltrated *N. benthamiana* leaves with *Bax* and each of the remaining 21 effector genes used for the cell-death experiments described above. *GFP* was used as a negative control. *Bax* triggers rapid cell death in *N. benthamiana* leaves at 2 dpi (Figure 5). *Fol-EC14* and *Fol-EC20* were found to completely suppress *Bax*-induced cell death whereas leaf regions co-expressing *GFP* and *Bax* developed pronounced cell death (Figure 5). 

### 3.5. Suppression of I/Avr1- and I-2/Avr2-Induced Cell Death

**Figure 6:**
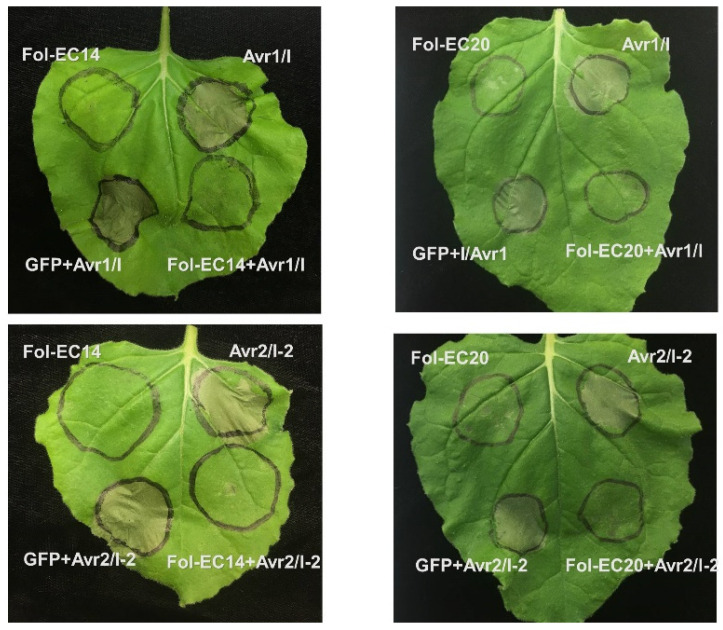
***Fol-EC14* and *Fol-EC20* can suppress cell death triggered by Fusarium-wilt resistance genes.***A. tumefaciens* carrying either *Fol-EC14* or *Fol-EC20* effector candidate gene was infiltrated into *N. benthamiana* leaves followed 24 h by co-infiltration with *A. tumefaciens* carrying the *Avr1* and *I* or *Avr2* and *I-2* genes. *GFP* served as a negative control. Representative leaves were photographed 3 days after infiltration and the experiment was repeated at least three times with consistent results.

To investigate whether *Fol-EC14* and *Fol-EC20* function as suppressors of cell death triggered by tomato proteins conferring *Fol*-resistance, we transiently co-expressed *Fol-EC14* and *Fol-EC20* with *I*/*Avr1* or *I-2*/*Avr2* in *N. benthamiana* leaves, using *GFP* as a negative control. No cell death was observed in leaf regions co-expressing *Fol-EC14* or *Fol-EC20* with *I*/*Avr1* or *I-2*/*Avr2* (Figure 6). In contrast, co-expression of *I*/*Avr1* or *I-2*/*Avr2* with *GFP* triggered rapid cell death at 3 dpi. These findings indicated that Fol-EC14 and Fol-EC20 inhibited cell death triggered by both a CC-NB-LRR resistance protein (I-2 in this instance) and an LRR-RP resistance protein (I in this instance).

To further explore whether *Fol-EC14* and *Fol-EC20* are able to suppress cell death triggered by other plant *R* genes, we transiently co-expressed them with *L6TIR* in *N. benthamiana* leaves. The TIR (Toll Interleukin-1 Receptor) domain of the flax *L6* TIR-NB-LRR resistance protein is able to homodimerize in the absence of its NB and LRR domains to trigger plant cell death [75]. *L6TIR*-induced cell death was not inhibited by either *Fol-EC14* or *Fol-EC20* (Figure S4). In addition, we examined their ability to suppress *Fol-EC19*-induced cell death. Neither *Fol-EC14* nor *Fol-EC20* was able to suppress cell death induced by *Fol-EC19* (Figure S4).

### 3.6. Functional Characterization of the Signal Peptides of the Fol-EC14, Fol-EC19, and Fol-EC20 Effectors

**Figure 7:**
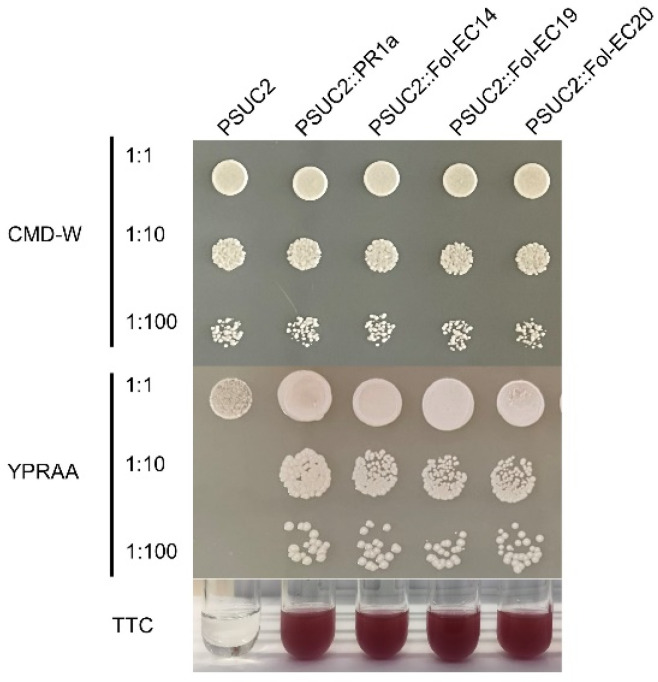
**Yeast secretion trap assay of the predicted signal peptides of cell-death inducing and cell-death suppressing effector candidates.** The predicted signal peptide coding sequences of *Fol-EC14*, *Fol-EC19*, and *Fol-EC20* were cloned into the yeast secretion trap vector pSUC2. The tobacco PR1a signal peptide was used as a positive control and empty vector was used as a negative control. CMD-W (-Trp) was used to select the transformed yeast YTK12 carrying the pSUC2 vector. Yeast growing on the YPRAA medium indicate secretion of invertase via a functional signal peptide. Conversion of the dye 2, 3, 5-triphenyltetrazolium chloride (TTC) to the insoluble red colored triphenylformazan is also indicative of invertase secretion.

To examine the function of the predicted signal peptide sequences of Fol-EC14, Fol-EC19, and Fol-EC20, a yeast signal sequence trap system was employed. The predicted signal peptide coding sequences of *Fol-EC14*, *Fol-EC19*, and *Fol-EC20* were amplified and cloned in frame with a truncated *SUC2* gene, which encodes yeast invertase, lacking its signal peptide. The resulting constructs and positive control pSUC2::SP^PR1a^ (carrying the *N. benthamiana* PR1a signal-peptide coding sequence) were transformed into yeast strain YKT12, which cannot grow on medium without a simple sugar due to a deficiency of the yeast invertase gene. Consistent with the positive control, YTK12 strains carrying the predicted signal-peptide coding sequences of *Fol-EC14*, *Fol-EC19*, and *Fol-EC20* fused to *SUC2* were able to grow on YPRAA plates (Figure 7). As expected, they were also able to catalyze the conversion of colorless TTC to red-colored TFP (Figure 7). Conversely, the negative control (YTK12 with empty vector) was not able to grow on a YPRAA plate and did not induce a TTC to TFP color change (Figure 7). These results demonstrated that the predicted signal peptides of Fol-EC14, Fol-EC19, and Fol-EC20 are functional.

## 4. Discussion

The effectors secreted by plant pathogenic fungi determine the outcome of infection by either suppressing plant immune response or interfering with plant physiological processes to facilitate pathogen infection [2,76,77]. The study presented here identified a repertoire of newly assembled *Fol* transcripts and 40 non-redundant effector candidates whose expression is highly induced during tomato infection, including all 13 *SIX* genes present in *Fol* race 2. Functional analysis of 22 of the 27 novel effector candidates revealed that one effector candidate induced cell death and two effector candidates suppressed R-protein-mediated cell death. We further demonstrated that these three effector candidates have a functional signal peptide. Together, these findings enrich the genome annotation of *Fol*, deliver genome-wide expression profiles of genes encoding small, secreted proteins, including the *SIX* genes, and provide a basis for further exploring *Fol* pathogenic mechanisms.

Previously, the annotation of the published Fol4287 reference genome was based on gene prediction without transcriptome analysis [33]. Thus, inevitable inaccuracies occurred, and some still remain. For example, several *SIX* genes, including *SIX7*, *SIX8*, *SIX11*, *SIX12*, and *SIX14* were not annotated in the reference genome but were subsequently submitted as separate NCBI accessions [32]. We have been able to assemble 26,826 transcripts from transcriptome sequencing data mapped to a genome assembly based on PacBio SMRT genome sequencing (Figure 1), including all of the previously unannotated *SIX* genes. In addition, eight effector candidate genes were identified that were absent from the *Fol* 2010 reference genome annotation (Table 1). Of the non-redundant total of 40 candidate effector genes, 18, including all 13 *SIX* genes present in *Fol* race 2, are located on LS regions and the remaining 22 on core chromosomes (Table 1 and Figure 2). Importantly, three of the newly annotated genes, *PSL2*, *FOXGR_015522* and *FOXGR_015533,* and one previously annotated gene, *FOXG_17276*, are located on the LS pathogenicity chromosome 14, providing a strong indication that they might be involved in *Fol* infection. Multiple copies of *SIX8* and the newly annotated *PSL1* gene are located in other LS regions, mostly as divergently oriented pairs (Table 1 and Figure 2). None of the 18 candidate LS effectors had homologs with a known function, although FOXG_17276 has a LysM domain, suggesting a role in carbohydrate binding. In contrast, eight of the 22 candidate core region effectors had homology to proteins of known function, including two hydrophobins, a glucanase, a phospholipase, a ribonuclease, and three proteases (Table 1). Cell wall-degrading, membrane-attacking, and proteolytic enzymes are well-known parts of a plant pathogen’s arsenal, and it is not surprising that degradative enzymes falling below the 300 amino acid threshold should be captured by this analysis as potential effectors. However, roles for a glucanase and trypsin in suppression of defence-related cell death and a ribonuclease in the induction of cell death were unexpected.

*FOXGR_021626*, here designated *Fol-EC14*, encodes a glucanase containing a GH131 glycosyl hydrolase domain, which was first described in the ascomycete *Podospora anserina* as PaGluc131A [78] and in the basidiomycete *Coprinopsis cinerea* as CcGH131A [79]. PaGluc131A has been reported to have β-1,3- and β-1,6-exoglucanase and β-1,4 endoglucanase activities [78], and a later study also reported β-1,3-endoglucanase activity, but could not confirm β-1,6-exoglucanase activity [80]. Despite comprising almost entirely a GH131 domain, Fol-EC14 only has 23% and 24% amino acid sequence identity with the N-terminal glycosyl hydrolase domains of PaGluc131A and CcGH131A, respectively. GH131 proteins are widespread among the Ascomycota and Basidiomycota and an extensive phylogenetic analysis identified two clades, each containing ascomycete and basidiomycete sequences suggesting divergence of the two GH131 clades prior to the divergence of the Ascomycota from the Basidiomycota [80]. Fol-EC14 falls in one clade and PaGluc131A and CcGH131A fall in the other. Interestingly, *Fusarium* is completely absent from the PaGluc131A/CcGH131A clade, although other members of the Sordariomycetes are represented in both (Figures S5 and S6).

Two members of the Fol-EC14 clade, *Colletotrichum higginsianum* ChGluc131A and ChGluc131B (Figures S7 and S8), have also been shown to have β-1,3-exoglucanase and β-1,3- and β-1,4 endoglucanase activities [80]. Fol-EC14 and ChGluc131B and most of their orthologs lack three of the four residues thought to be important for PaGluc131A and CcGH131A catalytic activity [79], whereas ChGluc131A and its orthologs lack only one of these residues (Figures S6–S8). They also differ from members of the PaGluc131A/CcGH131A clade by the presence of two conserved cysteine residues flanking the GH131 domain, which could potentially form a stabilizing disulfide bond (Figures S6–S8). Fol-EC14 has only 43% sequence identity with ChGluc131A and 48% with ChGluc131B, which in turn have only 43% sequence identity with one another. Consistent with these differences, Fol-EC14, ChGluc131A, and ChGluc131B are each member of distinct phylogenetic subgroups within the Fol-EC14 clade (Figures S6–S8), with ChGluc131B and its orthologs limited to the genus *Colletotrichum* (Figure S8), and *Fusarium* sequences present only in the Fol-EC14 subgroup (Figure S6). Like Fol-EC14, ChGluc131B lacks three of the four residues thought to be important for PaGluc131A and CcGH131A catalytic activity but is nevertheless catalytically active. This suggests a different mechanism of catalysis to that proposed for PaGluc131A and CcGH131A, but given the absence of information about residues important for ChGluc131B catalysis and the low sequence identity between Fol-EC14 and ChGluc131B, we cannot infer whether Fol-EC14 is catalytically active or not. If it was functional, possible roles for Fol-EC14 could include degradation of cellulose (β-1,4 glucan) in the plant cell wall, callose (β-1,3 glucan) degradation, conversion of elicitor-active glucans to shorter elicitor-inactive oligomers, and exoglucanase-mediated release of glucose as a nutrient for the fungus. 

*FOXG_13248*, here designated *Fol-EC20,* encodes a secreted trypsin that differs by a single conservative amino-acid substitution from a structurally and enzymatically well-characterized *F. oxysporum* trypsin [81,82]. Trypsins are widespread among plant pathogens, as are trypsin inhibitors among plant hosts. In the xylem-colonizing Gram-positive bacterium *Clavibacter michiganensis* subsp. *michiganensis* (*Cmm*), two genes, *pat-1* and *chpC*, encode secreted trypsin-family proteins that are required for the colonization and wilting of tomato and the suppression of the plant immune response [83,84]. Interestingly, *Cmm* also requires a β-1,4 endoglucanase encoded by the *celA* gene for pathogenicity and wilting of tomato [85]. Potentially, the Fol-EC14 exo/endoglucanase and Fol-EC20 trypsin might play similar roles in *Fol* pathogenicity.

There are three possible explanations for the suppression of Bax-induced and R-protein-induced cell death by Fol-EC14 and Fol-EC20, for which there may be some precedents. One explanation might be that the enzymatic actions of Fol-EC14 or Fol-EC20 suppress plant cell death. Sanchez et al. (1992) reported that water-soluble glucans from *Phytophthora infestans* can suppress fungal-elicitor-induced plant cell death [86], and Ali et al. (2015) reported that a potato cyst nematode expansin could suppress NPP (Nep1-like protein) and CNL-induced cell death [87]. Expansins are plant cell wall-loosening proteins. Given these two observations, it is plausible that either a soluble glucan product of Fol-EC14 catalysis or the effect of Fol-EC14 catalysis on the plant cell wall could have a suppressive effect on plant cell death. Similarly, Carlile et al. (2000) reported that the *Stagonospora nodorum* trypsin SNP1 released hydroxyproline from wheat cell walls and, like β-1,4 endoglucanases, contributes to the modification of the plant cell wall [88]. Hao et al. (2019) reported that a specific *F. graminearum* arabinanase, albeit a different glycosyl hydrolase to Fol-EC14, could suppress Bax-induced cell death [89]. Given that the arabinanase could also suppress flg22- and chitin-induced ROS (reactive oxygen species) production, they inferred that the suppression of Bax-induced cell death was likely related to the suppression of ROS production. A later study by the same group showed that a putative endoglucanase from *F. graminearum* could suppress chitin-induced but not flg22-induced ROS production [90]. No explanation for the mechanisms involved in the suppression of ROS production was provided by either study and, although the products of arabinanase or endoglucanase catalysis could be involved, a direct effect of the arabinanase or endoglucanse protein could not be excluded. A key question that therefore remains to be answered is whether the catalytic activities of Fol-EC14 and Fol-EC20 are required for their suppressive effects.

A second explanation might be that the Fol-EC14 or Fol-EC20 proteins have a direct effect on plant cell death unrelated to enzymatic function. Rose et al. identified non-catalytic members of the trypsin family in *Phytophthora* as inhibitors of plant β-1,3 endoglucanases able to reduce the production of elicitor-active oligoglucans from the *Phytophthora* cell wall [91]. Potentially, this inhibition could promote the production of water-soluble glucans able to suppress fungal-elicitor-induced plant cell death as described above. A third explanation is that the suppression of cell death by Fol-EC14 and Fol-EC20 is an artifact. Bozkurt et al. suggest that the heterologous expression of some proteins can elicit an unfolded protein response in the ER that can suppress Bax-induced cell death [92]. Given that Fol-EC14 and Fol-EC20 have been targeted to the plant ER as part of the secretory pathway, it is possible that they do not fold properly and thereby elicit an unfolded protein response in the ER. However, the fact that they were not able to suppress either L6TIR- or Fol-Ec19-induced cell death suggests this is not the explanation (Figure S4).

*FOXG_13233*, here designated *Fol-EC19*, encodes a secreted guanyl-specific ribonuclease that induces rather than suppresses plant cell death in *N. benthamiana* (Figure 4). Homologs of Fol-EC19 are widely distributed among the fungi including plant pathogens in the genera *Alternaria*, *Bipolaris*, *Colletotrichum*, *Fusarium*, *Magnaporthe*, *Pyrenophora*, *Ustilago*, and *Verticillium*. Our finding is consistent with a recent study showing that *F. graminearum* secretes Fg12, a ribonuclease with 85% identity and 93% similarity to Fol_EC19, which contributes to virulence on soybean as demonstrated by gene knockout experiments and induces cell death in *N. benthamiana*, *N. tabacum*, and tomato, as shown by agroinfiltration experiments [93]. Agroinfiltration of a mutant *Fg12* gene was used to show that induction of cell death in *N. benthamiana* was dependent on protein secretion and ribonuclease activity. Recombinant *F. graminearum* Fg12 protein was found to induce ion leakage and PR-gene expression in *N. benthamiana* and resistance to *F. graminearum* and *Phytophthra sojae* in soybean hypocotyls, again dependent on ribonuclease activity. Whether the cell death induced by Fol-EC19 is dependent on ribonuclease activity and whether Fol-EC19 is capable of inducing plant cell death or plant immune responses in tomato remain unknown but seems likely. These findings suggest that Fol-EC19 might, like Fg12, also function as a virulence factor in the context of pathogen infection but trigger a plant immune response when tested in isolation. Due to limitations of agroinfiltration in tomato, most of the experiments in the present study were conducted using the solanaceous model plant *N. benthamiana*, which has been used extensively in effector biology research and proven useful for translating such research into host species. Further experiments involving purified recombinant candidate-effector proteins or knockouts of effector-candidate genes in *Fol* will provide additional support for the virulence role of these three effector candidates during tomato infection.

## 5. Conclusions

The molecular interaction between tomato and the xylem-colonizing fungus *Fol* has been extensively studied. However, the virulence mechanisms employed by *Fol* effectors remain largely unknown. The study presented here expands the repertoire of *Fol* effector candidates, showing highly-induced expression during tomato infection. Agroinfiltration assays in *N. benthamiana* revealed that one effector candidate, encoding a secreted guanyl-specific ribonuclease, induced cell death and two effector candidates, encoding a glucanase and a secreted trypsin, respectively, suppressed R-protein-mediated cell death. We confirmed that these three biologically active effector candidates have a functional signal peptide that would direct their secretion into the xylem sap of infected tomato plants. These findings suggest that *Fol* utilizes diverse effector proteins to facilitate infection, including enzymatic effectors encoded by the core *Fusarium* genome. Taken together with previous knowledge about the role of lineage-specific effectors, these findings add value to our understanding of *Fol* virulence mechanisms.

## Figures and Tables

**Table 1:** Characteristics of 55 effector candidates with TPM values greater than or equal to that of *SIX13* during *Fol* infection.

Name	Lineage-Specific Region	Chromosome	2020 Contig	Start	End	Strand	Introns	Protein Length	Cys Residues	Protein Domain/Homology
SIX9	YES	14	14	809,946	810,290	-	0	114	6	
SIX6	YES	14	14	962,344	963,070	-	1	225	9	
SIX11	YES	14	14	1,007,574	1,007,906	-	0	110	8	
FOXG_17276 *	YES	14	14	1,450,230	1,450,661	-	0	143	6	LysM domain
PSL2 *	YES	14	14	1,486,868	1,487,339	-	3	106	9	PSE1 homologue
SIX14	YES	14	14	1,489,136	1,489,452	+	1	88	6	
SIX1	YES	14	14	1,508,020	1,508,874	-	0	284	8	
SIX2	YES	14	14	1,515,507	1516205	+	0	232	8	
SIX3	YES	14	14	1,620,196	1,620,687	-	0	163	3	
SIX5	YES	14	14	1,621,885	1,622,410	+	3	119	7	
SIX13	YES	14	14	1,721,250	1,722,191	+	1	293	12	
SIX10	YES	14	14	1,929,603	1,930,122	-	1	149	2	
SIX12	YES	14	14	1,931,346	1,931,777	-	1	127	10	
SIX7	YES	14	14	1,934,097	1,934,727	+	1	163	2	
FOXGR_015522	YES	14	14	1,996,473	1,996,874	-	2	79	9	
SIX13	YES	14	14	2,018,772	2,019,713	+	1	293	12	
FOXGR_015533 *	YES	14	14	2,033,852	2,034,196	-	0	114	3	Fol-EC3
FOXG_10949 *	NO	1	0	498,344	498,719	-	1	107	8	hydrophobin
FOXG_10950 *	NO	1	0	500,457	501,074	+	3	152	9	hydrophobin
FOXG_11033 *	NO	1	0	722,540	723,369	-	3	226	0	
FOXG_05750	NO	2	53	540,805	541,602	+	0	265	12	LysM domain x2
FOXG_05755 *	NO	2	53	549,873	550,346	+	0	157	3	
FOXG_18699 *	NO	4	2	3,264,675	3,264,965	+	0	96	10	
FOXGR_007323 *	NO	5	3	2,372,326	2,372,640	-	1	86	0	
FOXG_10672	NO	7	46	413,827	414,423	+	0	198	13	PAN/Apple domain x2
FOXG_04863 *	NO	7	46	3,884,224	3,885,126	+	0	300	8	
FOXG_04805 *	NO	7	46	4,051,134	4,051,586	+	1	132	8	
FOXG_02829 *	NO	8	50	561,065	561,514	-	0	149	16	
FOXGR_021626 *	NO	8	50	3,119,908	3,120,893	+	2	294	3	Glucanase—Fol-EC14
SIX8	YES	8	50	4,122,866	4,123,541	-	2	141	2	
PSL1 *	YES	8	50	4,124,187	4,124,677	+	3	111	9	
FOXG_08899 *	NO	9	5	3,212,288	3,212,782	+	1	148	1	
SIX8	YES	10	7	1363	1768	+	2	141	2	
FOXGR_010884	NO	10	7	444,492	445,286	+	0	264	0	
FOXG_11745 *	NO	10	7	655,322	655,924	+	1	183	4	phospholipase A2
SIX8	YES	10	7	3,412,328	3,413,003	-	2	141	2	
PSL1 *	YES	10	7	3,413,649	3,414,139	+	3	111	9	
FOXG_10138	NO	11	61	398,337	399,122	+	0	261	2	peptidase G1 family
FOXGR_025639 *	NO	11	61	507,487	507,728	+	1	61	2	
FOXG_16600 *	NO	11	61	2,173,630	2,174,176	-	1	164	0	
FOXG_13233 *	NO	12	12	668,427	668,929	+	2	131	4	ribonuclease F1—Fol-EC19
FOXG_13248 *	NO	12	12	703,080	703,929	+	2	248	6	Trypsin—Fol-EC20
FOXG_14607 *	NO	12	12	1,873,367	1,874,245	+	1	275	7	metalloprotease MEP1
FOXG_14684 *	NO	12	12	2,089,656	2,090,270	-	2	168	14	
PSL1 *	YES	-	9	576	1066	-	3	111	9	
SIX8	YES	-	10	224	783	+	2	141	2	
PSL1 *	YES	-	44	18,988	19,478	-	3	111	9	
SIX8	YES	-	44	20,124	20,683	+	2	141	2	
PSL1 *	YES	-	45	6024	6515	-	3	111	9	
PSL1 *	YES	-	58	1,499,796	1,500,286	-	3	111	9	
SIX8	YES	-	58	1,500,932	1,501,607	+	2	141	2	
PSL1 *	YES	-	59	3963	4453	-	3	111	9	
SIX8	YES	-	59	5099	5658	+	2	141	2	
PSL1 *	YES	-	60	7085	7575	-	3	111	9	
SIX8	YES	-	60	8221	8896	+	2	141	2	

* Novel candidate effector genes that were tested for cell-death induction and the suppression of Bax-induced cell death in *N. benthamiana*.

## Data Availability

The data that support the findings of this study are available from the corresponding author upon request and RNA-seq data are available in NCBI BioProject ID: PRJNA841073.

## Supplementary Materials

The following supporting information can be downloaded at: https://www.mdpi.com/article/10.3390/jof8070672/s1. Table S1. Primers used in this study; Table S2. Summary of RNA-seq statistics for three replicates of three infection time points (2, 4, and 6 dpi) and mycelium grown in vitro; Table S3. List of predicted proteins encoded by genes annotated from the PacBio-sequenced Fol4287 genome assembly; Table S4. Summary comparison of the Fol4287 genome assemblies and annotations obtained with Sanger and PacBio sequencing platforms. Figure S1. Whole-genome synteny analysis of predicted genes between Sanger-sequenced Fol4287 (*Fol* 2010) and PacBio-sequenced Fol4287 (*Fol* 2020) assemblies; Figure S2. Whole-genome alignment between Sanger-sequenced Fol4287 (*Fol* 2010) and PacBio-sequenced Fol4287 (*Fol* 2020) genome assemblies; Figure S3. RT-PCR to check the expression of 22 cloned effector candidate genes in agroinfiltrated *N. benthamiana* leaves; Figure S4. *FolEC-14* and *Eol-EC20* are not able to inhibit *L6TIR*- and *Fol-EC19*-induced cell death in *N. benthamiana* leaves; Figure S5. PaGluc131A alignment; Figure S6. Fol-EC14 alignment; Figure S7. ChGluc131A alignment; Figure S8/ChGluc131B alignment.

## References

[1] Zhou J.-M., Zhang Y. (2020). Plant Immunity: Danger perception and signaling. Cell.

[2] Lo Presti L., Lanver D., Schweizer G., Tanaka S., Liang L., Tollot M., Zuccaro A., Reissmann S., Kahmann R. (2015). Fungal effectors and plant susceptibility. Annu. Rev. Plant Biol..

[3] Giraldo M., Valent B. (2013). Filamentous plant pathogen effectors in action. Nat. Rev. Genet..

[4] van der Burgh A.M., Joosten M. (2019). Plant Immunity: Thinking outside and inside the box. Trends Plant Sci..

[5] Thomma B.P., Nurnberger T., Joosten M.H. (2011). Of PAMPs and effectors: The blurred PTI-ETI dichotomy. Plant Cell.

[6] Blondeau K., Blaise F., Graille M., Kale S.D., Linglin J., Ollivier B., Labarde A., Lazar N., Daverdin G., Balesdent M.H. (2015). Crystal structure of the effector AvrLm4-7 of *Leptosphaeria maculans* reveals insights into its translocation into plant cells and recognition by resistance proteins. Plant J..

[7] Plissonneau C., Daverdin G., Ollivier B., Blaise F., Degrave A., Fudal I., Rouxel T., Balesdent M. (2016). A game of hide and seek between avirulence genes *AvrLm4-7* and *AvrLm3* in *Leptosphaeria maculans*. New Phytol..

[8] Ghanbarnia K., Ma L., Larkan N.J., Haddadi P., Fernando W.G.D., Borhan M.H. (2018). *Leptosphaeria maculans* AvrLm9: A new player in the game of hide and seek with AvrLm4-7. Mol. Plant Pathol..

[9] Bourras S., McNally K.E., Ben-David R., Parlange F., Roffler S., Praz C.R., Oberhaensli S., Menardo F., Stirnweis D., Frenkel Z. (2015). Multiple avirulence loci and allele-specific efector recognition control the Pm3 race-specific resistance of wheat to powdery mildew. Plant Cell.

[10] Houterman P.M., Cornelissen B.J.C., Rep M. (2008). Suppression of plant resistance gene-based immunity by a fungal effector. PLoS Pathog..

[11] Ramachandran S.R., Yin C., Kud J., Tanaka K., Mahoney A.K., Xiao F., Hulbert S.H. (2017). Effectors from wheat rust fungi suppress multiple plant defense responses. Phytopathology.

[12] Selin C., de Kievit T.R., Belmonte M.F., Fernando W.G. (2016). Elucidating the role of effectors in plant-fungal interactions: Progress and challenges. Front. Microbiol..

[13] Nemri A., Saunders D.G., Anderson C., Upadhyaya N.M., Win J., Lawrence G.J., Jones D.A., Kamoun S., Ellis J.G., Dodds P.N. (2014). The genome sequence and effector complement of the flax rust pathogen *Melampsora lini*. Front. Plant Sci..

[14] Guyon K., Balagué C., Roby D., Raffaele S. (2014). Secretome analysis reveals effector candidates associated with broad host range necrotrophy in the fungal plant pathogen *Sclerotinia sclerotiorum*. BMC Genom..

[15] Haddadi P., Ma L., Wang H., Borhan M.H. (2016). Genome-wide transcriptomic analyses provide insights into the lifestyle transition and effector repertoire of *Leptosphaeria maculans* during the colonization of *Brassica napus* seedlings. Mol. Plant Pathol..

[16] Pérez-López E., Hossain M., Tu J., Waldner M., Todd C.D., Kusalik A.J., Wei Y., Bonham-Smith P.C. (2020). Transcriptome analysis identifies *Plasmodiophora brassicae* secondary infection effector candidates. J. Eukaryot. Microbiol..

[17] Kanja C., Hammond-Kosack K.E. (2020). Proteinaceous effector discovery and characterization in filamentous plant pathogens. Mol. Plant Pathol..

[18] Petre B., Saunders D.G.O., Sklenar J., Lorrain C., Krasileva K., Win J., Duplessis S., Kamoun S. (2016). Heterologous expression screens in *Nicotiana benthamiana* identify a candidate effector of the wheat yellow rust pathogen that associates with processing bodies. PLoS ONE.

[19] Lacomme C., Santa Cruz S. (1999). Bax-induced cell death in tobacco is similar to the hypersensitive response. Proc. Natl. Acad. Sci. USA.

[20] Wang Q., Han C., Ferreira A.O., Yu X., Ye W., Tripathy S., Kale S.D., Gu B., Sheng Y., Sui Y. (2021). Transcriptional programming and functional interactions within the *Phytophthora sojae* RXLR effector repertoire. Plant Cell.

[21] Wang Y., Li J., Xiang S., Zhou J., Peng X., Hai Y., Wang Y., Li S., Wei S. (2020). A putative effector UvHrip1 inhibits BAX-triggered cell death in *Nicotiana benthamiana*, and infection of *Ustilaginoidea virens* suppresses defense-related genes expression. PeerJ.

[22] Cheng Y., Wu K., Yao J., Li S., Wang X., Huang L., Kang Z. (2017). PSTha5a23, a candidate effector from the obligate biotrophic pathogen *Puccinia striiformis* f. sp. *tritici*, is involved in plant defense suppression and rust pathogenicity. Environ. Microbiol..

[23] Xu Q., Tang C., Wang X., Sun S., Zhao J., Kang Z., Wang X. (2019). An effector protein of the wheat stripe rust fungus targets chloroplasts and suppresses chloroplast function. Nat. Commun..

[24] Dutra D., Agrawal N., Ghareeb H., Schirawski J. (2020). Screening of Secreted Proteins of *Sporisorium reilianum* f. sp. *zeae* for Cell Death Suppression in *Nicotiana benthamiana*. Front. Plant Sci..

[25] Zhang N., Yang J., Fang A., Wang J., Li D., Li Y., Wang S., Cui F., Yu J., Liu Y. (2020). The essential effector SCRE1 in *Ustilaginoidea virens* suppresses rice immunity via a small peptide region. Mol. Plant Pathol..

[26] Derevnina L., Dagdas Y.F., de la Concepcion J.C., Białas A., Kellner R., Petre B., Domazakis E., Du J., Wu C.-H., Lin X. (2016). Nine things to know about elicitins. New Phytol..

[27] Li Y., Han Y., Qu M., Chen J., Chen X., Geng X., Wang Z., Chen S. (2020). Apoplastic cell death-inducing proteins of filamentous plant pathogens: Roles in plant-pathogen interactions. Front. Genet..

[28] Takken F., Rep M. (2010). The arms race between tomato and *Fusarium oxysporum*. Mol. Plant Pathol..

[29] Olivain C., Humbert C., Nahalkova J., Fatehi J., L’Haridon F., Alabouvette C. (2006). Colonization of tomato root by pathogenic and nonpathogenic *Fusarium oxysporum* strains inoculated together and separately into the soil. Appl. Environ. Microbiol..

[30] Rep M., Van Der Does H.C., Meijer M., Van Wijk R., Houterman P.M., Dekker H.L., De Koster C.G., Cornelissen B.J.C. (2004). A small, cysteine-rich protein secreted by *Fusarium oxysporum* during colonization of xylem vessels is required for *I-3*-mediated resistance in tomato. Mol. Microbiol..

[31] Houterman P.M., Speijer D., Dekker H.L., de Koster C.G., Cornelissen B.J.C., Rep M. (2007). The mixed xylem sap proteome of *Fusarium oxysporum*-infected tomato plants. Mol. Plant Pathol..

[32] Schmidt S.M., Houterman P.M., Schreiver I., Ma L., Amyotte S., Chellappan B., Boeren S., Takken F.L.W., Rep M. (2013). MITEs in the promoters of effector genes allow prediction of novel virulence genes in *Fusarium oxysporum*. BMC Genom..

[33] Ma L.-J., Van Der Does H.C., Borkovich K.A., Coleman J.J., Daboussi M.-J., Di Pietro A., Dufresne M., Freitag M., Grabherr M., Henrissat B. (2010). Comparative genomics reveals mobile pathogenicity chromosomes in *Fusarium*. Nature.

[34] Li J., Fokkens L., Conneely L.J., Rep M. (2020). Partial pathogenicity chromosomes in *Fusarium oxysporum* are sufficient to cause disease and can be horizontally transferred. Environ. Microbiol..

[35] Gawehns F., Ma L., Bruning O., Houterman P.M., Boeren S., Cornelissen B.J.C., Rep M., Takken F.L.W. (2015). The effector repertoire of *Fusarium oxysporum* determines the tomato xylem proteome composition following infection. Front. Plant Sci..

[36] Houterman P.M., Ma L., van Ooijen G., de Vroomen M.J., Cornelissen B.J., Takken F.L., Rep M. (2009). The effector protein Avr2 of the xylem-colonizing fungus *Fusarium oxysporum* activates the tomato resistance protein I-2 intracellularly. Plant J..

[37] Ma L., Houterman P.M., Gawehns F., Cao L., Sillo F., Richter H., Clavijo-Ortiz M.J., Schmidt S.M., Boeren S., Vervoort J. (2015). The *AVR2-SIX5* gene pair is required to activate *I-2*-mediated immunity in tomato. New Phytol..

[38] Gawehns F., Houterman P.M., Ichou F.A., Michielse C.B., Hijdra M., Cornelissen B.J., Rep M., Takken F.L.W. (2014). The *Fusarium oxysporum* effector Six6 contributes to virulence and suppresses I-2-mediated cell death. Mol. Plant Microbe Interact..

[39] Vlaardingerbroek I., Beerens B., Rose L., Fokkens L., Cornelissen B.J.C., Rep M. (2016). Exchange of core chromosomes and horizontal transfer of lineage-specific chromosomes in *Fusarium oxysporum*. Environ. Microbiol..

[40] Ayukawa Y., Asai S., Gan P., Tsushima A., Ichihashi Y., Shibata A., Komatsu K., Houterman P.M., Rep M., Shirasu K. (2021). A pair of effectors encoded on a conditionally dispensable chromosome of *Fusarium oxysporum* suppress host-specific immunity. Commun. Biol..

[41] Gonzalez-Cendales Y., Catanzariti A.-M., Baker B., Mcgrath D.J., Jones D.A. (2015). Identification of I-7 expands the repertoire of genes for resistance to *Fusarium* wilt in tomato to three resistance gene classes. Mol. Plant Pathol..

[42] Simons G., Groenendijk J., Wijbrandi J., Reijans M., Groenen J., Diergaarde P., van der Lee T., Bleeker M., Onstenk J., de Both M. (1998). Dissection of the fusarium *I2* gene cluster in tomato reveals six homologs and one active gene copy. Plant Cell.

[43] Catanzariti A.M., Lim G.T.T., Jones D.A. (2015). The tomato *I-3* gene: A novel gene for resistance to *Fusarium* wilt disease. New Phytol..

[44] Catanzariti A.M., Do H.T., Bru P., de Sain M., Thatcher L.F., Rep M., Jones D.A. (2017). The tomato *I* gene for *Fusarium* wilt resistance encodes an atypical leucine-rich repeat receptor-like protein whose function is nevertheless dependent on SOBIR1 and SERK3/BAK1. Plant J..

[45] Jenkins S., Taylor A., Jackson A.C., Armitage A.D., Bates H.J., Mead A., Harrison R.J., Clarkson J.P. (2021). Identification and Expression of Secreted *In Xylem* Pathogenicity Genes in *Fusarium oxysporum* f. sp. pisi. Front. Microbiol..

[46] Vleeshouwers V.G., Oliver R.P. (2014). Effectors as tools in disease resistance breeding against biotrophic, hemibiotrophic, and necrotrophic plant pathogens. Mol. Plant Microbe Interact..

[47] Domazakis E., Lin X., Aguilera-Galvez C., Wouters D., Bijsterbosch G., Wolters P.J., Vleeshouwers V.G.A.A., Shan L., He P. (2017). Effectoromics-Based Identification of Cell Surface Receptors in Potato. Plant Pattern Recognition Receptors: Methods and Protocols.

[48] Mes J.J., Wit R., Testerink C.S., de Groot F., Haring M.A., Cornelissen B.J. (1999). Loss of avirulence and reduced pathogenicity of a gamma-irradiated mutant of *Fusarium oxysporum* f. sp. *lycopersici*. Phytopathology.

[49] Ye J., Coulouris G., Zaretskaya I., Cutcutache I., Rozen S., Madden T.L. (2012). Primer-BLAST: A tool to design target-specific primers for polymerase chain reaction. BMC Bioinform..

[50] Rao X., Huang X., Zhou Z., Lin X. (2013). An improvement of the 2^(-delta delta CT) method for quantitative real-time polymerase chain reaction data analysis. Biostat. Bioinforma. Biomath..

[51] Kim D., Paggi J.M., Park C., Bennett C., Salzberg S.L. (2019). Graph-based genome alignment and genotyping with HISAT2 and HISAT-genotype. Nat. Biotechnol..

[52] Shao M., Kingsford C. (2017). Accurate assembly of transcripts through phase-preserving graph decomposition. Nat. Biotechnol..

[53] Testa A.C., Hane J.K., Ellwood S.R., Oliver R.P. (2015). CodingQuarry: Highly accurate hidden Markov model gene prediction in fungal genomes using RNA-seq transcripts. BMC Genom..

[54] Hoff K.J., Lomsadze A., Borodovsky M., Stanke M. (2019). Whole-Genome Annotation with BRAKER. Methods Mol. Biol..

[55] Waterhouse R.M., Seppey M., Simão F.A., Manni M., Ioannidis P., Klioutchnikov G., Kriventseva E.V., Zdobnov E.M. (2018). BUSCO Applications from Quality Assessments to Gene Prediction and Phylogenomics. Mol. Biol. Evol..

[56] Wang Y.P., Tang H.B., DeBarry J.D., Tan X., Li J.P., Wang X.Y., Lee T.-H., Jin H., Marler B., Guo H. (2012). MCScanX: A toolkit for detection and evolutionary analysis of gene synteny and collinearity. Nucleic Acids Res..

[57] Johnson M., Zaretskaya I., Raytselis Y., Merezhuk Y., McGinnis S., Madden T.L. (2008). NCBI BLAST: A better web interface. Nucleic Acids Res..

[58] Kurtz S., Phillippy A., Delcher A.L., Smoot M., Shumway M., Antonescu C., Salzberg S.L. (2004). Versatile and open software for comparing large genomes. Genome Biol..

[59] Chen C., Chen H., Zhang Y., Thomas H.R., Frank M.H., He Y., Xia R. (2020). TBtools: An integrative toolkit developed for interactive analyses of big biological data. Mol. Plant.

[60] Neu E., Debener T. (2019). Prediction of the *Diplocarpon rosae* secretome reveals candidate genes for effectors and virulence factors. Fungal Biol..

[61] Armenteros A.J.J., Tsirigos K.D., Sønderby C.K., Petersen T.N., Winther O., Brunak S., Von Heijne G., Nielsen H. (2019). SignalP 5.0 improves signal peptide predictions using deep neural networks. Nat. Biotechnol..

[62] Krogh A., Larsson B., von Heijne G., Sonnhammer E.L. (2001). Predicting transmembrane protein topology with a hidden Markov model: Application to complete genomes. J. Mol. Biol..

[63] Emanuelsson O., Nielsen H., Brunak S., von Heijne G. (2000). Predicting subcellular localization of proteins based on their N-terminal amino acid sequence. J. Mol. Biol..

[64] de Castro E., Sigrist C.J.A., Gattiker A., Bulliard V., Langendijk-Genevaux P.S., Gasteiger E., Bairoch A., Hulo N. (2006). ScanProsite: Detection of PROSITE signature matches and ProRule-associated functional and structural residues in proteins. Nucleic Acids Res..

[65] Pierleoni A., Martelli P.L., Casadio R. (2008). PredGPI: A GPI-anchor predictor. BMC Bioinform..

[66] Patro R., Duggal G., Love M.I., Irizarry R.A., Kingsford C. (2017). Salmon provides fast and bias-aware quantification of transcript expression. Nat. Methods.

[67] Love M.I., Huber W., Anders S. (2014). Moderated estimation of fold change and dispersion for RNA-seq data with DESeq2. Genome. Biol..

[68] Hellens R.P., Allan A.C., Friel E.N., Bolitho K., Grafton K., Templeton M.D., Karunairetnam S., Gleave A.P., Laing W.A. (2005). Transient expression vectors for functional genomics, quantification of promoter activity and RNA silencing in plants. Plant Methods.

[69] de Rybel B., van den Berg W., Lokerse A., Liao C.Y., van Mourik H., Moller B., Peris C.L., Weijers D. (2011). A versatile set of ligation-independent cloning vectors for functional studies in plants. Plant Physiol..

[70] Ma L., Lukasik E., Gawehns F., Takken F.L.W. (2012). The use of agroinfiltration for transient expression of plant resistance and fungal effector proteins in *Nicotiana benthamiana* leaves. Methods Mol. Biol..

[71] Ma L., Djavaheri M., Wang H., Larkan N.J., Haddadi P., Beynon E., Gropp G., Borhan M.H. (2018). *Leptosphaeria maculans* effector protein AvrLm1 modulates plant immunity by enhancing MAP kinase 9 phosphorylation. iScience.

[72] Yin W., Wang Y., Chen T., Lin Y., Luo C. (2018). Functional Evaluation of the Signal Peptides of Secreted Proteins. BIO Protoc..

[73] Sperschneider J., Dodds P.N., Gardiner D.M., Singh K.B., Taylor J.M. (2018). Improved prediction of fungal effector proteins from secretomes with EffectorP 2.0. Mol. Plant Pathol..

[74] Coll N.S., Epple P., Dangl J.L. (2020). Programmed cell death in the plant immune system. Cell Death Differ..

[75] Bernoux M., Ve T., Williams S., Warren C., Hatters D., Valkov E., Zhang X., Ellis J.G., Kobe B., Dodds P.N. (2011). Structural and functional analysis of a plant resistance protein TIR domain reveals interfaces for self-association, signaling, and autoregulation. Cell Host Microbe.

[76] Hogenhout S.A., Van der Hoorn R.A.L., Terauchi R., Kamoun S. (2009). Emerging concepts in effector biology of plant-associated organisms. Mol. Plant Microbe Interact..

[77] Lievens B., Houterman P.M., Rep M. (2009). Effector gene screening allows unambiguous identification of *Fusarium oxysporum* f. sp. *lycopersici* races and discrimination from other *formae speciales*. FEMS Microbiol Lett..

[78] Lafond M., Navarro D., Haon M., Couturier M., Berrin J.G. (2012). Characterization of a broad-specificity beta-glucanase acting on beta-(1,3)-, beta-(1,4)-, and beta-(1,6)-glucans that defines a new glycoside hydrolase family. Appl. Environ. Microbiol..

[79] Miyazaki T., Yoshida M., Tamura M., Tanaka Y., Umezawa K., Nishikawa A., Tonozuka T. (2013). Crystal structure of the N-terminal domain of a glycoside hydrolase family 131 protein from *Coprinopsis cinerea*. FEBS Lett..

[80] Anasontzis G.E., Lebrun M.H., Haon M., Champion C., Kohler A., Lenfant N., Martin F., O’Connell R.J., Riley R., Grigoriev I.V. (2019). Broad-specificity GH131 beta-glucanases are a hallmark of fungi and oomycetes that colonize plants. Environ. Microbiol..

[81] Schmidt A., Jelsch C., Ostergaard P., Rypniewski W., Lamzin V.S. (2003). Trypsin revisited: Crystallography AT (SUB) atomic resolution and quantum chemistry revealing details of catalysis. J. Biol. Chem..

[82] Rypniewski W., Hastrup S., Betzel C., Dauter M., Dauter Z., Papendorf G., Branner S., Wilson K. (1993). The sequence and X-ray structure of the trypsin from *Fusarium oxysporum*. Protein Eng..

[83] Stork I., Gartemann K.H., Burger A., Eichenlaub R. (2008). A family of serine proteases of *Clavibacter michiganensis* subsp. *michiganensis*: *ChpC* plays a role in colonization of the host plant tomato. Mol. Plant Pathol..

[84] Chalupowicz L., Barash I., Reuven M., Dror O., Sharabani G., Gartemann K.H., Eichenlaub R., Sessa G., Manulis-Sasson S. (2017). Differential contribution of *Clavibacter michiganensis* ssp. *michiganensis* virulence factors to systemic and local infection in tomato. Mol. Plant Pathol..

[85] Jahr H., Dreier J., Meletzus D., Bahro R., Eichenlaub R. (2020). The endo-beta-1,4-glucanase CelA of *Clavibacter michiganensis* subsp. *michiganensis* is a pathogenicity determinant required for induction of bacterial wilt of tomato. Mol. Plant Microbe Interact..

[86] Sanchez L.M., Ohno Y., Miura Y., Kawakita K., Doke N. (1992). Host Selective Suppression by Water-Soluble Glucans from *Phytophthora* spp. of Hypersensitive Cell Death of Suspension-Cultures Cells from Some Solanaceous Plants Caused by Hyphal Wall Elicitors of the Fungi. Jpn. J. Phytopathol..

[87] Ali S., Magne M., Chen S., Côté O., Stare B.G., Obradovic N., Jamshaid L., Wang X., Bélair G., Moffett P. (2015). Analysis of putative apoplastic effectors from the nematode, *Globodera rostochiensis*, and identification of an expansin-like protein that can induce and suppress host defenses. PLoS ONE.

[88] Carlile A.J., Bindschedler L.V., Bailey A.M., Bowyer P., Clarkson J.M., Cooper R.M. (2000). Characterization of SNP1, a cell wall-degrading trypsin, produced during infection by *Stagonospora nodorum*. Mol. Plant. Microbe Interact..

[89] Hao G., McCormick S., Vaughan M.M., Naumann T.A., Kim H.-S., Proctor R., Kelly A., Ward T.J. (2019). *Fusarium graminearum* arabinanase (Arb93B) enhances wheat head blight susceptibility by suppressing plant immunity. Mol. Plant Microbe Interact..

[90] Hao G., McCormick S., Usgaard T., Tiley H., Vaughan M.M. (2020). Characterization of three *Fusarium graminearum* effectors and their roles during *Fusarium* head blight. Front. Plant Sci..

[91] Rose J.K., Ham K.S., Darvill A.G., Albersheim P. (2002). Molecular cloning and characterization of glucanase inhibitor proteins: Coevolution of a counterdefense mechanism by plant pathogens. Plant Cell.

[92] Bozkurt T.O., Schornack S., Banfield M.J., Kamoun S. (2012). Oomycetes, effectors, and all that jazz. Curr. Opin. Plant Biol..

[93] Yang B., Wang Y., Tian M., Dai K., Zheng W., Liu Z., Yang S., Liu X., Shi D., Zhang H. (2021). Fg12 ribonuclease secretion contributes to *Fusarium graminearum* virulence and induces plant cell death. J. Integr. Plant Biol..

