# Supplementary material for: Transcriptome Analysis of Fusarium–Tomato Interaction Based on an Updated Genome Annotation of Fusarium oxysporum f. sp. lycopersici Identifies Novel Effector Candidates That Suppress or Induce Cell Death in Nicotiana benthamiana
Source: J Fungi (Basel). 2022 Jun 26;8(7):672. doi: 10.3390/jof8070672 (PMC9316272; doi:10.3390/jof8070672)
Supplement: Supplementary file 1 [file jof-08-00672-s001.zip › Supplementary Materials/Supplementary Figures/Supplementary Figure S1.pdf]

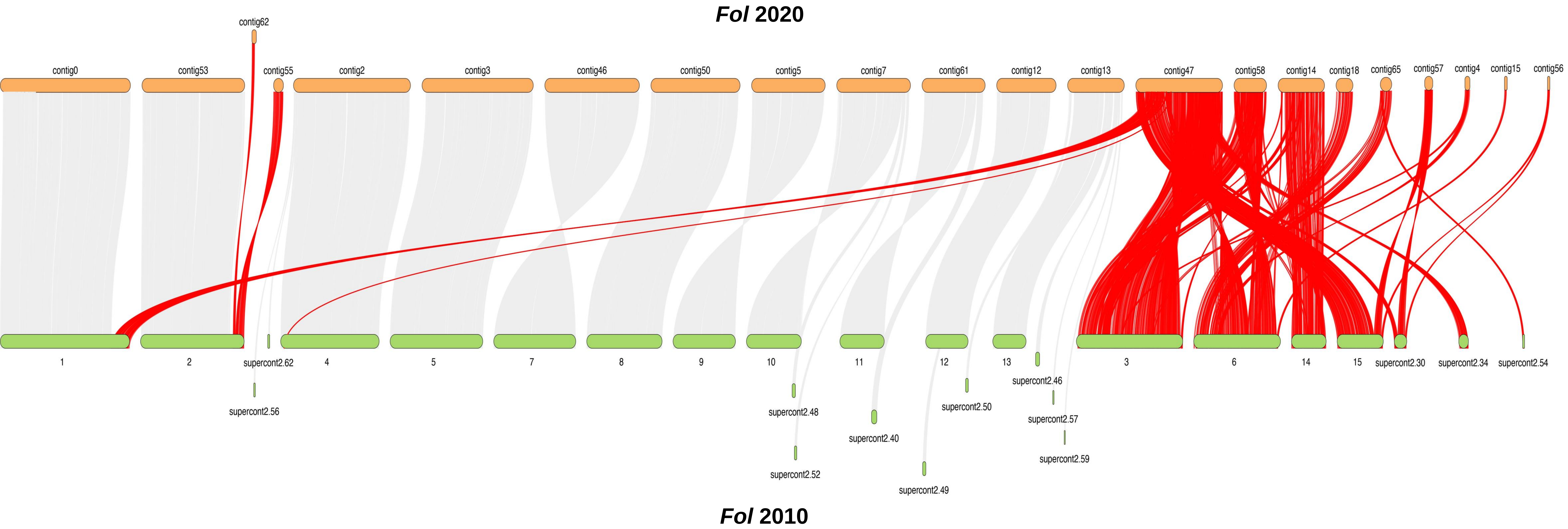

Supplementary Figure S1. Whole-genome synteny analysis of predicted genes between Sanger-sequenced Fol4287 (*Fol* 2010) and PacBio-sequenced Fol4287 (*Fol* 2020) assemblies. The grey lines in the background indicate the collinear genes between the two assemblies, while the red lines highlight the collinear genes in the lineage-specific regions.
