# Supplementary material for: Transcriptome Analysis of Fusarium–Tomato Interaction Based on an Updated Genome Annotation of Fusarium oxysporum f. sp. lycopersici Identifies Novel Effector Candidates That Suppress or Induce Cell Death in Nicotiana benthamiana
Source: J Fungi (Basel). 2022 Jun 26;8(7):672. doi: 10.3390/jof8070672 (PMC9316272; doi:10.3390/jof8070672)
Supplement: Supplementary file 1 [file jof-08-00672-s001.zip › Supplementary Materials/Supplementary Figures/Supplementary Figure S2.pdf]

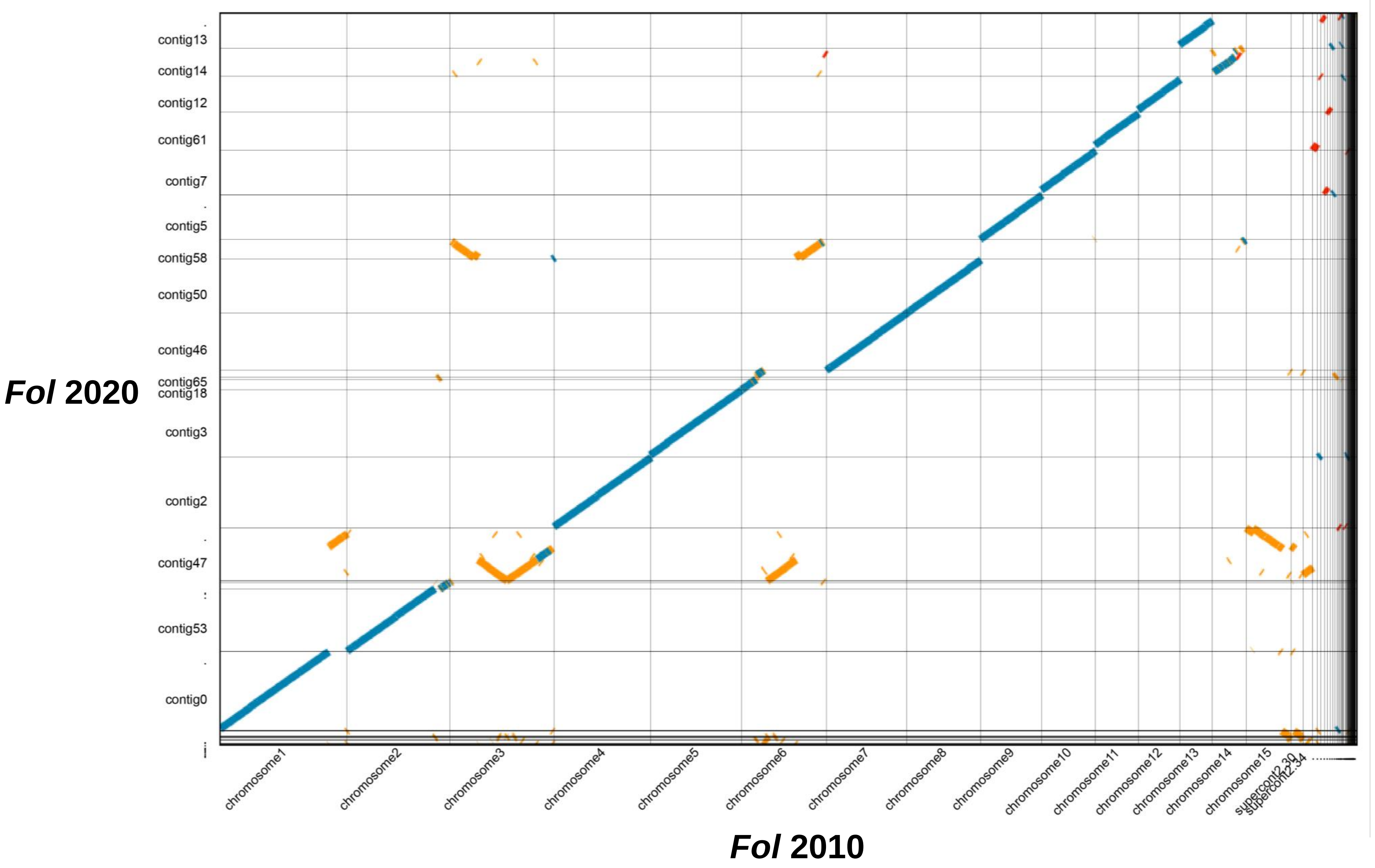

Supplementary Figure S2. Whole-genome alignment between Sanger-sequenced Fol4287 (*Fol* 2010) and PacBio-sequenced Fol4287 (*Fol* 2020) genome assemblies. The alignment shows the correspondence between chromosomes in *Fol* 2010 genome assembly and contigs in *Fol* 2020 genome assembly. Unique forward alignments are in blue, unique reverse alignments are in red and repetitive
