# Supplementary material for: Transcriptome Analysis of Fusarium–Tomato Interaction Based on an Updated Genome Annotation of Fusarium oxysporum f. sp. lycopersici Identifies Novel Effector Candidates That Suppress or Induce Cell Death in Nicotiana benthamiana
Source: J Fungi (Basel). 2022 Jun 26;8(7):672. doi: 10.3390/jof8070672 (PMC9316272; doi:10.3390/jof8070672)
Supplement: Supplementary file 1 [file jof-08-00672-s001.zip › Supplementary Materials/Supplementary Figures/Supplementary Figure S3.pdf]

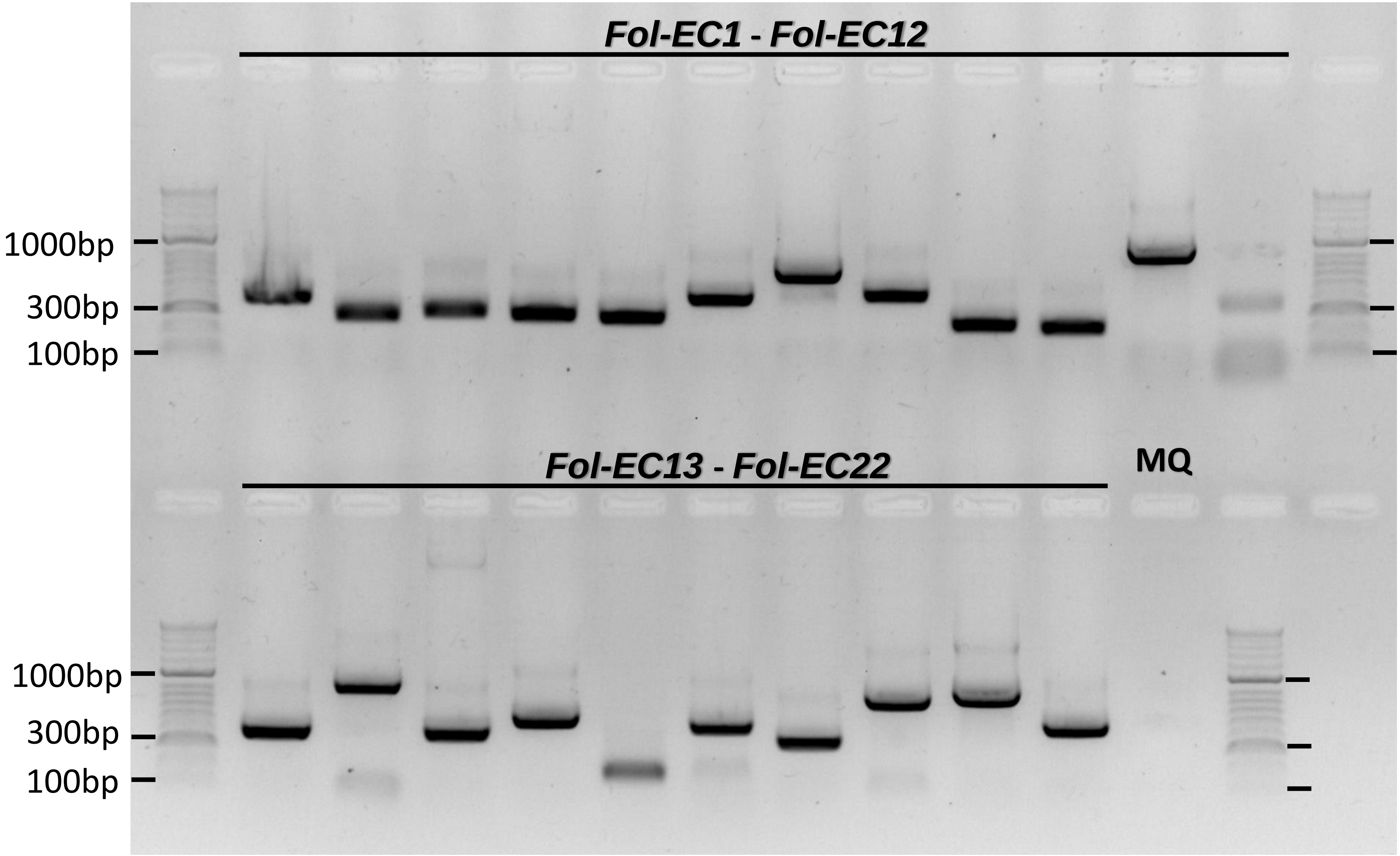

Supplementary Figure S3. RT-PCR to check the expression of 22 cloned effector candidate genes in agroinfiltrated *N. benthamiana* leaves. All cloned *Fol-EC1* to *EC22* genes were transiently expressed in *N. benthamaian* leaves and RNA was extracted for RT-PCR with the corresponding LIC cloning primer combinations as shown in table S1. MQ indicates water control.
