# Supplementary material for: Transcriptome Analysis of Fusarium–Tomato Interaction Based on an Updated Genome Annotation of Fusarium oxysporum f. sp. lycopersici Identifies Novel Effector Candidates That Suppress or Induce Cell Death in Nicotiana benthamiana
Source: J Fungi (Basel). 2022 Jun 26;8(7):672. doi: 10.3390/jof8070672 (PMC9316272; doi:10.3390/jof8070672)
Supplement: Supplementary file 1 [file jof-08-00672-s001.zip › Supplementary Materials/Supplementary Figures/Supplementary Figure S5.pdf]

**Signal peptide**

```
Colchl   1 -MKFTW---NI-ASAALLA-----TRA---QCEILWDGRFNDMTASTDLDKWSFGNPVGS
Colhig   1 -MKFTW---SI-ASAALLA-----TRA---QCEILWDGRFNDMTASTDLDKWSFSNAVGP
Colinc   1 -MKFTW---SI-ASAALLA-----TRV---QCEILWDGRFNDMTSSTDLDKWSFSSPVGS
Colsim   1 -MKFTW---SI-ASAALLA-----TRA---QCEILWDGRFNDMTSATDLDKWSWSSQVGP
Veralf   1 MMKFLA---TA-SAAFSLV-----SQC---HSAVLWDGRFNDLTSSTDLDKWSWATQVGP
Verlon   1 MMKFLA---TA-SVAFSLV-----SQC---HSAVLWDGRFNDLTSSTDLNKWSWATQVGP
Verdah   1 -MKFLA---TA-SAAFSLV-----SQC---YSAILWDGRFNDLTSSTDLNKWSWATQVGP
Conlig   1 -MKSPT---LA-SLAAQLL-----CVS---AGTILWDGRFNDLTSSTDLNTWSWSNQVGP
Neucra   1 -MKFTL---LS-GLVAQAL-----SVS---AGSILWDGRFNDLTSATDLSKWSWSSQVGP
Neutet   1 -MKFTL---LS-GLVAKAL-----SVS---AGSILWDGRFNDLTSATDLSKWSWSSQVGP
Sormac   1 -MKFSV---LA-GLVAQAF-----TVS---AGTVLWDGRFNDFTSSTDLNKWSWGNQVGP
Madmyc   1 -MRFPV---LS-SLAAQVL-----SVS---AGTILWDGRFNDLSSSTDLNNWSWGNQVGP
Podans   1 -MKFHV---LS-GLVAQVL-----SVS---AGTILWDGRFNDMTSSADLNKWSWGNQVGP
Chaglo   1 -MKFSV---LS-SILANAL-----SVS---AGTILWDGRFNDLSSSTDLNNWSWGNQVGP
Thethe   1 -MRFSV---LS-SILANAL-----SAS---AGTILWDGRFNDLSSSKDLEKWSWANQVGP
Thiter   1 -MKFSI---LP-SLLAGVV-----SVS---AGTILWDGRFNDLSSSTDLNNWSWSNEVGP
Chathe   1 -MRLLT---LA-TLTAPAL-----------GGNILWDGRFNDLSSSLDLNRWSWSNQVGP
Gaetri   1 -MRFSVANVLA-GTAYMAS-----AVA---AGTVIWDGRFNDLTSSTDLNKWSWGSQVGP
Magory   1 -MRLSI---CT-AALSLTG-----GVV---AGTVIWDGRFNDMTSAADLNKWSWGNQVGS
Maggri   1 -MRLSI---FT-APLSLMG-----GAV---AGTVIWDGRFNDMTSAADLNKWSWGNQVGS
Stacha   1 -MKTVAAT-LL-AASSLWV-----GLA---SGAVLWDGRTNDMDSAEDLLDWSWGNQVGP
Stachl   1 -MKTVAAT-LL-AASSLWV-----GLA---SGAVLWDGRTNDMDSAEDLLDWSWGNQVGP
Micbol   1 -MKTSV---LV-SLFLGQL-----A-S---CGTVLWDGRFNDFTSASDLDKWSWGNQVGP
Colfio   1 -MKVTA---LL-TLVP---------LT---SAKVLWDGRFNDFNTSSDLNKWSWSNQVGP
Colnym   1 -MKVTA---LL-TLVP---------LT---SAKVLWDGRFNDFNTSSDLNKWSWSNQVGP
Colsal   1 -MKVTA---LL-TLLP---------LT---SAKVLWDGRFNDFNSSSDLNKWSWSNQVGP
Colgra   1 -MKTAA---LL-SLLP---------LA---SAKVLWDGRFNDLSSSTDLNNWSWSNQVGP
Colsub   1 -MKAAA---LL-SLLS---------LA---SAKVLWDGRFNDLSSSTDLNKWSWSNQVGP
Coltof   1 -MKAAA---LL-SLLP---------LT---SAKVLWDGRFNDLSSSSDLNKWSWANQVGP
Colorb   1 -MKAAA---LL-AVLP---------LA---SAKVLWDGRFNDLTSSTDLNKWSWANQVGP
Colglo   1 -MKAAA---LF-SLLP---------LA---SGKILWDGRFNDLNSSTDLNKWSWANQVGP
Phamin   1 -MQLTK------ALIA--------SLAALGNAAVIWDGRFNDFSSSSDLNKWSWSNQVGP
Pesfic   1 -MKAT-------ILLA--------SLA---KAAVLWDGRFNDFSSSADLNKWSWGNQVGP
Psevex   1 -MKTT-------LLFA--------QVA---SAAVIWDGRFNDFSSSADLDKWSWSNQVGP
Rosnec   1 -MKAST---LSAVLLV--------RLA---SAAIIWDGRFNEFTSSADLNKWSWSNQVGP
Daldsp   1 -MKVTL---F--SALA--------AVA---SAAVLWDGRFNDLGTSADLNKWSWSNQVGP
Hyposp   1 -MKAAL---I--SVLA--------TVA---SAAVLWDGRFNDLGTSADLNKWSWSNQVGP
Ophpic   1 -MK---------GLLALCL----AAAA---HAAVLWDGRFNEYSSAADINNWSWSNQVGP
Spobra   1 -MKLSN---SL-LLLT-------GTAA---QAAVLWDGRFNDLSSAADLNTWSWSNQVGP
Sposch   1 -MKLSN---SL-LLLA-------GTAA---QAAVLWDGRFNDLSSAADLNTWSWSNQVGP
Spoins   1 -MKLPG---SL-PLLASCLLPVFGGAA---RAAVLWDGRFNDLATASDLNKWSWSSQVGP
Lompro   1 -MKLSP---S--ALLALAP-----ALA---NAAVLWDGRFNDMTSSADLDKWSWSNQVGA
Sceapi   1 -MKLSP---S--ALLALAP-----ALV---NAAVIWDGRFNDLSSSADLDKWSWSNQVGA
Diaamp   1 -MRAFS---LS-AAAALLA-----TAN---AGTVIWDGRFNDLSTSADLATWSWSNQVGP
Neodit   1 -MKTFA---AF-ASLA--------GLA---AAEILWDGRFNDLESSADLADWSWSNQVGP
Eutlat   1 -MKAPT---LL-ALLS-------GHLAA--CGTVIWDGRFNDLESAADLDEWDWSNQVGP
Conlus   1 -MPSSSSS-LIASLVGLLS----ASILTVQAGTVLWDGRFNDLSSSTDLDDWSWSNEVGP
Cerpla   1 -MKSFA------SLLLL------GAPA---LASILWDGRFNDMTSSADLGKWSWSTPVGS
Thipun   1 -MKSFA------SLLFL------SAPA---LASILWDGRFNS-STAAELSQWSWSNQVGP
```

```
Colchl  48 YQYYIHGSGKTTEYVNLSEDFKNPADTVSKQGAKISLTSTAFWNGQNMRRTELIPETKAA
Colhig  48 YQYYIHGPGETTEYVNLSEDFKNPADTASKQGAKISLTSTAFWNGQNMRRTELIPQTKAA
Colinc  48 YQYYIHGSGKTTEYVNLSEDFKNPADTASKQGAKISLTSTAFWNGQNMRRTELIPQTSAA
Colsim  48 YQYYIHGSGATTDYVNLSSDFKNPADTGSKQGAKISLTSTSFWNGQTMRRTELIPQTTAA
Veralf  49 YQYYIHGSGPVTEYVNLSPSYKNPADSGSKQGVKISLTDTAYWNGQNMRRTELIPQTSAA
Verlon  49 YQYYIHGSGPVTEYVNLSPSYKNPADSGSKQGVKISLTDTAYWNGQNMRRTELIPQTSAA
Verdah  48 YQYYIHGSGPVTEYVNLSPSYKNPADSGSKQGVKISLTDTAYWNGQNMRRTELIPQTSAA
Conlig  48 YQYYIHGSSPVTAYVNLSPTYKNPADAGSKQGAKITLDSTSYWNGQTMRRTELIPQTAAP
Neucra  48 YQYYIHGPSEVTSYVNLSPSFKNPADSGSSQGAKITLDKTAFWNGQTMRRTELIPQTTAA
Neutet  48 YQYYIHGPSEVTSYVNLSPSFKNPADSGSSQGAKITLDKTAFWNGQTMRRTELIPQTTAA
Sormac  48 YQYYIHGSSEVTSYVNLSPSYKNPADSGSNQGAKITLDNTAYWNGQNMRRTELIPQTTAA
Madmyc  48 YQYYIHGPSEVTAYVNLSPDYKNPADSGSRQGAKITLDDTAYWNGQNMRRTELIPQTSAA
Podans  48 YQYYIHGSSPVSAYVNLSPDYKNPADTGSRQGAKITLDNTAYWNGQNMRRTELIPQTTAA
Chaglo  48 YQYYIHGSSSVTSYVNLSPDYKNPADSGSKQGAKITLDNTAYWNGQNMRRTELIPQTTAP
Thethe  48 YQWYIHGSSSVTAYVNLSPDYKNPADSGSKQGAKITLDNTAYWNGQNMRRTELIPQTSAA
Thiter  48 YQYYIHGPSPVTAYVNLSPDYKNPADTGSKQGAKITLDSTSYWNGQNMRRTELIPQTSAA
Chathe  45 YQYYIHGSSPVTAYVNLSPSYKNPFDAGSKQGAKITLDRTAYWNGQNMRRTELIPQTTAP
Gaetri  51 YQYYIHGSSPVASYINLSPSYKNPADAASRQGAKFTLDSTAYWNGQNMRRIELIPQTTAA
Magory  48 YQYYIHGSSPVTSYVNLSPSHKNPADTTSKQGAKLTLDSTAYWNGQNMRRIELIPQTTAA
Maggri  48 YQYYIHGSSPVASYVDLSPSHKNPADTTSKQGAKLTLDSTAYWNGQNMRRIELIPQTTAA
Stacha  50 YQWYIHGNGPVSEYVALSPDYKNPADSGSSKGIKISLTDTAYWNGQNMRRTELIPQTSAA
Stachl  50 YQWYIHGNGPVSEYVALSPDYKNPADSGSSKGIKISLTDTAYWNGQNMRRTELIPQTSAA
Micbol  47 YQWYIKGSGKTTEYVNLSPAYKNAADSASNQGVKISLTNSAYWNGQNMRRTELIPQTKAA
Colfio  44 YQYYIHGSGDVTKYVNLSPSYKNPADTASKQGVKITLDSTAYWNGQNMRRTELIPQTKAA
Colnym  44 YQYYIHGSGDVTKYVNLSPSYKNPADTASKQGVKITLDSTAYWNGQNMRRTELIPQTKAA
Colsal  44 YQYYIHGSGDVTKYVNLSPSYKNPADTASKQGVKITIDSTAYWNGQNMRRTELIPQTKAA
Colgra  44 YQYYIHGSGDVAKYVNLSPSYKNPSDAASKQGVKITLDSTAYWNGQNMRRTELIPQTKAA
Colsub  44 YQYYIHGSGDVTKYVNLSPSYKNPNDTASKQGVKITLDSTAYWNGQNMRRTEIIPQTKAA
Coltof  44 YQYYIHGSGDVTKYVNLSPSYKNPNDTASKQGVKITLDSTAYWNGQNMRRTELIPQTKAA
Colorb  44 YQYYIHGPGDVTKYVNLSPSYKNPADTASKQGVKITLDSTAYWNGQNMRRTELIPQTKAA
Colglo  44 YQYYIHGSGDVTKYVNLSPDYKNPADTASKQGVKITLDSTAYWNGQNMRRTELIPQTKAA
Phamin  46 YQYYIHGSGTVEKYVNLSPDYKNPNDTVSKQGAKFTLDSTAFWNGQNMRRTELIPQTTAG
Pesfic  42 YQYYIHGSGTVDKYINLGTSFKNPNDTSSKQGAKFTLDSTAYWNGQNMRRIELIPQTTAA
Psevex  42 YQYYIHGSGTVDKYVNLSPDYKNPNDTVSKQGAKFTLDSTAFWNGQTMRRTELIPQTTAA
Rosnec  46 YQYYIHGSSGVDRYVNLSPDYKNPADTASRQGAKFTLDSTAFWNGQNMRRTELIPQTKAA
Daldsp  44 YQYYIHGSGSVEKYINFSPDYKNPADTVSKQGAKFTLDSTAFWNGQNMRRIELIPQTKAA
Hyposp  44 YQYYIHGSGSVEKYINFSPDYKNPADKVSKQGAKFTLDSTAFWNGQNMRRIELIPQTKAA
Ophpic  44 YQYYIHGSGTVDEYIALSPDYKNPHDTVAKQGARFTLDSTAYWNGQNMRRIELIPQTKAA
Spobra  46 YQYYIHGSGTVNKYIALSPSYKNPNDTVSKQGARFTLDSTSFWNGQTMRRTELIPQTKAA
Sposch  46 YQYYIHGSGTVNKYIALSPSYKNPNDTVSKQGARFTLDSTSFWNGQTMRRTELIPQTKAA
Spoins  53 YQYYIHGSGTVDKYIALSPDYKNPNDTVSKQGARFTLDATSFWNGQTMRRTELIPQTKAA
Lompro  47 YQWYIKGSSATSAYINLSEDFKNPADTSSKQGAKFTLDGTAFWNGQTMRRTELIPQTTAG
Sceapi  47 YQWYIKGSSPTSSYINLSEDFKNPADTSSKKGAKFTLDGTAFWNGQTMRRTELIPQTTAG
Diaamp  48 YQYYIHGSGSVTEYVNLSPSYKNPADTSSKQGAKVTLTNTAYWNGQNMRRTELIPQTSAA
Neodit  45 YQYYIHGDGEVTEYVNLAADFANPADAGSSQGAKISLTDTAYWNGQTMRRTELIPQTSAA
Eutlat  47 YQWYIHGPSATADYVDLSADYKNPADESSTKGVKLTLDSTAFWNGQNMRRTELIPQTTAA
Conlus  55 YQYYIHGDGPVTEYINLSAAYKNPADTASAQGAQLTLDDTAYWEGQTMRRTELIPQTTAA
Cerpla  45 YQYYIHGSGEVTEYVNLSADYKNPADTGSNQGAKITLTNTAYWNGQNMRRTELIPQTTAA
Thipun  44 YQYYIHGSGDLSDYVDISTDFKNPGDSNSEYGAKISLTSTSYWEGQNMRRTELIPSTKAA
```

```
Colchl  108 IAKGKVYYHFSLKRSDVNAPSLNKEHQIAFFESHFVEMKSGWQSGAAGTEDPLLRWVVGG
Colhig  108 IAKGKVYYHFSLKRSDTNAPSLSKEHQIAFFESHFVEMKSGWQSGATGTEDPLLRWVVGG
Colinc  108 IAKGKVYYHFSLKRTDTNAPSLNKEHQIAFFESHFVEMKSGWQSGATGTEDPLLRWVVGG
Colsim  108 IGKGKLFYHFSLKRSDTNAPSLNKEHQIAFFESHFVEMKSGWQSGATGTEDPLLRWVVGG
Veralf  109 INKGKVFYHFSIKRSDVNPPATTREHQIAFFESHFTELKSGWLSGAAGTSDPLLRWCVGG
Verlon  109 INKGKVFYHFSIKRSDVNPPATTREHQIAFFESHFTELKSGWLSGAAGTSDPLLRWCVGG
Verdah  108 INKGKVFYHFSIKRSDVNPPATTREHQIAFFESHFTELKAGWLSGAAGTSDPLLRWCVGG
Conlig  108 INQGKVFYHLSLMRSDTNPPAQTREHQIGFFESHFTELKAGWLSGAAGVSDPLLRWCVQG
Neucra  108 INKGKVFYHFSLMRKDTNAPALTREHQIAFFESHFTELKSGWQSGAAGTSDPLLRWCIGG
Neutet  108 INKGKVFYHFSLMRKDTNAPALTREHQIAFFESHFTELKSGWQSGASGTSDPLLRWCIGG
Sormac  108 INQGKVFYHFSLMRKDTNAPATTREHQIAFFESHFTELKSGWQSGASGISDPLLRWCIGG
Madmyc  108 INSGKVYYHFSLMRKDVNPPATTREHQIAFFESHFTELKSGWLSGAPGISDTLLRWCVGG
Podans  108 INQGKVYYHFSLMRKDINAPATTREHQIAFFESHFTELKSGWLSGAPGISDTLLRWCVGG
Chaglo  108 IAQGKVYYHFSLMRKDTNAPATTREHQIAFFESHFTELKAGWLSGAPGVSDTLLRWCVGG
Thethe  108 INQGKVYYHFSLMRKNTNPPATTREHQIAFFESHFTELKSGLLSGAPGESDSLLRWCVGG
Thiter  108 INKGKVYYHFSLMRKDINAPATTREHQIAFFESHFTELKSGLLSGASGVSDTSLRWCVSG
Chathe  105 INRGKVYYHFSLMRKDVNAPATTREHQIAFFESHFTELKSGWLSGSPGTEDTKLRWCVSG
Gaetri  111 INKGKVYYHFSIKRSDVNPPATTREHQIAFFESHFTELKSGWLSGAPGTSDPALRWMVNQ
Magory  108 INQGKVFYHFSIKRSDVNAPATTREHQIAFFESHFTELKSGWLSGAPGTSDPALRWMVNQ
Maggri  108 INQGKVFYHFSIKRSDVNPPATTREHQIAFFESHFTELKSGWLSGAPGTSDPALRWMVNQ
Stacha  110 IASGRVFYHFSIKREDVNAPALTREHQIAFFESHFTELKSGWLSGAPGIEDPLLRWQVGG
Stachl  110 IASGRVFYHFSIKREDVNAPALTREHQIAFFESHFTELKSGWLSGAPGIEDPLLRWQVGG
Micbol  107 IASGKVWYHFSIKRSNTNAPAQTREHQIAFFESHFTELKSGLLSGQPGTSDPALRWMVGG
Colfio  104 INSGKVWYHFSISRKDTNAPSIFREHQIAFFESHFTELKSGWISGESATNNTNLQFMVQQ
Colnym  104 INSGKVWYHFSISRKDTNAPSIFREHQIAFFESHFTELKSGWISGESATNNTNLQFMVQQ
Colsal  104 INSGKVWYHFSISRKDVNAPSIFREHQIAFFESHFTELKSGWISGESATSNTNLQFMVQQ
Colgra  104 IASGKVWYHFSISRKDVNAPSVFREHQIAFFESHFTELKSGWISGEQGTSNNNLQFMVQG
Colsub  104 IASGKVWYHFSISRKDANAPSVFREHQIAFFESHFTELKSGWISGEQGTSNNNLQFMVQG
Coltof  104 IASGKVWYHFSISRKDVNAPSVFREHQIAFFESHFTELKSGWISGEQATSNTNLQFMVQG
Colorb  104 IASGKVWYHFSISRKDVNAPSVFREHQIAFFESHFTELKSGWISGEQAASNPNLQFMVQQ
Colglo  104 INSGKVWYHFSIMRKDTNAPSIYREHQIAFFESHFTELKSGWISGESATSNANLQFMVQQ
Phamin  106 INKGKVYYHFSLMRSDKNAPSVNREHQINFFESHFTELKYGWISGESGTSNPNLQWMVSQ
Pesfic  102 INSGKVWYHFSISRSDTNAPSVNREHQICFFESHFTELKYGWISGESGTSDPYLQFMISQ
Psevex  102 INKGKVYYHFSLLRKDTNAPSVNREHQINFFESHFTEMKYGWISGESGTSNPALQFMISQ
Rosnec  106 INSGKVYYHFSMMRKDTNAPSVNREHQICFFESHFVEMKSGWISGEQGTSNPNLQFMVSQ
Daldsp  104 INSGKVFYHFSIMRKDTNAPSKNREHQICFFESHFTELKYGWISGEQGTENPNLQFMVSQ
Hyposp  104 INSGKVFYHFSISRKDTNAPSKNREHQICFFESHFTELKYGWISGEQGTENPNLQFMISQ
Ophpic  104 IASGKVFYHFSIMRKDTNAPSVNREHQICFFESHFTELKSGWISGEQGTSNPNLQWMVSQ
Spobra  106 IASGKVFYHFSVMRKDTNAPSVNREHQICFFESHFTELKYGWISGEQGTSNPNLQWMVSQ
Sposch  106 IASGKVFYHFSIMRKDTNAPSVNREHQICFFESHFTELKYGWISGEQGTSNPNLQWMVSQ
Spoins  113 IATGKVVYHFSIMRKATNAPSINREHQICFFESHFTELKYGWISGEQGTSNPNLQWMVSQ
Lompro  107 INKGKVFYHFSMMRKATNPPSVNKEHQICFFESHFTEMKYGWISGEQGTENPNLQFMVSQ
Sceapi  107 INKGKVFYHFSMMRKAQNAPSVNKEHQICFFESHFTEMKYGWISGEQGTENPNLQFMVSQ
Diaamp  108 INQGKVWYHFSMMTSGTNYPSIYREHQINFFESHFTEMKSGWISGEAATSNPNLQWMVGG
Neodit  105 IAAGKVYYHFSIKRADENAPSLTREHQIAFFESHFTELK----SGLSGTED-NLQWFVGG
Eutlat  107 INEGKVWYHFSILRKDVNPPAETREHQIAFFESHFTELKSGWLSGSSGTDDPLLRWMVGG
Conlus  115 INEGTVWYHFSMMANATDFPSPYREHQICFFESHFTEMKAGWISGEAATSDGLLRWDVQS
Cerpla  105 INKGKVTYHFSMMRSETNAPSIFREHQIAFFESHFCEMKYGWLSGESGESNPNLQFFAAS
Thipun  104 INKGKTVYHFSIMRKDTNAPSVNREHQIAFFESHFCEMKYGWVSGESGTSDTNLQFFANS
```

```
Colchl  168 KTEWSANWDADVWHNVAYEIDFDAGSVGFWHSTGSDALKQVVAPVKASTQSNGADWHVGV
Colhig  168 KTEWSVNWDADVWHNVAYEIDFDAGSVGFWHSTGSEALTQVVAPVTAAASSNGADWHVGV
Colinc  168 KTEWSVNWDADVWHNVAYEIDFDAGSVGFWHSTGSAALTQVVAPVTASASSNGADWHVGV
Colsim  168 KTEWSVNWDADVWHNVAYEIDFDGGSVGFWHSTGAEALTQVVAPVTASASSNGADWHVGV
Veralf  169 QTHWSVNWDADVWHNVAYEIDFSAGTVGFWHSTGASSLTRVNAPVAASTSSNGADWHLGV
Verlon  169 QTHWSVNWDADVWHNVAYEIDFSAGTVGFWHSTGASPLNRVNAPIAASTSINGADWHLGV
Verdah  168 QTHWSVNWDADVWHNVAYEIDFSAGTVGFWHSTGASPLTRVNAPIAASTSSNGADWHLGV
Conlig  168 NTKWSVNWDAGVWHNVAYEIDFSANTVGFWHSTGGDNLVQVVAPQSVSTSSNGADWHVGV
Neucra  168 QTKWSVNWDADVWHNVAYEIDFDANTVGFWHSTGSDALTQVIAPQAAGTSSNGADWHVGV
Neutet  168 QTKWSVNWDADVWHNVAYEIDFDANTVGFWHSTGSDALTQVIAPQAAGTSSNGADWHVGV
Sormac  168 QTKWSVNWDAGVWHNVAYEIDFSANTVGFWHSTGSDDLVRVIAPQSASTSSNGADWHVGV
Madmyc  168 QTQWSTEWEADIWHNVAYEIDFGAGTVGFWHSTGSDPLTQKVAPVSASTSSNGADWHVGV
Podans  168 QTQWSVEWAADVWHNVAYEIDFAAGTVGFWHSTGSDPLTRKVAPVKTSTSSNGADWHVGV
Chaglo  168 QTKWSTEWEANVWHNVAYEIDFSANTVAFWHSTGGDALTQKIAPVSTSTSSNGADWHVGV
Thethe  168 QTQWSTDWEADVWHNVAYEIDFSANTVGFWHSTGSDPLTQKVAPISASTSSNGADWHVGV
Thiter  168 QTQWSTEWVADVWHNVAYEIDFDANTVGFWHSTGSDALVQVVAPVSVSTSSNGADWHVGV
Chathe  165 QTHWSVEWEADVWHNFAYEIDFSAGTVGLWHSENGEPLKRVVDPVRTSASSDGKDWHVGV
Gaetri  171 QTKWSVDWAAGVWHNVAYEIDFGAGTVGFWHSTGADPLTRTVAPVSVSTSSNGADWHLGV
Magory  168 QTKWSVDWAADVWHNVAYEIDFAANTVGFWHSTGASPLQRIVAPVSVSTSSNGADWHVGV
Maggri  168 QTKWSVDWAADVWHNVAYEIDFSANTVGFWHSTGGDPLQRIVAPVSVSTSSNGADWHVGV
Stacha  170 QTRWSVNWEAGVWHNVAYEIDFTAGTVGFWHSTGSSNLQRIVAPVSASTSSNGADWHLGV
Stachl  170 QTRWSVNWEAGVWHNVAYEIDFTARTVGFWHSTGSSNLQRIVAPVSASTSSNGADWHLGV
Micbol  167 VTKWSVNWDADVWHNCAYEIDFSANKVGFWHSTGSGDLVQTVAPISASTSSNGADWHLGV
Colfio  164 KSLWKTEWKPDVWHNVAYEINFSSGSVGFWHSEGSAPLQQVVAPVSASTSSNGADWHLGV
Colnym  164 KSLWKTEWKPDVWHNVAYEINFSSGSVGFWHSEGSAPLQQVVAPVSASTSSNGADWHLGV
Colsal  164 KSLWKTEWKPDVWHNVAYEINFSSGSVGFWHSEGSAPLQQVVAPVSASTSSNGADWHLGV
Colgra  164 KSLWKTEWKPDVWHNVAYEINFSSGSVGFWHSEGSAPLQQVVAPVSASTSSNGQDWHLGV
Colsub  164 KSLWKTEWKPDVWHNVAYEINFSGGSVGFWHSEGNAPLQQVVAPVSAPTSSNGQDWHLGV
Coltof  164 KSLWKTEWKPDVWHNVAYEINFSSGSVGFWHSEGGAPLQQVVAPVSASTSSNGQDWHLGV
Colorb  164 KSLWKTEWKPDVWHNVAYEINFSSGSVGFWHSEGSAPLQQVVAPVSASTSSNGQDWHLGV
Colglo  164 KSLWKAEWKAGVWHNVAYEINFSSGSVGFWHSEGSAPLTQVVAPVSASTSSNGADWHLGV
Phamin  166 KSYWKTEWTPGVWHNVAYEIDFSANKIGFWHSTGGDPLTQVVAPVSGSTSSNGADWHLGV
Pesfic  162 NSKWKTEWKAGVWHNVAFEIDFSKNTVGFWHSEGGAPLTQIVAPQSASTSSNGADWHLGV
Psevex  162 NSKWKTEWLPDVWHNVAYEIDFSANTVGFWHSEGSAPLTQVVKPASASTSSNGADWHLGV
Rosnec  166 NSKWKTEWKANVWHNIAYEIDFSRNTVGLWHSEGSSPLAQAVAPVSASTSSNGADWHLGV
Daldsp  164 NSKWKTEWKPNVWHNVAFEIDFSSGTVGFWHSEGADALKQLVAPVKTSTSSNGADWHLGV
Hyposp  164 NSKWKTEWKPNVWHNVAFEIDFGAGTVGFWHSEGADPLTQVVAPAKTSTSSNGADWHLGV
Ophpic  164 QSQWKTEWKPDVWHNVAYEIDFS-GTVGFWHSEGGDPLKQVVAPVSVSASSNGADWHLGV
Spobra  166 KSQWKTEWKPDVWHNVAYEIDFS-GKVGFWHSEGGDPLKQVVAPVSVSASSNGADWHLGV
Sposch  166 KSQWKTEWKPDVWHNVAYEIDFA-GKVGFWHSEGGDPLKQVVAPVSVSASSNGADWHLGV
Spoins  173 KSQWKTEWKPDVWHNVAYEIDFS-GHVGFWHSEGGDPLTQVVAPVAVSASSNGADWHLGV
Lompro  167 KGLWKTEWVPDVWHNIAYEIDFGANTVGFWHSEGADPLEQVVAPVAASTSSNGADWHLGV
Sceapi  167 KGLWKTEWAPDVWHNIAYEIDFGANKVGFWHSEGDEPLTQKVAPVAASTSSNGADWHLGV
Diaamp  168 VSKWSTNFTAGVWHNVAYEIDFSANTVGFWHSTGSDPLELTVSPVSASTSSNGADWHLGV
Neodit  160 TSKWEAEWAAGVWHNIAYEIDFDASTVGLWHSTGADALTQVVKPVTASTSSNGADWHVGV
Eutlat  167 TTEWSTEFLPEVWHNVAYEIDFSANTVGFWYSTGAEDLKQTIEPVSASTSSNGQDWHLGV
Conlus  175 VTQWSTEFEAGVWYNIAYEINFTAGTVGFWYSTGGNDLVQTVAPMAASASSNGQDWHLGV
Cerpla  165 KSQWKAEWAAGVWHNIAYEIDFDGNTVAFWHSTGSDPLTKVVDAVSVTATSDGKDWHLGV
Thipun  164 KSQWKATWEPNVWHNIAYEIDFSGGTVAFWHSTGSDALTKVVDAVGVSAQSNGADWHVGV
```

```
Colchl  228 LELPRDGYPDETEDFYFSGVYIEDGELTTAVGSGSS
Colhig  228 LELPRDGYPDETEDFYFSGVYIEDGELTTSVGSGAA
Colinc  228 LELPRDGYPDETEDFYFSGVYIENGELTTSVGSGSA
Colsim  228 LELPRDGYADETEDFYFSGVYIEDGEITKAV--GS-
Veralf  229 LELPRSGYPDAPEDIYFSGVYIEDGTITTSVAGPGS
Verlon  229 LELPRSGYPDAPEDIYFSGVYVEDGTITTSVAGPGS
Verdah  228 LELPRSGYPDAPEDIYFSGVYIEDGTITTSVAGPGS
Conlig  228 LELPRDGYPDSTEDYYWSGVYIESGSITTSVSGPGS
Neucra  228 LELPRDGYADATEDFYFSGVYIESGSITTSVAGPGS
Neutet  228 LELPRDGYADATEDFYFSGVYIESGSITTSVAGPGT
Sormac  228 LELPRDGYPDTTEDYFWSGVYIESGSITTSVSGPGS
Madmyc  228 LELPRSGYPDSTEDFYWSGVYIESGDLTTSVAGPGA
Podans  228 LELPRSGYPDSNEDFYWSGVYIESGSLTTSVAGPGQ
Chaglo  228 LELPRSGYSDSNEDYYWSGVYIESGSLTTNVAGPGA
Thethe  228 LELPRSGYPDTTEDYYWSGVYIESGSITTNIAGPGA
Thiter  228 LELPRSGYPDTTEDFYFSGVYIESGSLTTSVSGPGS
Chathe  225 LELPRSGYNDFDEDFYFSGVYIENGEITTSVSGPGE
Gaetri  231 LELPRSGYPDAVEDFYFSGVYIETGSITTAIGTGGG
Magory  228 LELPRSGYPDAVEDFYFSGVYIETGTITTAIGDGSG
Maggri  228 LELPRSGYPDAVEDFYFSGVYIETGTITTAIGDGSG
Stacha  230 LELPRSGYADENEDFFFSGVYIENGEITTSVSGPGT
Stachl  230 LELPRSGYADENEDFFFSGVYIENGEITTSVSGPGT
Micbol  227 LELPRSGYSDSNEDFYFSGVYIESGSITKSVTGPK-
Colfio  224 LELPRDGYADTNEDFYFSGVYVEDTAITTSVTGPAA
Colnym  224 LELPRDGYADTNEDFYFSGVYVEDTAITTSVTGPGE
Colsal  224 LELPRDGYADTNEDFYFSGVYVEDTAITTSVTGPGE
Colgra  224 LELPRSGYSDTNEDFYFSGVYVEDGAITTSVTGPGR
Colsub  224 LELPRTGYSDTNEDFYFSGVYVEDGAITTSVTGPAA
Coltof  224 LELPRNGYADTDEDFYFSGVYVEDGAITTSVTGPAA
Colorb  224 LELPRNGYPDTTEDFYFSGVYVEDGAITTSVTGPAA
Colglo  224 LELPRDGYPDSNEDFYFSGVYVEDTAITTSVTGPGE
Phamin  226 LELPRSGYSDTTEDFYFSGVYIESGSITTAIGGPK-
Pesfic  222 LELPRSGYSDANEDFYFSGVYIESGSMTTSVNGPAA
Psevex  222 LELPRSGYSDSNEDFYFSGVYIESGTLTTAVSGATA
Rosnec  226 LELPRSGYPDTTEDFYFSGVYVESGTITTAIGGPIA
Daldsp  224 LELPRSGYSDGVEDFYFSGVYVESGPITTSIGGPTA
Hyposp  224 LELPRSGYPDSVEDFYFSGVYIESGPITTAIGGPA-
Ophpic  223 LELPRSSYSDTNEDFYFSGVYIESGPITTTIGGPAA
Spobra  225 LELPRSGYNDANEDFYFSGVYIESGPITTTIGGPAA
Sposch  225 LELPRSGYNDANEDFYFSGVYIESGPITTTIGGPAA
Spoins  232 LELPRSGYSDGVEDYYFSGVYIESGPVTTAIGGPPA
Lompro  227 LELPRDGYPHENEDFYFSGVYIEDGTITTDVAGPA-
Sceapi  227 LELPRDGYPHQNEDFYFSGVYIEDGTITTDVSGPA-
Diaamp  228 LELPRSGYSDSNEDFYFSGVYVESGSLTTTIGSGGE
Neodit  220 LELA-NGDADETEDFYFSGVYIEDGELTTAISSSKS
Eutlat  227 LELPRDGYTDEVEDLYFSGVYVESGSITTTISGPAS
Conlus  235 LELPRGGYTDTDENLYFSGVYIESGSITTSVSGPGG
Cerpla  225 LELQRTGYEDADEDFYFSSVYIEDGEVTTGFPAGGS
Thipun  224 LELPRSGYTDSNEDFYFAGVYIEDGDVTTTMGGSSS
```

__Sordariomycetes__

**Hypocreomycetidae - Glomerellales**

Colchl = OLN87414.1 hypothetical protein CCHL11_09568 [Colletotrichum chlorophyti] SAPAATASAKPACRRRRRRSNKAKRN

Colfio = EXF79761.1 hypothetical protein CFIO01_10221 [Colletotrichum fioriniae PJ7]

Colglo = EQB46944.1 hypothetical protein CGLO_13971 [Colletotrichum gloeosporioides Cg-14]

Colgra = XP_008089016.1 hypothetical protein GLRG_00140 [Colletotrichum graminicola M1.001]

Colhig = CCF34200.1 hypothetical protein CH063_06244 [Colletotrichum higginsianum]

Colinc = KZL80950.1 hypothetical protein CI238_10924 [Colletotrichum incanum]

Colnym = KXH48861.1 hypothetical protein CNYM01_04877 [Colletotrichum nymphaeae SA-01]

Colorb = TDZ18371.1 hypothetical protein Cob_v008530 [Colletotrichum orbiculare MAFF 240422]

Colsim = KXH30104.1 hypothetical protein CSIM01_00818 [Colletotrichum simmondsii]

Colsal = KXH61778.1 hypothetical protein CSAL01_07891 [Colletotrichum salicis]

Colsub = KDN72294.1 hypothetical protein CSUB01_06221 [Colletotrichum sublineola]

Coltof = KZL74381.1 carbohydrate-binding module family 1 protein [Colletotrichum tofieldiae] corrected to remove spurious N-terminal sequence
Veralf = XP_003008315.1 conserved hypothetical protein [Verticillium alfalfae VaMs.102]
Verdah = KAF3351428.1 Putative transcriptional regulatory protein [Verticillium dahliae VDG2]
Verlon = KAG7128176.1 hypothetical protein HYQ45_012077 [Verticillium longisporum]
**Hypocreomycetidae - Hypocreales - Clavicipitaceae - no orthologues found**
**Hypocreomycetidae - Hypocreales - Cordycipitaceae - no orthologues found**
**Hypocreomycetidae - Hypocreales - Hypocreaceae - no orthologues found**
**Hypocreomycetidae - Hypocreales - Nectriaceae (but no orthologues found in Fusarium)**
Neodit = KPM42463.1 hypothetical protein AK830_g4089 [Neonectria ditissima]
**Hypocreomycetidae - Hypocreales - Ophiocordycipitaceae - no orthologues found**
**Hypocreomycetidae - Hypocreales - Stachybotrys**
Stacha = KEY67053.1 hypothetical protein S7711_10884 [Stachybotrys chartarum IBT 7711]
Stachl - KFA65100.1 hypothetical protein S40285_03114 [Stachybotrys chlorohalonata IBT 40285]
**Hypocreomycetidae - Microascales**
Cerpla = KKF94894.1 hypothetical protein CFO_g2760 [Ceratocystis platani]
Thipun = KKA29646.1 hypothetical protein TD95_000695 [Thielaviopsis punctulata]
Lompro = PKS10019.1 hypothetical protein jhhlp_004644 [Lomentospora prolificans]
Sceapi = XP_016641373.1 hypothetical protein SAPIO_CDS7745 [Scedosporium apiospermum]
**Sordariomycetidae - Coniochaetales**
Conlig = OIW27922.1 carbohydrate-binding module family 1 protein [Coniochaeta ligniaria NRRL 30616]
**Sordariomycetidae - Togniniales**
Phamin = XP_007910843.1 putative carbohydrate-binding module family 1 protein [Phaeoacremonium minimum UCRPA7]
**Sordariomycetidae - Diaporthales**
Conlus = PSR77661.1 hypothetical protein BD289DRAFT_377636 [Coniella lustricola]
Diaamp = KKY33924.1 putative carbohydrate-binding module family 1 protein [Diaporthe ampelina]
**Sordariomycetidae - Magnaporthales**
Gaetri = XP_009223752.1 hypothetical protein GGTG_07663 [Gaeumannomyces tritici R3-111a-1]
Magory = >QBZ57551.1 hypothetical protein PoMZ_02480 [Pyricularia oryzae] (syn. Magnaporthe oryzae)
Maggri = XP_030981300.1 uncharacterized protein PgNI_07823 [Pyricularia grisea] (syn. Magnaporthe grisea)
**Sordariomycetidae - Ophiostomataceae**
Ophpic = EPE07306.1 carbohydrate-binding module family 1 protein [Ophiostoma piceae UAMH 11346]
Spobra = XP_040620187.1 uncharacterized protein SPBR_03181 [Sporothrix brasiliensis 5110]
Spoins = OAA53838.1 hypothetical protein SPI_09283 [Sporothrix insectorum RCEF 264]
Sposch = XP_016590501.1 hypothetical protein SPSK_07902 [Sporothrix schenckii 1099-18]
**Sordariomycetidae - Sordariales - Sordariales incertae sedis**
Madmyc = KXX78310.1 Feruloyl esterase B [Madurella mycetomatis]
**Sordariomycetidae - Sordariales - Chaetomiaceae**
Chaglo = XP_001228503.1 uncharacterized protein CHGG_10576 [Chaetomium globosum CBS 148.51]
Chathe = XP_006690661.1 hypothetical protein CTHT_0001080 [Chaetomium thermophilum var. thermophilum DSM 1495]
Podans = AFQ89876.1 beta-glucanase [Podospora anserina]
Thethe = XP_003667321.1 carbohydrate-binding module family 1 protein [Thermothelomyces thermophilus ATCC 42464]
Thiter = SPQ27187.1 1b90b30c-5168-4e91-890b-de3dbdb1dae1 [Thermothielavioides terrestris] (syn. Thielavia terrestris)
**Sordariomycetidae - Sordariales - Sordariaceae**
Neucra = XP_958348.1 hypothetical protein NCU09764 [Neurospora crassa OR74A]
Neutet = XP_009851721.1 hypothetical protein NEUTE1DRAFT_64374 [Neurospora tetrasperma FGSC 2508]
Sormac = XP_003344812.1 uncharacterized protein SMAC_09184 [Sordaria macrospora k-hell]
**Xylariomycetidae - Xylariales**
Eutlat = EMR71091.1 putative carbohydrate-binding module family 1 protein [Eutypa lata UCREL1]
Daldsp = OTB20440.1 hypothetical protein K445DRAFT_312903 [Daldinia sp. EC12]
Hyposp = OTA66562.1 glycoside hydrolase family 131 protein [Hypoxylon sp. EC38]
Micbol = KXJ89759.1 hypothetical protein Micbo1qcDRAFT_212247 [Microdochium bolleyi]
Pesfic = XP_007838550.1 hypothetical protein PFICI_11778 [Pestalotiopsis fici W106-1]
Psevex = XP_040719926.1 uncharacterized protein BCR38DRAFT_96685 [Pseudomassariella vexata]
Rosnec = GAP89226.2 putative carbohydrate-binding module family 1 protein [Rosellinia necatrix]

**Supplementary Figure S5. PaGluc131A alignment.** Multiple protein-sequence alignment of the N-terminal

glycosyl hydrolase domains of PaGluc131A orthologues in the PaGluc131A/CcGH131A clade of Sordariomycete GH131 (glycosyl-hydrolase 131) sequences. All sequences shown were the highest blastP matches to PaGluc131A (= Podans highlighted in green in the alignment) obtained for each genome-sequenced species represented. Sequences were aligned using ClustalW at https://www.ebi.ac.uk/Tools/msa/mafft/. Amino-acid identities were highlighted in back and similarities in grey using BoxShade at https://embnet.vital-it.ch/software/BOX_form.html. Residues conserved with the catalytic residues of PaGluc131A and CcGH131A have been highlighted manually in red. C-terminal extensions, including those containing CBM1 (carbohydrate binding module 1) domains, were removed manually for the purposes of alignment.
