# Supplementary material for: Transcriptome Analysis of Fusarium–Tomato Interaction Based on an Updated Genome Annotation of Fusarium oxysporum f. sp. lycopersici Identifies Novel Effector Candidates That Suppress or Induce Cell Death in Nicotiana benthamiana
Source: J Fungi (Basel). 2022 Jun 26;8(7):672. doi: 10.3390/jof8070672 (PMC9316272; doi:10.3390/jof8070672)
Supplement: Supplementary file 1 [file jof-08-00672-s001.zip › Supplementary Materials/Supplementary Figures/Supplementary Figure S6.pdf]

Signal peptide

```
Fol-EC14   1 MRTA-AF--I-TLLASVVSAT----PFGQR-----SQVLARASETCPVVFDGRVPANASL
Fuspro1    1 MRTA-AF--V-TLLASVVSAS----PFGQR-----SQVLARASETCPVVFDGRVPANASL
Fusman1    1 MRTA-AF--I-ALLASVVSAS----PFGQR-----SQVLARASETCPVVFDGRVPANASL
Fusfuj1    1 MRTA-AF--I-TLLASVVSAT----PFGQR-----SQVLARASETCPVVFDGRVPANASL
Fusnyg1    1 MRTA-AV--I-TLLASVVSAT----PFGQR-----SQVLARASETCPVIFDGRVPANASL
Fusver1    1 MRTA-SV--I-TLLASVVSAT----PFGQR-----SQVLPRASETCPVVFDGRVPANASL
Fuscul1    1 MRAA-AI--I-ALFASVAFAA----PADSR-------SCSKAKNTCPVVFDGRVPANASL
Fusgra1    1 MRAA-AI--I-ALFASVAFAA----PADSR-------SCSKAKNTCPVVFDGRVPANASL
Fuspse1    1 MRAA-AV--I-ALFASVAFAA----PADSR-------SCSKAKNTCPVVFDGRVPANASL
Fuslan1    1 MRAA-AI--I-TLLASVASAA----PADNR-------SCSKAKNTCPVVFDGRVPANASL
Fuspoa1    1 MRAA-AI--I-TLLASVASAA----PADDR-------SCSKAKNTCPVVFDGRVPANATL
Fusven1    1 MRAA-AI--I-TLLASVASAA----PADDR-------SCSKAKNACPVVFDGRVPANATL
Neodit1    1 MHRS-AV--F-ALLASAASAA----PF-HG-----FHAFTNKSSSCPVIFDGRVPANASL
Fussol1    1 MRAA-AF--F-ALVSSVVSAT----PLGHR-----FHTYSKASNSCAVVFDGRVPANASL
Colchl1    1 MPLQ-VI--I-LLLTTVALAT----TKP---------CTPPPLTKCPVILDGRIPSFAIS
Colfio1    1 MRSSFLI-PL-LLAIPSSLAS----PSP----------CLTATIQCPLVFDGRVPVTATP
Colnym1    1 MRSPFLI-PI-LLAIPSALGS----PSP----------CTAAAIQCPLVFDGRVPATATP
Colsim1    1 MRSPFLI-PF-LLAIPSALAS----PSP----------CLTATIQCPLVFDGRAPATATP
Colglo1    1 MHP------L-TLLTLATPAL----SVTL---------TCRSL-IRCPLIFDGRVPSTAIP
Colgra1    1 MFSR-AA-LL-LLLTPATLAA----ATP---------NCHAAAVRCPIVFDGRVPAVATP
Colinc1    1 MFSH-TA--L-LLLAPAALAA----STL---------GCRAASLKCPIVFDGRVPATATP
Coltof1    1 MLPH-TA--L-LLLAPAAFTA----ATL---------NCRAASIKCPIVFDGRVPATATP
Colhig1    1 MIPP-AA--L-LLLALAILAA----SSPS--------NCGAASIRCPIIFDGRVPAAAVP
Colorb1    1 MRLF----LLSILFLSLTLAA--------------SLRCSAPSIQCPLIFDGRVPSTAIP
Veralf1    1 MLPS-VLTVL-SALVASSIAK----PVT---------KCRQAAVTCPIVFDGRVPQTATV
Verdah1    1 MLPS-VLTVL-SALVASSLAK----PVT---------KCRQAAVTCPIVFDGRVPQTAKV
Verlon1    1 MLPS-VLTVL-SALVASSFAK----PVT---------KCRQAAVTCPIVFDGRVPQTATV
Lompro1    1 MFRS-----L-FFLALAQAAF----ALP------------AAEPSCPVVFDGRVPADAEP
Sceapi1    1 MFRS-----L-LLLALTQGIF----ALP------------AAEPSCPIVFDGRVPKDASL
Chathe1    1 MHR--SL-LL-TLLTTAIAVLARP-PSRHPHS--PSAAAAPGTIKCPIIFDGRIKTTTQL
Madmyc1    1 MHH--AI-LL-SLL-TAGTAVTAR-PSFSQEP--PINQHVATAIQCPIVFDGRVGATTEL
Thiter1    1 MHR--AT-LV-GLLAAAATTVTGA-PSCSG-----VHAAAPGTVKCPLVFDGRVKATAAL
Thethe1    1 MHST-TT-LL-SLLSAAAAANGASLPRDSV-------CGAAAEIQCPIVFDGRVSADAQP
Conlig1    1 MVHQ-TL-LL-ALLSALTVSAR---P-----------SKCAAEPKCPIVLDGRVKTTLVP
Daldsp1    1 MISS-AL-FTGFLLASSASAR----PT----------KRGTGEIKCPIILNGFVPADLTP
Hyposp1    1 MISS-AL-LTGLLLTSSASAH----PT----------KRGTGEIKCPVVLSGFVPVDKTP
Rosnec1    1 MVSS-TL-LLGGILAAATLSFALP-PSPPE-------RRGAGAIKCPVVLNGLVGVSLTP
Phamin1    1 MVSY-TL-VF-AALAAAVSGA----P-----------CASNATIKCPIVFDGRIAASAAL
Spobra1    1 MKAFSSL-LF-AGVAAAV-------PTGQA----ADADTPAATVKCPIVFDGRVPSTLAL
Sposch1    1 MKAFSSL-LF-AGVAAAV-------PTGQA----ADADAPAATVKCPIVFDGRVPSTLVL
Spoins1    1 MHALASL-LLTAGVAVAVPASSSS-SSAAASTP-TAAAAAAATVKCPIVFDGRVPPSLKL
Podans1    1 MHP--SL-LL-GLLAGTALAGTIRRPGRPR------PQAPTAETLCPIVFDGRPDSSLEP
Neucra1    1 MLT--PT-IL-TLLFTLATTALAV-PQPH-----------AALIKCPVILDGRIKIHTQL
Neutet1    1 MLT--PT-IL-TLLFTLATTALAV-PHPH-----------AALIKCPLIFDGRIKTRTQL
Sormac1    1 MHA--ST-IF-GFLAAAATAVLAV-PHPHK---------EKAAVKCPLIFDGRIKSRTLP
Pesfic1    1 MFS--QI-VMSSLLATLATAR----PSP------------RAAASCPVVLSGRVPASAQL
Psevex1    1 MFSV-AL-VF-GLTATAASAG----PL----------LTPRIDITCPIVLEGRVASSTAL
Eutlat1    1 MVSS-TI-LV-SLFASLAVARR---PGRRQ---------GSGTITCPITLDGRVPVDTEL
Gaetri1    1 MTLA-RH-LANALLAVAVAVA-------------------GAAEVKCPIVFDGRVPASLAL
Magory1    1 MPLA-AL-IPALLLAAAAAAAAT-PLSPR------AADPAGPIRCPIVFDGRVPATLAA
Maggri1    1 MPLA-PL-FSVLLLAAAASAT----PLAVR------AADPTGTIQCPIVFDGRVPTTLVS
Conlus1    1 MLFT-SP-IL-ALSALVAIAT----ASPRICQTHASKTTNSSTIKCPIVLDGRVPANASL
Diaamp1    1 MFSA-SL-LL-ALGTTAVAAI----PYETR-------SPSSGVVQCPVILEGRIPASSVL
Diahel1    1 MFSV-SL-LL-GLGATIAAAI----PSGPR-------SLTSGSVKCPVVFEGRVPTSTTL
Micbol1    1 MALK-AF-FTTSLVALASLASALP-PVPGR------PGATATKISCPVVFSGQIPIAAKL
```

```
Fol-EC14  48 TDFDTANGGGWNPYNPGFVKGNNISWSEILQLP---------K-TKT--------K-SRF
Fuspro1   48 TDFDTANGGGWNPYNPGFVKGNNISWSEILQLP---------K-TKT--------K-SRF
Fusman1   48 TDFDTANGGGWNPYNPGFVKGNNISWSEILQLP---------K-TKT--------K-SRF
Fusfuj1   48 TDFDTANGGGWNPYNPGFVKGNNISWSEILQLP---------K-TKT--------K-SRF
Fusnyg1   48 TDFDTANGGGWNPYNPGFVKGNNISWSEILKLP---------K-TKT--------K-SRF
Fusver1   48 TDFDTANGGGWNPYNPGFVKGNNISWSEILKLP---------K-TKT--------K-SRF
Fuscul1   46 ADFDKENGGGWNPYNPGFVKGNNISWSEIIKLP---------Q-TTP----------SRF
Fusgra1   46 TDFDKENGGGWNPYNPGFVKGNNISWSEIIKLP---------Q-TTP----------SRF
Fuspse1   46 TDFDKENGGGWNPYNPGFVKGNNISWSEIIKLP---------Q-TTP----------SRF
Fuslan1   46 SDFDKENGGGWNPYNPGYVKGNNISWSEIVKLP---------Q-TTP----------SRF
Fuspoa1   46 ADFDKENGGGWNPYNPGYVKGNNISWSEIIKLP---------Q-TTP----------SRF
Fusven1   46 ADFDKENGGGWNPYNPGFVKGNNISWSEIVKLP---------Q-TTP----------SRF
Neodit1   47 ADFDTARGGGWNPFNPGYVKGNNLSWSDILLLP---------N-HVP--------A-SRF
Fussol1   48 TDFDTENGGGWNPFNPGYVKGNNISWSEILLLP---------K-NTP--------R-SRF
Colchl1   44 IDFNTAQGGGWSPFNPDYVKGSSLAWSDILLLP---------D-AVP--------P-SRF
Colfio1   45 RDFDTSLGGGWNPYNPDYVKGTNLSWSDIILLP---------G-PAPVPTRPGSRS-SRF
Colnym1   45 QDFDTALGGGWNPYNPDYVKGASLSWSDIILLP---------E-SAPVSTRPGSRS-SRF
Colsim1   45 QDFDTALGGGWNPYNPDYVKGTNLSWSDIILLP---------E-SAPVSTRPGSRS-SRF
Colglo1   41 TDFDTPNGGGWNPFNPSYVKGNTLLWSDIVLLP-----------SGP--------T-SRF
Colgra1   45 QDFDTASGGGWNPYNPGYVKGEGLLWSDIILLPLPSPQEEEEE-AAP--------P-SRF
Colinc1   44 QDFDSASGGGWNPYNPGYVKGEGLLWSDIILLP---------Q-VAP--------P-SRF
Coltof1   44 QDFDSASGGGWNPYNPGYVKGEGLLWSDIILLP---------Q-VAP--------P-SRF
Colhig1   45 GDFDSASGGGWNPYNPDYVKGKGLLWSDIILLP---------R-AGP--------P-SRF
Colorb1   43 TDFDSSTT--WNPYNPSYVKGASLFWSEILLFP-----------PTP--------P-SRF
Veralf1   46 QDFDTAGGGGFNPFNPNFVKGNNLTWSEILLLP---------T-ITP--------A-SRF
Verdah1   46 QDFDTAGGGGFNPFNPNFVKGNNLTWSEILLLP---------T-ITP--------A-SRF
Verlon1   46 QDFDTAGGGGFNPFNPNFVKGNNLTWSEILLLP---------T-ITP--------A-SRF
Lompro1   39 TDFDANNGGGWMPFNSGYVKGETLKWSEILQFP---------E-VAE--------A-SRF
Sceapi1   39 TDFDANNGGGWMPFNPGYVKGETLKWSEILKLP---------E-VAE--------A-SRF
Chathe1   54 TDFDSYST---SPFNPDYVKGQTLKWSDIIKFP---------S--VS--------P-SRF
Madmyc1   53 TDFDSYGT---SIFNPDYVKGQNLKWSQIIKFP---------E--VA--------N-SRF
Thiter1   51 TDFDSAST---SPFNPDYVKGNNLKWSQIIKFP---------Q--TS--------N-ARF
Thethe1   51 TDFDSAST---SIFNPDYVKGNNLKWSDILQFP---------D--AG--------T-THF
Conlig1   44 TDFDSYAT---SPFNPDYVKGNNLKWSDILQFP---------T-DVG--------T-SRF
Daldsp1   45 TDFDSYAT---SPFNPDYIRGSE-KFSEILQFP---------E--VE--------S-SRF
Hyposp1   45 TDFDSYAT---SPFNPDYIRGSE-KFSQILLFP---------D--AG--------S-SRF
Rosnec1   51 ADFDSYAT---SPFNPDYVRGGE-KFSQTLRFP---------A-VAA--------S-SRF
Phamin1   43 TDLDSSST---SPFNPDYVKGNSLKWSNIILFP---------S--VP--------N-SRF
Spobra1   48 TDFDSYSKG--SPFNAEYVKGNDLKWSEILLLP---------S--VP--------T-SRF
Sposch1   48 TDFDSYSKG--SPFNAEYVKGNDLKWSEILLLP---------S--VP--------T-SRF
Spoins1   58 ADFDASGSG--SPFNPGYVKGNDLKWSDILLLP---------S--VA--------APSRF
Podans1   51 LDFDDWNT---SPFNPDYVKGAGLPWSSILQFP---------D-ISP--------P-ARF
Neucra1   45 TDFDSSAT---SLFNPDSVKGSSLKWSQIIQFP---------EGDKG----------SRF
Neutet1   45 TDFDSSAT---SLFNPDNVKGSSLKWSQIIQFP---------EGDKG----------SRF
Sormac1   47 TDFDSSST---SLFNPDNVKGSSLKWSQIVKFP---------K-EKG----------SRF
Pesfic1   42 TDFDSSAT---SLFNPDYVRASAIPWSQILLFP---------N--VT--------N-SRF
Psevex1   44 SDFDSYST---SIFNPDYVRGSQ-PWSQILVFS---------N--TT--------N-SRF
Eutlat1   46 ADFDSYAT---SPFNPDYIRGDE-SFSETLLFP---------D--VP--------N-SRF
Gaetri1   41 TDFDSPGRG--SPFGTGYVKGEGLLWSDILKFP---------TAAAG--------P-PRF
Magory1   52 TDLDDAAR---SPFNTGYVKGEGLKWSDIIEFP---------A-GAG--------Q-PRF
Maggri1   49 TDLDDATR---SPFNTGYVKGEGLKWSDIIKFP---------T-EVG--------Q-PRF
Conlus1   54 TSFDTAN----GLFNPDYVKGNNLSWSDILLFP---------T-VAT--------N-SRF
Diaamp1   47 TDFDVEN----DLFNPGYTKGNNLSWSDILLFP---------D--VA--------N-SRF
Diahel1   47 KEFDVEN----ELFNPDYVKGNNLTWSNVVRFP---------D--VP--------N-SRF
Micbol1   52 TDFDAAAT---SLFNPNNVKGND-TWSQIIKFP-------AAAGAAP---GNAACGLARF
```

```
Fol-EC14   89 DI------EAGTIPLEVTISDKSIFMKQLGFRRAGLQFNKDSNEGSPGSKGVKTLHFSIM
Fuspro1    89 DT------EAGTIPLEVTISDKSIFMKQLGFRRAGLQFNKDSNEGSPGSKGVKTLHFSIM
Fusman1    89 DT------EAGTIPLEVTISDKSIFMKQLGFRRAGLQFNKDSNEGSPGSKGVKTLHFSIM
Fusfuj1    89 DT------EAGTIPLEVTISDKSIFMKQLGFRRAGLQFNKDSNEGSPGSKGVKTLHFSIM
Fusnyg1    89 DT------EAGTIPLEVTISDKSIFMKQLGFRRAGLQFNKDSNEGSPGSKGVKTLHFSIQ
Fusver1    89 DT------EAGTIPLEVTISDKSIFMKQLGFRRAGLQFNKDSNEGSPGSKGVKTLHFSIQ
Fuscul1    86 DV------SGKTLPLEVTISDKSIFMKQLGFRRAGLQFSKDKNEGSPGSEGIKTLHFSLM
Fusgra1    86 DV------SGKTLPLEVTISDKSIFMKQLGFRRAGLQFSKDKNEGSPGSEGIKTLHFSLM
Fuspse1    86 DV------SGKTLPLEVTISDKSIFMKQLGFRRAGLQFSKDKNEGSPGSEGIKTLHFSLM
Fuslan1    86 DV------SGKTLPLEVTISDESIFMKQVGFRRAGLQFSKDKNEDSPGSEGIKTLHFSLM
Fuspoa1    86 DA------TGKTLPLEVTISDESIFMKQVGFRRAGLQFSKDKNEGSPGSEGIKTLHFSLI
Fusven1    86 DA------SGKTLPLEVTISDESIFMKQFGFRRAGLQFSKDKNEGSPGSEGIKTLHFSLM
Neodit1    88 DA------AAAAVPVEVTIADESIFMKQNGFRRAGLQFNEDANEDSPASEGVVTLHFSLL
Fussol1    89 DA------VSRTVPLKVTISDESIFMQQHGFRRAGLQFAKDDNEKSPASEGVKTLHFSIL
Colchl1    85 DR------AQNQRPLEVTISDESIFMSQRGFRRAGLLFGADTNTGSPASTGVKTLHFSVR
Colfio1    94 DA------AEKRRSLEVTISDASIFMNQQGLRRAGLLFSGDANEGSPAGEGVKTLHFSVR
Colnym1    94 DA------AEKKRALEVTISDASIFMNQRGFRRAGLLFAGDTNEGSPAGEGVKTLHFSVR
Colsim1    94 DA------AEKKRALEVTLSDASIFMNQRGFRRAGLLFAGDTNEGSPAGEGVKTLHFSVR
Colglo1    81 DA------AAGSRAVEVTISDESIFMSQRGFRRAGLQFAADANTGSPAGEGAKTLHFSVR
Colgra1    95 DA------GGGTRPLEVTVSDRSIFMNQRGFRRAGLQFAADANVGSPAAAGVKTLHFSVR
Colinc1    85 DS------REKKRPLEVTISDKSIFMSQRGFRRAGLQFAKDTNVGSPAGTGAKTLHFSVR
Coltof1    85 DA------REEKRPLEVTISDKSIFMDQRGFRRAGLQFAMDANVGSPAGTGAKTLHFSVR
Colhig1    86 DA-----GEERRRPLEVTISDASVFMDQRGFRRAGLLFAGDANVGGPAGAGVKTLHFSVR
Colorb1    81 DT------LPSTRAIEVTVSDDSIFMNQRGFRRAGLQFLADTNTGSPAAKGVVTLHFSVA
Veralf1    87 DA------AKNTRPVEVTISDESIFMTQLGFRRAELQFANDTGSGSPGSVGVKTLHFSVQ
Verdah1    87 DA------AKNTRPVEVTISDESIFMTQLGFRRAELQFANDTGSGSPGSVGVKTLHFSVQ
Verlon1    87 DA------AKNTRPVEVTISDASIFMTQLGFRRAELQFANDTGSGSPGSVGVKTLHFSVQ
Lompro1    80 DA------DAGTIPVEVTLSDKSIFMTQNGFRRAGLQFLKDTNEGSPATTGKKTIHFSLR
Sceapi1    80 DS------DSGTVPLEVTLSDKSIFMTQNGFRRAGLQFLKDSNEGSPASKGKKTIHFSLR
Chathe1    91 DD------PDQHKPIEVTISDASIFMNQRGFRRAGLQFQGDSNTGSAGSKGVKTIHFSVR
Madmyc1    90 DN-------ETHKPFEVTISDASIFQSQRGFRRAGLQFQGDSNNGSPGSSGVKTIHFSVR
Thiter1    88 DN-------NSTKAIEVTISDASIFQSQQGFRRAGLQFQGDTNNGSPGTTGVKTIHFSIK
Thethe1    88 DN-------ETHKPFEVTLSDESIFQSQQGFRRAGLQFNGDTNNGSPGSQGVKTIHFSVK
Conlig1    82 DN-------ASYKPFEVTISDASIFQQQNGFRRAGLQIQGDTNTGSPANTGVRTIHFSVR
Daldsp1    81 DN-------ESTRAVEVTINDKSIFQKQNGFRRAGLQIQGDTNENGPGTTGVRTLHFSVK
Hypospl    81 DN-------ASTKSVEVTINDKSIFQTQNGFRRAGLQIQGDTNENGPGITGVRTLHFSVK
Rosnec1    88 DN-------ASTKAVEVTINDRSIFQAQNGFRRAGLQIQGDTNEGGPGTVGIRTLHFSVK
Phamin1    80 DN-------ASHRPLEVTLSDKSVFQSQNGFRRAGLQFAGDSNNGSPGYTGIKTLHFSVK
Spobra1    86 DAAVNTSGAAAFKALEVTLSDKSIFQKQNGFRRAGLQLNGDTNTGSPGFTGIKTLHFSVK
Sposch1    86 DAAVNTSGAAAFKALEVTLSDKSIFQKQNGFRRAGLQLNGDTNTGSPGFTGIKTLHFSVK
Spoins1    97 DAAVNSSGAKPFQAVEVTLSDKSIFQKQQGFRRAGLQFNGDTNTGSPGYTGIKTLHFSVQ
Podans1    89 DD------PTYQKPFEVTINDSSIFNNQRGFRRAGLQFQGDTNRDSPGSSGIKTIHFSLK
Neucra1    83 DG-------CSHKPLEVTIADASVFKTQYGFRRAGLIFNGDKNIGSPASRGVKTLHFSVR
Neutet1    83 DS-------CSHKPLEVTIADASVFKTQYGFRRAGLIFNGDKNIGSPASRGVKTLHFSVR
Sormac1    84 DN-------CSHKPLEVTISDESVFKTQYGFRRAGLIFAADTNTGSPASKGVKTLHFSVK
Pesfic1    79 DG-------ADFKSVEVTISEKSIFQTQNGFRRAGLQLANDTNTQGPGTTGVRTLHWSVK
Psevex1    80 EN-------ASYKSIEVTISDQSIFQSQNGFRRAGLQINGDTNTGGPGSKGVKTLHWSVK
Eutlat1    82 DD-------EGFKSVEVTISDLSIFQEQNGFRRIGLQIQGDENEGGPGTVGVKTLHWSVK
Gaetri1    81 EN------ASLHKPITVTLSDRSIFQTQRGFRRAGLQFDGDSNTGGEGSAGVRTLHWSVK
Magory1    90 ED------AASHKPITVTLSDRSVFQTQRGFRRAGLQFRDDANAGGAGAVGVRTLHWSVK
Maggri1    87 EN------SSSHKPITVTLSDRSIFQTQRGFRRAGLQFKDDANTGGRGAVGVRTLHWSVK
Conlus1    91 DS----ADAAQYKPIEVNINNASVYQAQYGFRRAGLLFANDSNTGSLGYTGVKTLHWSVQ
Diaamp1    83 DN-------ASYKSVEVTISDESIFQKQYGFRRAGLLFANDTNEGSLGYQGLKTLHWSVK
Diahel1    83 DN-------ASYKSVEVTISDQSIFQKQNGFRRAGLLFANDPNEGSFGYQGLRTLHWSVR
Micbol1    98 DD------PAKHKSFEVTINDRSIFKTQFGFRRAGLLFKNDTNAAGPGNTGVKTLHWSVK
```

```
Fol-EC14  143 QDDKRPLNLSHEYLNVWHEKADFSGNQFQFQAGQLIGQ-N--GTAATWKLLDQDFKLLWE
Fuspro1   143 QDDKRPLNLSHEYLNVWHEKADFSGNQFQFQAGQLIGQ-N--GTAATWKLLDQDFKLLWE
Fusman1   143 QDDKRPLNLSHEYLNVWHEKADFSGNQFQFQAGQLIGQ-N--GTAATWKLLDQDFKLLWE
Fusfuj1   143 QDDKRPLNLSHEYLNVWHEKADFSGNQFQFQAGQLIGQ-N--GTAATWKLLDQDFKLLWE
Fusnyg1   143 QDDKRPLNLSHEYLNVWHEKADFSGNQFQFQAGQLIGQ-N--GTAATWKLLDQDFKLLWE
Fusver1   143 QDDKRPLNLSHEYLNVWHEKADFSGNQFQFQAGQLIGQ-N--GTAATWKLLDQDFKLLWE
Fuscul1   140 QDKSRPLNLSHEYINVWHEKADFSGNQFQFQSGTIIGQ-N--HTAATWKLFDEKMKLLWE
Fusgra1   140 QDKSRPLNLSHEYINVWHEKADFSGNQFQFQSGTIIGQ-N--HTAATWKLFDEKMKLLWE
Fuspse1   140 QDKSRPLNLSHEYLNVWHEKADFSGNQFQFQSGTIIGQ-N--HTAATWKLFDEKMKLLWE
Fuslan1   140 QDKSRPLNLSHEYLNVWHEKADYSGNQFQFQAGTIIGQ-N--HAASTWKLFDEKMKLLWE
Fuspoa1   140 QDKNRPLNLSHEYLNVWHEKADYSGNQFQFQAGTIIGQ-N--HAAATWKLFDEKMKLLWE
Fusven1   140 QDKNRPLNLSHEYLNVWHEKADFSGNQFQFQAGTIIGQ-G--HAAATWKLFDEKMKLLWE
Neodit1   142 QDESRPLNLSHEYLNVWHEAADYSSNQFNFQTGAMIDQPT--LPKDTYKLLDRTNKEIWS
Fussol1   143 QDESRPLNLSHEYLNVWHEAGDYSSNQFNFEAGTIIGQ-D--LPKDTWKLLDRTNKQIWS
Colchl1   139 QDPSRPLNLTHEYLNVWHETAAYDANQFSFQAGTIFGQAG--FPKDTFKLLDRDNKLVWS
Colfio1   148 LNSERPLNLTHEYLNVWHETAAYDANQFNFQTGTIIGQPR--LPKDTFKLLDRDNKLLWS
Colnym1   148 LDLERPLNLTHEYLNVWHETAAYDANQFNFQTGTIIGQPE--LPKDTYKLLDRDNKLLWS
Colsim1   148 LDLERPLNLTHEYLNVWHETAAYDANQFNFQTGTIIGQPA--LPKDTFKLLDRDNKLLWL
Colglo1   135 QDAERPLNLTHEYLNVWHETAAYDANQFNFQTGTIIGQSG--LPEDTFKLLDRENRLVWS
Colgra1   149 QDAARPLNLTHEYLNVWHETAAYDANQFNFQTGTLIGREG-LLPKDTFKLLDRDNKLLWS
Colinc1   139 QDAAKPLNLTHEYLNVWHETAAYDANQFNFQTGTIIGQAG--LPKNTFKLLDRNNKLVWS
Coltof1   139 QDPARPLNLTHEYLNVWHETAAYDANQFNFQTGTIIGQAG--LPKNTFKLLDRDNKLVWS
Colhig1   141 QDAARPLNLTHEYLNVWHETAAYDANQFSFQTGTIIGQPG--LPRDTFKLLDRDNRLVWS
Colorb1   135 QDIERPLNLTHEYLNVWHETAAYDANQFNFQTGTIIGQEG--LPEKTWKLLDRNNQLMWS
Veralf1   141 TDAQRRFNESHEYLNVWHETAVFDANQFNFQTGTIIGQPE--LPANTWKLLDRNNALLWS
Verdah1   141 TDAQRRFNESHEYLNVWHETAAFDANQFNFQTGTIIGQPE--LPANTWKLLDRNNALLWS
Verlon1   141 TDAQRRFNGSHEYLNVWHETAAFDANQFNFQTGTIIGQPE--LPANTWKLLDRNNALLWS
Lompro1   134 TDPQRTLNLTHEYILVWHETAAYDANQFNFQTGTILGQTG--LPAETYKLLDRSNKQLWS
Sceapi1   134 TDPQRALNLTHEYILVWHETAAYDANQFNFQTGTILGQTG--LPRDTYKLLDRNNKQLWS
Chathe1   145 WDNARPLNLSHEYLNVWHETADYSANQFNFEGGAILGQTQ--LPADTWKVLDRNNRQIWA
Madmyc1   143 WDAQRALNLSHEYLNVWHEAADYSANQFNFQAGAILGQSS--LPRDTYKVLNRQNRQVWS
Thiter1   141 LDPQRPLNLSHEYLNVWHESADYSANQFNFEAGTIIGQSG--LPKNTWKVLNRQNQQIWS
Thethe1   141 IDPQRGLNLSHEYILVWHEAADYSANQFNFETGTIIGQEG--LPKDTYKVLNRNNQQIWS
Conlig1   135 QDPKRPLNLTHEYLNVWHEASDYSADQFMFEAGTIIDQTG--LPKNTFKVLNRQSKQIWS
Daldsp1   134 QDPERTLNLTHEYLNVWHESADYSANQFNFQSGTIIGKSG--SDKNNFKILNRQNTQVWS
Hyposp1   134 QDPERALNLTHEYLNVWHETSDYSANQFNFEAGTIIGKSG--SDKNSFKVLNRQNTQVWS
Rosnec1   141 QDPQRRLNLTHEYLNVWHETADYSANQFNFQAGTLIGRNS---NKNTFKVLNRQNTEVWS
Phamin1   133 QDPARALNLSHEYLNVWHEAADYSADQIMFETGTILGQTG--LPKNTFKFFDRNMKQLWS
Spobra1   146 QDAQRPLNLSHEYLNVWHEASDYSADQIMFQAGTLIDQAG--LPKNTFKVMSRQNKQLWS
Sposch1   146 QDAQRPLNLSHEYLNVWHEASDYSADQIMFQAGTLIDQAG--LPKNTFKVMSRQNKQLWS
Spoins1   157 QDAQRPLNLSHEYLNVWHESSDYSADQIMFQTGTLIDQAG--LPKNTWKVLSRTNKLLWS
Podans1   143 WDAQRPLNLSHEYLNVWHETADYSANQFNFQAGAILGQNS--LARDTWKVLNRQNRQVWS
Neucra1   136 QDEARGLNLTHEYLNVWHETSDWSANQFNFETGTIIGQEG-SLAKNTFKIMSRENKLIWS
Neutet1   136 QDEARGLNLTHEYLNVWHETSDWSANQFNFQTGTIIGQEG-SLAKNTFKIMSRENKLIWS
Sormac1   137 QDLKRPLNLTHEYLNIWHETSDWSANQFNFQTGTIIGQDTRKLPKNTFKIMSRENKLIWQ
Pesfic1   132 QDPTRTLNLTHEYLNVWHERADYNGNQFNFETGTLIDNSS--LDKNTFKITNRNSEVIWS
Psevex1   133 QDSSRPLNLTHEYLNVWHERADYNGNQFNFQTGTVIGQ-S--YAKDTFKILDRQNVLVWS
Eutlat1   135 QDPARTFNLTHETYGM--RRARYDGNQFNFETGTIIGRPG---NENTFKILDRQNTEVWS
Gaetri1   135 IDPGRALNLSHEYLNVWHETSDYSANQFNFEMGTILGQTS--LPRDTFKILNRQNRQIWS
Magory1   144 IDAARGLNLSHEYLNVWHETADYSANQFNFEMGSILGQPE--LPSDTFKILDRRNRQIWS
Maggri1   141 IDPARGLNLSHEYLNVWHETADYSANQFNFEMGSILGQPD--LPSDTFKILDRRNQQIWS
Conlus1   147 QDPQRALNLSHEYLNVWHESADYSADQIMFQAGSILAQPG--LARDTFKVFDRNSSLLWS
Diaamp1   136 QDSARALNLSHEYLNVWHESADYSGDQFMFQTGAMLDYPD--MPSDTFKFFDRNATLLWS
Diahel1   136 QDPALALNLSHEYLNVWHERADYSANQFSFQTGLMIDHPE--LSADSFKFFDRDGRLIWS
Micbol1   152 QDPARPLNLTHEYLNVWHEAGDFSSNHFHFTAGSLIGRPE--FPANTFKILNRKLDVIFS
```

```
Fol-EC14  200 TPMLKK-VWQNFAITLNYEKNTIQAYYS-KGCKPLKVAT-QPIARNLTGQGQFQIGILKK
Fuspro1   200 TPMLKK-VWQNFAITLNYEKNTIQAYYS-KGCKPLKVAT-QPIARNLTGQGQFQIGILKK
Fusman1   200 TPMLKK-VWQNFAITLNYEKNTIQAYYS-KGCKPLKVAT-QPIARNLTGQGQFQIGILKK
Fusfuj1   200 TPMLKK-VWQNFAITLNYEKNTIQAYYS-KGCKPLNVAT-QPIARNLTGQGQFQIGILKK
Fusnyg1   200 TPMLKK-VWQNFAITLNYEKNTIQAYYS-KGCKPLKAAT-QPIARNLTGQGQFQIGILKK
Fusver1   200 TPMLKK-VWQNFAITLNYEKNTIQAYYS-KGCKPLKAAT-QPIARNLTGQGQFQIGILKK
Fuscul1   197 TPMLEG-KWQNFAITLNFDKNTIQAYYS-EGPKALKAAT-QPIARDLSGQGQYQIGMLKK
Fusgra1   197 TPMLEG-KWQNFAITLNFDKNTIQAYYS-EGSKALKAAT-QPIARDLSGQGQYQIGMLKK
Fuspse1   197 TPMLEG-KWQNFAITLNFDKNTIQAYYS-EGSKALKAAT-QPIARDLSGQGQYQIGMLKK
Fuslan1   197 TPMLKG-KWQNFAITLNFDKNTIQAYYS-EGSKPLKAAT-KPIARDLSGQGQYQIGMLKK
Fuspoa1   197 TPMLKG-KWQNFAITLNFDKNTIQAYYS-EGSKPLKAAT-KPIARDLSGQGQYQIGMLKK
Fusven1   197 TPMLKA-KWQNFAITLNFDKNTIQAYYS-EGSKPLKAAT-KPIARDLSGQGQYQIGMLKK
Neodit1   200 TPILPG-SWQNFAITLDFNNNTLQAWYS-EGNKPLLPAT-KPVANDNSGRGQYQIGILKK
Fussol1   200 TPILKD-VWQNFAITLDFDNNTIQAWYS-EGRKPLRAVT-EPISNDNSGRGQYQIGILKK
Colchl1   197 TPIVTDGSWQNFALTLDFSNNTMQVWYS-AGDAPLTKQG-SIISANLAGEGQYQIGILKK
Colfio1   206 TPMAMDGSWQNFALTLDFLYNTIQIWYS-AGDNPLARQG-GGVPVDLAGDGQYQIGILKK
Colnym1   206 TPIAKDGSWQNFALTLDFLNNTIQIWYS-AGNNPLARQG-GAVPVNLAGDGQYQIGILKK
Colsim1   206 TPMAKDGSWQNFALTLDFLNSTIQIWYS-AGNNPLARQG-GAVPVDLAGDGQYQIGILKK
Colglo1   193 TPMATDGSWQNFALTLDFDQNTIQVWYS-ANDEPLQKSG-DIISANLAGQGQYQIGMLKK
Colgra1   208 TPVATDGSWQNFALTLDFSKNTIQVWYS-TGGARLASQG-GAIPANLAGDGQYQIGILKK
Colinc1   197 TPIATDGSWQNFALTLDFSKNTIQVWYS-AGDAPLVKQG-GVISANLTGDGQYQIGILKK
Coltof1   197 TPMATDGSWQNFALTLDFSNNTIQAWYS-AGDAPLIKQG-GVVSANLAGDGQYQIGILKK
Colhig1   199 TPVATDGSWQNFALTLDFPNNTIQVWYS-AGNAPLARQG-GVIAANLLGDGQYQIGLLKK
Colorb1   193 TPLVDDGSWQNFALTLDFDQNTIQVWYS-GGGAPLTKQG-DGILADLAGGGQYQIGILKK
Veralf1   199 TPVLDG-VWQNFGLTLDFNANTIGVLYS-EGDKPLAPSGKGAFPANLAGEGQYHVGILKK
Verdah1   199 TPVVDG-VWQNFALTLDFNANTISVLYS-EGDKPLAPSGKGAFPANLAGEGQYHVGILKK
Verlon1   199 TPVLDG-VWQNFGLTLDFNANTIGVLYS-EGDKPLAPSGKGSFPANLAGEGQYHVGILKK
Lompro1   192 TPIKDD-TWQNFAITIDYTANTLQMWYS-EGDAELAKAT-DPINNNNAGEGQYQVGMLKK
Sceapi1   192 TPIKSD-TWQNFGITVDYTGNTLQVWYS-EGDEELAKAT-DPINNNNAGEGQYQVGMLKK
Chathe1   203 VKIERE-GWQNFAITVDYEKNTLQVYYS-KDDEPLTAVT-SAVYNNNAGEGQFQIGILKK
Madmyc1   201 TPILRD-DWQNFAITVDFNRNTLQVYYS-EGDEPLRSVT-SAVSNNNAGEGQYQIGLLKK
Thiter1   199 TPIVKD-DWQNFAITLDFNKNTLQVYYS-QGTDPLKSVT-SAVSNDNSGEGQYQIGILKK
Thethe1   199 TPILED-QWQNFAITLDFNQNTLQVYYS-EADEPLESVT-DVVSNNNAGEGQYQIGLLKK
Conlig1   193 TPIEST-AWQNFAITLDFNKNTLQVYYS-KGDDPLKSVT-SAVSNDNSGGGQYQIGILKK
Daldsp1   192 TPIDAT-AWQNFAITLDFVKNTLQVYYS-KDDEPLKAVT-EALQNDNSGGGQYQIGILKK
Hypospl   192 TPIDAT-AWQNFAITLDFVKNTLQVYYS-KDDEPLKAVT-SALQNDNSGGGQYQIGILKK
Rosnec1   198 TPIDET-AWQNFAITLDFNKNTLQVYYS-RGDEPLRSVT-SALSNNNAGGGQYQIGILKK
Phamin1   191 TAVDGT-AWQNFAVTLNINSNTVQVYYS-KDDEPLKSVT-SAVSADLSGQGQFQIGILKK
Spobra1   204 TPISTT-DWQNFAFTLDFNKNTVQVYYS-KGTDPLASVL-KATADDLSGAGQFQIGILKK
Sposch1   204 TPISTT-DWQNFAFTLDFNKNTVQVYYS-KGTDPLASVL-KATADDLSGAGQFQIGILKK
Spoins1   215 TPLVAA-DWQNFALTLDFNKNTVQVYYS-QGSDPLKSVL-TATADDLTGAGEYQIGILKK
Podans1   201 TPILRN-EWQNFAISLDFVRNTLRVYYS-RGSEPLRSVT-NALTNNNAGEGQYQVGILRK
Neucra1   195 TPIEQK-EWQNFAITLDFNKNTLRLYYS-KGHEPLRSVT-QAVPNNNAGEGQYQIGILKK
Neutet1   195 MPIEQK-EWQNFAITLDFDKNTLRLYYS-KGHEPLRSVI-QAVPNNNAGEGQYQIGILKK
Sormac1   197 TPIEKK-EWQNFAITLDFNKNTLQVYYS-KGNEPLRSVT-KAVPNNNAGEGQYQFGILKK
Pesfic1   190 TPIDQS-AWQNFAVTLDYDNNTLQVYYS-LGTEPLTAVT-QALANDNSGYGQFQIGILKK
Psevex1   190 TPIDAT-AWQNFAVTLDFDKNTLQVYYS-EGDAALAAVT-NVLTNDNSGEGQYQFGILKK
Eutlat1   190 TPIDES-EWQNFAVTLDFEQNTLQVYYS-VGDTPLEAVT-EALSNNNAGEGQYQIGILKK
Gaetri1   193 TKVDNT-AWNNFAIKIDYTKNTLQVYYS-KGDEPLKSVT-NAVQNDNSGKGQYQLGLLKK
Magory1   202 TPIDET-AWNNFAVTIDYVKNTLRVYYS-KNDEPLKAASCAPTPNDNSGEGQYQLGLLKK
Maggri1   199 TPIDET-AWNNFAVTIDYDKNTLRIYYS-KNDEPLKAASCTASPNDNSGEGQYQLGLLKK
Conlus1   205 VAMDLEGGWQNFAVTLDVTANTIQIYYS-TGTDPLQAVT-DVIPTDLSGDGQFQIGMIKK
Diaamp1   194 TPIDFS-EWQNFAITLDIDSNLVQIYYS-LGNAALEAVT-DFVPADLSGEGQFQLGILKK
Diahel1   194 TPIDFE-EWQNFAVMLNVDANLIQIYYS-LGDAALEAVT-DAEPLRLEGEGQYQLGILKK
Micbol1   210 TPINLRGEWQTFAVTLDYNANTIQVFTSPNANTPLKPAFPTPLPNDNSGFGQFQMGILKK
```

```
Fol-EC14 257 PTG-TDDVANSGFQEANLNEGLIYGGIFLED-SADGCVSL
Fuspro1  257 PTG-TDDVANSGFQEANLNEGLIYGGIFLED-SADGCVSL
Fusman1  257 PTG-TDDVANSGFQEANLNEGLIYGGIFLED-SADGCVSL
Fusfuj1  257 PTG-TDDVANAGFQEANLNEGLIYGGIFLED-SADGCVSL
Fusnyg1  257 PTG-TDDVANSGFQEANLNEGLIYGGIFLED-SADGCVSL
Fusver1  257 PTG-TDDVANSGFQEANLNEGLIYGGIFLED-SADGCVSL
Fuscul1  254 PTG-TDDVANAGFQEANLNEGLIYGGIFLED-SKNNCVSL
Fusgra1  254 PTG-TDDVANAGFQEANLNEGLIYGGIFLED-SKNNCVSL
Fuspse1  254 PTG-TDDVANAGFQEANLNEGLIYGGIFLED-SKNNCVSL
Fuslan1  254 PTG-TDDVANAGFQEDNLNEGLIYGGIFLED-SKNDCVSL
Fuspoa1  254 PTG-TDDVANAGFQENNLNEGLVYGGIFLED-SKNDCVSL
Fusven1  254 PTG-TDDVANAGFQEDNLDEGLIYGGIFLED-SKNDCVSL
Neodit1  257 PTG-TADVVNSGFQESGLNEGLIYGGIFIED-STNYCVSK
Fussol1  257 PTG-TDDVVNSGFQESDLDEGLIYGGIFIED-SKDGCVSA
Colchl1  255 PTG-TSDVVNSGYQSSNLDEGQIYGGIFIED-SADGCVSI
Colfio1  264 PTG-TDDVVNSGYQSSNLNEGLIYGGIFIED-SADGCVSI
Colnym1  264 PTG-TDDVVNSGYQSSNLNEGLIYGGIFIED-SANGCVSI
Colsim1  264 PTG-TDDVVNSGYQSSNMNEGLIYGGIFIED-SANGCVSI
Colglo1  251 PTG-TDDVVNSGYQESDLDEGQIYGGIFIED-SADGCVSV
Colgra1  266 PTG-TSDVVNSGYQSSNLDEGQIYGGIFIED-GADGCVSL
Colinc1  255 PTG-TSDVVNSGYQSSNLNEGQIYGGIFIED-SADGCVST
Coltof1  255 PTG-TSDVVNAGYQSSNLNEGQIYGGIFIED-STNGCVST
Colhig1  257 PTG-TSDVVNEGYQSNNLDEGQIYGGIFIEE-SAGGCVST
Colorb1  251 PTG-TSDVVNSGYQASNLDEGQVYGGIFIED-STGGCVSI
Veralf1  257 PTG-TDDVVNSGFQESPLNEGLIYAGVFIED-SANGCVSK
Verdah1  257 PTG-TDDVVNSGFQESPLNEGLIYAGVFIED-SANGCVSK
Verlon1  257 PTG-TDDVVNSGFQESPLNEGLIYAGVFIED-SANGCVSK
Lompro1  249 PTG-TSDVVNSGYQESGLDEGLIYGGIFIED-SASDCVSK
Sceapi1  249 PTG-TSDVVNSGYQESGLDEGLIYGGIFIEDSSADTCVSK
Chathe1  260 PTG-TSDVVNSGYQSPNLDEGLLYGGLFVED-SAGGCVSL
Madmyc1  258 PTG-TSDVVNSGYQESNLNEGLIYGSLFVED-SADGCISL
Thiter1  256 PTG-TSDVVNSGYQESNLNEGLIYGSLFVED-SANGCISL
Thethe1  256 PTG-TDDVVNSGYQESGIDEGLIYGSLFVED-SANGCVSL
Conlig1  250 PTG-TSDVVNAGYQESKLNEGQIYGGLFIED-STNGCVSL
Daldsp1  249 PTG-TSDVVNSGYQETGINEGQIYGGIFLED-SANGCISL
Hyposp1  249 PTG-TSDVVNSGYQETGISEGQIYGGIFLED-SANGCVSL
Rosnec1  255 PTG-TSDVVNGGYQEKNLDEGQIYGGLFLED-GANGCISL
Phamin1  248 PTG-TSDVVNSGYQESGLNEGQIYGGLFIED-SSNGCISL
Spobra1  261 PTG-TSDVVNSGFQESGLKEGQIYGGIFIED-SANGCISL
Sposch1  261 PTG-TSDVVNSGFQESGLKEGQIYGGIFIED-SANGCISL
Spoins1  272 PTG-TSDVVNSGFQESGLKEGLIYGGIFIED-SANGCVSL
Podans1  258 PTG-TSDVVNSGYHQRNLNEGLIYGSVFVED-GEGGCVSL
Neucra1  252 PTG-TDDVVNSGYQESDIDEGQIYGGIFIED-SANGCVSL
Neutet1  252 PTG-TDDVVNSGYQESGIDEGQIYGGIFIED-SANGCVSL
Sormac1  254 PTG-TDDVVNKGYQESGIDEGQIYGGIFIED-SAGGCVSL
Pesfic1  247 PTG-TSDVVNSGYQESPISEGQIYGGIFLED-SANGCITL
Psevex1  247 PTG-TSDVVNSGYQEKGILEGQIYGGIFLED-SADGCISL
Eutlat1  247 PTG-TDDVVNSGYQETGIDEGQIYGSIFLED-SSDGCVSL
Gaetri1  250 PTG-TNDVVNSGYQSRNINEGLIYGGIFLED-SAGGCISL
Magory1  260 PTG-TDDVVNSGYQASGIDEGLIYGGIFLED-SADGCVSL
Maggri1  257 PTG-TDDVVNSGYQASGIDEGLIYGGIFLED-SADGCVSL
Conlus1  263 PTG-TSDVVNAGYQEAPFEEGQIYGGLFVED-SAGGCISL
Diaamp1  251 PTG-TDDVVNSGFQESPLNEGQIYGGIFVED-STGGCVSL
Diahel1  251 PTG-SADVVNSGFQESPLNEGQIYGGIFVED-STAGCVSL
Micbol1  270 PTGETTDIVNKGFQSAGIDEGQIYGGVFLED-SAGGCVSL
```

<u>Sordariomycetes</u>

Hypocreomycetidae - Glomerellales

Colchl1 = OLN97223.1 hypothetical protein CCHL11_02057 [Colletotrichum chlorophyti] after splicing out intron with putative non-canonical GCGCGT donor site, consistent with the findings of Rep et al. (2006)

Colfio1 = EXF74738.1 hypothetical protein CFIO01_05408 [Colletotrichum fioriniae PJ7]

Colglo1 = EQB57524.1 hypothetical protein CGLO_02337 [Colletotrichum gloeosporioides Cg-14]

Colgra1 = XP_008095341.1 hypothetical protein GLRG_06465 [Colletotrichum graminicola M1.001]

Colhig1 = XP_018153158.1 hypothetical protein CH63R_11343 [Colletotrichum higginsianum IMI 349063]

Colinc = KZL79130.1 hypothetical protein CI238_10245 [Colletotrichum incanum]
Colnym = KXH49624.1 hypothetical protein CNYM01_07617 [Colletotrichum nymphaeae SA-01]
Colorb = TDZ26553.1 hypothetical protein Cob_v001237 [Colletotrichum orbiculare MAFF 240422]
Colsim = KXH49371.1 hypothetical protein CSIM01_11916 [Colletotrichum simmondsii]
Coltof = KZL70738.1 hypothetical protein CT0861_13067 [Colletotrichum tofieldiae]
Veralf = XP_003005433.1 conserved hypothetical protein [Verticillium alfalfae VaMs.102]
Verdah = XP_009658131.1 uncharacterized protein VDAG_10367 [Verticillium dahliae VdLs.17]
Verlon = KAG7105666.1 hypothetical protein HYQ44_015124 [Verticillium longisporum]
Hypocreomycetidae - Hypocreales - Clavicipitaceae - none
Hypocreomycetidae - Hypocreales - Cordycipitaceae - none
Hypocreomycetidae - Hypocreales - Hypocreaceae - none
Hypocreomycetidae - Hypocreales - Nectriaceae
Fuspro = XP_031082440.1 related to endoglucanase c [Fusarium proliferatum ET1]
Fusfuj = KLO84549.1 endoglucanase c [Fusarium fujikuroi]
Fusman = XP_041680198.1 uncharacterized protein FMAN_08678 [Fusarium mangiferae
Fuscul = PTD05001.1 hypothetical protein FCULG_00002645 [Fusarium culmorum]
Fusgra = XP_011319797.1 hypothetical protein FGSG_08907 [Fusarium graminearum PH-1]
Fuslan = KPA46823.1 endoglucanase c [Fusarium langsethiae]
Fusnyg = PNP82107.1 hypothetical protein FNYG_04560 [Fusarium nygamai]
Fuspoa = KAG8671804.1 hypothetical protein FPOAC1_005062 [Fusarium poae]
Fuspse = KAF0636074.1 hypothetical protein FPSE5266_08205 [Fusarium pseudograminearum]
Fusven = XP_025584865.1 uncharacterized protein FVRRES_05581 [Fusarium venenatum]
Fusver = XP_018746004.1 hypothetical protein FVEG_02506 [Fusarium verticillioides 7600]
Neodit = KPM34483.1 hypothetical protein AK830_g12087 [Neonectria ditissima]
Fussol = XP_003052800.1 uncharacterized protein NECHADRAFT_67676 [Fusarium vanettenii 77-13-4] (syn. Fusarium solani)
Hypocreomycetidae - Hypocreales - Ophiocordycipitaceae - none
Hypocreomycetidae - Hypocreales - Stachybotryaceae - none
Hypocreomycetidae - Microascales
Ceratocystis platani - no orthologue
Thielaviopsis punctulate - no orthologue
Lompro = PKS06492.1 hypothetical protein jhhlp_007240 [Lomentospora prolificans]
Sceapi = XP_016644335.1 hypothetical protein SAPIO_CDS3558 [Scedosporium apiospermum]
Sordariomycetidae - Coniochaetales
Conlig = OIW34589.1 hypothetical protein CONLIGDRAFT_650979 [Coniochaeta ligniaria NRRL 30616]
Sordariomycetidae - Togniniales
Phamin = XP_007912622.1 hypothetical protein UCRPA7_1852 [Phaeoacremonium minimum UCRPA7]
Sordariomycetidae - Diaporthales
Conlus = PSR86977.1 hypothetical protein BD289DRAFT_433121 [Coniella lustricola]
Diaamp = KKY30718.1 hypothetical protein UCDDA912_g09348 [Diaporthe ampelina]
Diahel = POS74193.1 hypothetical protein DHEL01_v207411 [Diaporthe helianthi]
Sordariomycetidae - Magnaporthales
Gaetri = XP_009230278.1 hypothetical protein GGTG_14087 [Gaeumannomyces tritici R3-111a-1]
Magory = ELQ60567.1 hypothetical protein OOW_P131scaffold01278g3 [Pyricularia oryzae P131] (syn. Magnaporthe oryzae)
Maggri = XP_030977943.1 uncharacterized protein PgNI_09303 [Pyricularia grisea] (syn. Magnaporthe grisea)
Sordariomycetidae - Ophiostomataceae
Grosmannia clavigera - no orthologue
Ophiostoma piceae - no orthologue
Spobra = XP_040622012.1 uncharacterized protein SPBR_06066 [Sporothrix brasiliensis 5110]
Spoins = OAA67127.1 hypothetical protein SPI_01703 [Sporothrix insectorum RCEF 264]
Sposch = XP_016586072.1 hypothetical protein SPSK_04444 [Sporothrix schenckii 1099-18]
Sordariomycetidae - Sordariales - Sordariales incertae sedis
Madmyc = KXX74097.1 hypothetical protein MMYC01_208909 [Madurella mycetomatis]
Sordariomycetidae - Sordariales - Chaetomiaceae
Chathe = XP_006697564.1 hypothetical protein CTHT_0073070 [Chaetomium thermophilum var. thermophilum DSM 1495]
Podans = XP_001913072.1 uncharacterized protein PODANS_1_8450 [Podospora anserina S mat+] with intron prediction corrected
Thethe = XP_003659079.1 uncharacterized protein MYCTH_2295704 [Thermothelomyces thermophilus ATCC 42464]
Thiter = XP_003652516.1 uncharacterized protein THITE_2114094 [Thermothielavioides terrestris NRRL 8126] (syn. Thielavia terrestris)

Sordariomycetidae - Sordariales - Sordariaceae
Neucra = KHE89943.1 glycoside hydrolase family 131 protein [Neurospora crassa] after splicing out intron with non-canonical GCATGT donor site described by Rep et al. (2006) and removing spurious N-terminal sequence preceding correct start methionine
Neutet = EGZ77998.1 hypothetical protein NEUTE2DRAFT_80122 [Neurospora tetrasperma FGSC 2509] after splicing out intron with non-canonical GCATGT donor site described by Rep et al. (2006) and removing spurious N-terminal sequence preceding correct start methionine
Sormac = XP_024511214.1 uncharacterized protein SMAC_05957 [Sordaria macrospora k-hell]
Xylariomycetidae - Xylariales
Eutlat = EMR68142.1 hypothetical protein UCREL1_4850 [Eutypa lata UCREL1]
Daldsp = OTB14174.1 hypothetical protein K445DRAFT_140233 [Daldinia sp. EC12]
Hyposp = OTA63122.1 glycoside hydrolase family 131 protein [Hypoxylon sp. EC38]
Micbol = KXJ85933.1 hypothetical protein Micbo1qcDRAFT_220044 [Microdochium bolleyi]
Pesfic = XP_007828005.1 hypothetical protein PFICI_01233 [Pestalotiopsis fici W106-1]
Psevex = XP_040712670.1 uncharacterized protein BCR38DRAFT_398434 [Pseudomassariella vexata]
Rosnec = GAP92008.2 putative glycoside hydrolase family 131 protein [Rosellinia necatrix] corrected to remove spurious N-terminal sequence

Supplementary Figure S6. Fol-EC14 alignment. Multiple protein-sequence alignment of Fol-EC14 orthologues in the Fol-EC14 clade of Sordariomycete GH131 (glycosyl-hydrolase 131) sequences. All sequences shown were the highest blastP matches to Fol-EC14 obtained for each genome-sequenced species represented. Sequences were aligned using ClustalW at https://www.ebi.ac.uk/Tools/msa/mafft/. Amino-acid identities were highlighted in black and similarities in grey using BoxShade at https://embnet.vital-it.ch/software/BOX_form.html. Conserved cysteines have been highlighted manually in blue, residues conserved with the catalytic residues of PaGluc131A and CcGH131A in red and non-conserved residues in teal.
