# Supplementary material for: Transcriptome Analysis of Fusarium–Tomato Interaction Based on an Updated Genome Annotation of Fusarium oxysporum f. sp. lycopersici Identifies Novel Effector Candidates That Suppress or Induce Cell Death in Nicotiana benthamiana
Source: J Fungi (Basel). 2022 Jun 26;8(7):672. doi: 10.3390/jof8070672 (PMC9316272; doi:10.3390/jof8070672)
Supplement: Supplementary file 1 [file jof-08-00672-s001.zip › Supplementary Materials/Supplementary Figures/Supplementary Figure S7.pdf]

```
Colhig2   1 MYTSTLIL--SLVAAAAAACTGG--KNKTAE--AAAPGAKA-QCALQFDGRIPKAFTAAD
Colchl2   1 MFTSTLFL--SLVAAAAAACNGK--PNDT----DSSSGVKG-QCALQFDGRIPKAFTAAD
Colfio2   1 MFASTIFL--SLVAAAAAACDGTTPKNNTG---AVSPGAKA-QCALQFDGRIPKTFKAAD
Colsim2   1 MFASTIFL--SLVAAAAAACDGTTPKNNTG---AVSPGAKA-QCALQFDGRIPKTFKAAD
Colinc2   1 MFTSTLFL--SLVAAAAAACDGST-KNNTA---GATPGAKA-QCSLQFDGRIPKAFTAAD
Coltof2   1 MFTSTLFL--SLVAAAAAACDGST-KNNTA---GATPGAKA-QCSLQFDGRIPKAFTAAD
Colgra2   1 MYTSTLIF--SLLAAA---------------------GAKAQQCTLQFDGRIPKASTVAD
Colorb2   1 MFASTIIL--SLVAAVAADCGSKS-GNNSYSSGPALPGAQA-QCTLQFDGRIPKAFTAAD
Lompro2   1 MALSRS----ALLSILLSAA----------------PLVSAQECTLQFDGRVPDGFAAAD
Sceapi2   1 MSPRS-----ALLSLLLSAL----------------PLVSAQECTLQFDGRIPDGFAAAD
Rosnec2   1 MAGFYPL---ALLVGIIPTL------------------VVSQQCDLQFDGRVPSDFVAAT
Conlig2   1 MASPYLLSVSSILTLLALTS--------------------AQKCPLQFDGRIPSTFTPTS
Grocla2   1 MLARH-----SLVLLASATT-----------------AVLAKTCALQFDGRVPSTFNLTT
Ophpic2   1 MISQYL----SLLALPVLAA--------------------AQKCSLQFDGRIPSNLTLAA
Spobra2   1 MMYPLSI---AFLALPLLAS--------------------AQKCTIQFDGRVPSTFTAAS
Spoins2   1 MRSSASL---ALLALPLLAS--------------------AQKCAIQFDGRIPSSFSAAT
Phamin2   1 MASLIRNY-FSLLPILLELS-----------------VVLAQSCDIQFDGRVSAGSAVAD
Podans2   1 MHKHGI----PLLFASLVTS------------------VSAQSCRLHFDGRVPPSFGVAG
Diaamp2   1 MFSQRLLT-QTTLVSLASLA--------------------ASQSAIQFDGRVPAGTQLSA
Diahel2   1 MFSQRLLT-QTSLFSLASLA--------------------ASQSTIQFDGRVPAGTQLSA
Psevex2   1 MASHRSFI-PSFLSLVTLVV--------------------AQQCTLQFDGRVPSDFVAAT
Thiter2   1 MVHLQYY---SVAVSLLLPS-----------------WVAAQNCTLQFDGRVPSDLVAAD
Gaetri2   1 MRYSFTST-LSLALSLSPIS-----------------GVWAQCRKLQFDGRIPNNFQVAQ
Magory2   1 MRSFTVAS--AIFGGLLATS-----------------PVYGQQCKLQFDGRVPANFQPAN
Maggri2   1 MRSFTVAS--AILGGLSATS-----------------PVYGQQCKLQFDGRIPENFELTG
Cerpla2   1 MQSL------SLLALIAPVA--------------------AQVCQLAFDGRVAKDFALEG
Thipun2   1 MYSR------SLIAAALAAA-----------------PAVAQVCPLAFDGRVPDNFTLDT
Colglo2   1 MSLSFIST-LSLLLSAAGAAN------------------AAKQCPIQFEGRVPTNFTPTD
Colnym2   1 MHFSSSSL-GALVAVAAGAAD-------------------AAKCPIQFDGRIPTNFTPAT
Stacha2   1 MAPSLFRL-LPVLAGVTAVA--------------------GQSCPLQFDGRIPRNFTVAD
Stachl2   1 MAPSLFRI-LPVLAGVTAVA--------------------GQSCSLQFDGRIPRNFTVAD
Clapur2   1 MRTYFAI---NFVAVATAAASG-----------LGREAATTTKCPLVFDGRVPANFTLEQ

Colhig2  54 FDG-KTSPFNTANVFGKGLKFSDLIK-LPGGQ----SL--FDV-NSTQPFEVTI------
Colchl2  52 FDG-KSSPFNTANVFGKGLKFSDLIK-LPGGQ----SL--FDV-NSTQPLEVTI------
Colfio2  55 FDS-TSSPFNTANVFGKGLKFSEIVQ-LPGGN----SL--FDV-NSTQPFEVTI------
Colsim2  55 FDS-TSSPFNTANVFGKGLKFSEIVQ-LPGGN----SL--FDV-NSTQPFEVTI------
Colinc2  54 FDG-SSSPFNTANVFGKGLKFSDLVQ-LPGGQ----SL--FDV-NSTQPFEVTI------
Coltof2  54 FDG-TSSPFNTANVFGKGLKFSDLVQ-LPGGQ----SL--FDV-NSTQPFEVTI------
Colgra2  38 FDA-KTSPFNPSNVFGQGLKFSQLIQ-LPGGQ----SL--FDA-NATQPFEVTI------
Colorb2  57 FDG-KTSPFNADNVIGKGLKFSELIK-LPEET----SL--FDG-NSSKPFEVTI------
Lompro2  41 FDA-ANDIFSPDNVFGQGLSFGQLIQ-IPNAGL---SI--FDD--GTIPFEVTI------
Sceapi2  40 FDQ-ANNIFSPDNVFGQGLAFSQLIQ-LPAIGA---SL--FDD--ATVPFEVTI------
Rosnec2  40 FNT-GNGVFDPNNVVGQGLSLGSVVQ-IQDSLS---SL--FDV--GTNPVEVTI------
Conlig2  41 FDA-ANAFFSQSNVLGQGLKFSQLIQ-LPSITP---SL--FDT--ATVPVGVTI------
Grocla2  39 FDT-ANGVFNPNNVFGKNLTLSQLLR-LPAQKG---SI--FDE--NTVPFEVTI------
Ophpic2  37 FDS-NNNIYNPSNVFGKGLKFSELIQ-LPAVAG---SL--FDT--KTVPVEVTI------
Spobra2  38 FDT-NNNLFNPSNVFGQGLKFSQLIQ-LPAVTG---SL--FDV--NTVPAEVTI------
Spoins2  38 FDT-NNNVFSPSNVFGQNLKLSQLVE-LPAQTG---SL--FDV--DTVPVEVTI------
Phamin2  43 FDA-TNNLFSTDNVFGQGLKFSQLLE-LPAVTP---SLPKFDV--NTIPVEVTI------
Podans2  39 FDT-PNNFFSETNVLGAGLSFSQLIQ-LPAISA---SL--FDI--ETIPIEVTI------
Diaamp2  40 FDS-ENGLFNPDNVFGKNLTFSQLIQ-LPDVAP---SL--FDV--GTVPLEVTV------
Diahel2  40 FDS-DNGLFNPDNVFGKNVSFGQLIQ-LPDVDP---SL--FDV--GTVPLEVTI------
Psevex2  40 FDTAANTMFNPDFTFGANLSISDVVE-VPCVTS---SL--FDAKNATKAVEVTI------
Thiter2  41 FDS-PNKFFSEKFVLGQGLKFDQALRVLPVAGG---SL--FDIDVDAQPVEVLI------
Gaetri2  43 FDA-ANNIFNPKNVLGKDLAFSKILQ-LPKTSG---SL--FDN-NGTQPLEVTI------
Magory2  42 FDT-ANNIFNPSNVLGKGLKFSDVIQ-LPTLAQQ--SI--FDVAGKTKAVEVTI------
Maggri2  42 FDS-SNNLFSSSNVLGKGLKFSELIQ-FPANGNQTKTL--FDTAGKTKPLEVTIKYFTPP
Cerpla2  35 FDA-DTSPFNPSNVFGKGLKWSEIIK-FPEGNG---GL--FDV--DTKPIEVTI------
Thipun2  38 FDS-NNGVFNPSFVLGANLTWSEELQ-LPGGS----SL--FSD--NTQPVEVTI------
Colglo2  42 FDT-SASPFNNGFVFGKGLKASDVVT-IPTGLN---SL--FDA-NFSKPFEVNI------
Colnym2  41 FDT-SASPFNNGFVFGKGLKASDVVT-IPTGLS---SL--LDG-PANKPFQVNI------
Stacha2  40 FDT-QNGIYNPDFVKGRDLAFSDIIE-LETDA----TL--FDT--NSVPFTVQI------
Stachl2  40 FDT-QNGIYNPDFVKGRDLAFSDIIE-LETDT----TL--FDT--NSVPFTVQI------
Clapur2  47 FDV-KNNVFNPSFVVGANQTFSSVLR-FDNRGTSH-SL--FDRVGVTKPVEVTI------
```

```
Colhig2   99 ---DDKSIFAPSADNVQIGFRRAEMLPLSNDGKDASTQGIKTLHFSLQKDDKRPLNLSHE
Colchl2   97 ---NDKSIFAPSETNVQVGFRRAELLPLSNDGQDASTDGIKTLHFSLKKDPQRPLNLSHE
Colfio2  100 ---NDKSIFAPSETNVQVGFRRAEMLPLSNDGKDASTQGIKTLHFSLQKDAQRPLNLSHE
Colsim2  100 ---NDKSIFAPSETNVQVGFRRAEMLPLSNDGKDASTQGIKTLHFSLQKDAQRPLNLSHE
Colinc2   99 ---NDKSIFAPSETNVQVGFRRAEMLPLSNDGKDASTQGIKTLHFSLQKDAQRPLNLSHE
Coltof2   99 ---NDKSIFAPSATNVQVGFRRAEMLPLSNDGKDASTQGIKTLHFSLQKDAQRPLNLSHE
Colgra2   83 ---NDKSIFAPSANNVQIGFRRAEMLPASNDGTDPSTDGVKTLHFSLMKDAQRSLNLSHE
Colorb2  102 ---NDKSIFAPSETNVQVGFRRAEMLPLSNDGKDDSTVGVKTLHFSLKKDDQRPLNLSHE
Lompro2   86 ---SDDSIFAPSADNVQTGFRRAELLPASNDGTDPSTTGVKTLHFSLMKDNARPFNLSHE
Sceapi2   85 ---SDDSIFAPSADNIQTGFRRAELLPASNDGTDPSTTGVKTLHFSLMKDNARAFNLSHE
Rosnec2   85 ---SDDSIFAPSADNVQTGFRRAELLIASNDGTDASTTGVKTLHFSIMKDTNRPLNLTHE
Conlig2   86 ---SDKSIFAPSADNVQTGFRRAELLPASNSGTDASTAGVKTLHFSVMKDGARPLNLSHE
Grocla2   84 ---SDESIFAPSASNVQTGFRRAEMLPASNSGTDPSTTGIKTLHFSLMKDSERPLNLSHE
Ophpic2   82 ---SDASIFAPSADNVQTGFRRAELLPASNSGTDASTLGVKTLHFSLMQDPQRPLNLTHE
Spobra2   83 ---SDKSIFAPSADNVQTGFRRAELLPASNSGTDPSTQGIKTLHFSVMQDAQRPLNLTHE
Spoins2   83 ---SDQSIFAPSADNIQTGFRRAELLPASNSGTDPSTQGVKTLHFSVMQDAQRPLNLSHE
Phamin2   90 ---SDQSIFAPSADNVQTGFRRAELLPASNSGTDPSTTGVKTLHFSVMKDADRPLNLSHE
Podans2   84 ---SDASIF------NGQTGFRRAELLPASNSGIDDSTTGIKTLHFSIGKDAQRPLNLSHE
Diaamp2   85 ---SDESIFAPSADNVQLGFRRAELLPASNNGTDASTLGVKSLHFSLMKDAARPLNLSHE
Diahel2   85 ---NDDSIFAPSADNVQLGFRRAELLPASNSGTDPSTQGVKSLHFSLMKDAARPLNLSHE
Psevex2   88 ---DDKSIFTPSADNVQLGFRRAELMITSNDGTDDSTLGVKTTHFSIMKDAARPLNMSHE
Thiter2   89 ---SDNSIF------NGQVGFRRAELIPASNDGTDPSTQGVKTLHFSVKKDAERPLNLSHE
Gaetri2   89 ---DDKSIFVPNADTVQNGFRRVELLAASNSGTDDSTKGVKTLHFSIAKDGQRALNLTHE
Magory2   90 ---NDKSIFVPNADTVQNGFRRTELLIASNNGTDDSTKGVKTLHFSIAKDLKKPLNLTHE
Maggri2   98 GIPSDKSIFVPNPDTVQNGFRRTELLIASNNGTDDSTKGVKTLHFSVAKDAKRPLNLTHE
Cerpla2   80 ---NDTSIFAPSETNVQNGFRRAELLIASNSGTDPSTTGIKTLHFSLKKDAQRPLNTSHE
Thipun2   82 ---NDKSIFAPSPDNVQVGFRRAELQIASNSGTDNSTLGIKTLHFSLKKDITRPLNLSHE
Colglo2   88 ---DDRSIFAPTETNVQTGFRRAEMLPMSNNGTDPSTAGIKTVHWSMMKDPKKPLNLTHE
Colnym2   87 ---DDRSIFAPTETNVQTGFRRAEMLPMSNNGTDPSTSGIKTVHWSMMKDPKKPLNLTHE
Stacha2   84 ---DDSSIFAPSADNVQTGFRRVELLPASNSGTDDSTLGIKTLHFSVAKDVTRPLNLSHE
Stachl2   84 ---DDSSIFAPSADNVQTGFRRVELLPASNSGTDDSTLGIKTLHFSVAKDITRPLNLSHE
Clapur2   96 ---NDRSIFAPSVSNVQVGFRRTELMIASNSGKDISTTGIRTLHFSIAKDPARPLNLDHE

Colhig2  156 YQLAFLESADFSTNQIVLKTGTIL-GQ-N--TEDPDTLQLFGNVATSP-PPELFKTKFTP
Colchl2  154 YQLVFLESADFSTNQVVLKTGTIL-GQ-N--TADPDTLQLFGNVNSSP-VPELFKTKFTE
Colfio2  157 YQLVFLESADFSTNQVVLKTGTIL-GQ-N--TADPDTLQLFGNVNSSP-VPELFKTKFTA
Colsim2  157 YQLVFLESADFSTNQVVLKTGTIL-GQ-N--TADPDTLQLFGNVNSSP-VPELFKTKFTA
Colinc2  156 YQLVFLESADFSTNQVVLKTGTIL-GQ-N--TEDPDTLQLFGNVASSP-TPELFKTKFTP
Coltof2  156 YQLVFLESADFSTNQVVLKTGTIL-GQ-N--TADPDTLQLFGNVASSP-TPELFKTKFTP
Colgra2  140 YQLVFLESADFSTNQVVLKTGSIL-GQ-N--TADPDTLQLFGNVNSNP-VPELFQTKFTP
Colorb2  159 YQLVFLESADYSTNQIVLKTGSLI-GE-N--TADPDTLQLFGNVASSP-VPQLFKTKFTP
Lompro2  143 YQLVFLESNDFSTNQIVLKTGTIL-GV-N--TADPDTLQLFGNVNQG---NLLFSTPFTA
Sceapi2  142 YQLVFLESADFSTNQFVLKTGTIL-GG-N--TADPDTLQLFGNVNQG---QLLFSTPFTA
Rosnec2  142 YQLVFLESNDFSTNQIVLKTGSIL-GA-N--TADPDTLQLFGNVNANP-VQTLFSVPFTE
Conlig2  143 YQLVFLESNDFSTNQLVLKTGTVI-GA-G--AGDPDTLQLFGNVNESP-PKVLFSTPFQA
Grocla2  141 YQLVFLESSDFSTNQLVLKTGTLI-GQ-E--TANPDTLQLLGNVNVNP-PQVLFSTPFLP
Ophpic2  139 YQLVFLESADFSTNQLVLKTGTII-GSTD--GVDPNTLQLFGNVNANP-PQVLFSTPFTP
Spobra2  140 YQLVFLESNDFSTNQLVLKAGTVL-GQAG--NVDPNTLQLFGNVNSNP-PQVLFSTPFTP
Spoins2  140 YQLVFLESNDFSTNQLVLKTGTIL-GGAA--GVDPNTLQLFGNVNANP-PQVLFSTPFTP
Phamin2  147 YQLVFLESNDFSTNQLVLKTGTIL-GQ-A--TADPDTLQLIGNVNDG--ANVLFSTPFVD
Podans2  136 YQLVFLESNDFSTNQFVLKTGTIL-GGDT--AVDPDTLQLFGNVNQTP-SKLLFSVPFLD
Diaamp2  142 YQLVFVESSDFSTNQFVLKTGTLL-DTATQGGVDPDSLILFGNVKQS---QILFNTAFTE
Diahel2  142 YQLVFVESSDFSTNQFVLKTGQIL-DTATQKGVDPDSLILFGNVKQS---EQLFSTPFTE
Psevex2  145 YQLFFLESADFSTNQVVLKTGSII-GA-D--TPNPDTLQLFGNVNSSP-LQTLFSTNFTE
Thiter2  141 YQLVFLESNDFSTNQFVLKTGTIL-GQ-N--TADPDTLQVFGNVNQG---QLLFSTPFVD
Gaetri2  146 YQLVFLEDNSFSTNQVVLKTGTVA-GQ-N--GGNPDTLQLFGNVKESP-PKVLFSTPFAA
Magory2  147 YQLVFLEDNKFSTNQIVVKTGTID-GQ-A--NTNPDTLNVFGNVASSP-PKLLFSTPFGA
Maggri2  158 YQLVFLEDNKFSTNQIVVKTGTIA-GQ-T--SNNTNTLNVFGNVNASP-PKLLFSTPFAA
Cerpla2  137 YQLVFLEDSTYSTNQFVLKTGTVLTGD-G--ASDPDTLQLFGNVNQKE-PGMIFSAPFTP
Thipun2  139 YQLVFLEDNTFSTNQFALKTGTIL-GQ-N--TTNPDTLQLFGNVNDAK-PGLIFSAPFTA
Colglo2  145 YQMVFLESSIFSSNQYALKYGDLI-GI-H--PADPDVLVLFGNSNTKPFQPELFRTKFTD
Colnym2  144 YQMVFLESSIFSSNQFALKYGDLI-GI-H--PADPDVLHLFGNSNTNP-SPELFKTKFTE
Stacha2  141 YQLVFLESADYSNSQFDIKTGTIL-GR-D--TPDPDTIQVYGNINAG--AALLYSTPFTE
Stachl2  141 YQLVFLESADYSNSQFDIKTGTIL-GR-D--TPDPDTIQVYGNINAG--AALLYSTPFTE
Clapur2  153 YQLSFLEDSSFSTNQFVLKTGNIT-GV-N--TLDPDSLTLFGNTNKF--GDVLFTTPFTA
```

```
Colhig2  211  GVFHNFAIKLDFTANTTEVFYSQGTSALKSQGAAVPNIIAGQGQYHFGILKKPVNGGADI
Colchl2  209  GVFHNFAIKLDFTKNTTEVFYSQGNSALKSQGAPVSNNIAGKGQYHFGMLKKPVNGGSDI
Colfio2  212  GVFHNFAIKLDFTKNTTEVFYSQGNSALKSTGAAVTNNIAGQGQYHFGMLKKPVNGGSDI
Colsim2  212  GVFHNFAIKLDFTKNTTEVFYSQGNSALKSTGAAVANNIAGQGQYHFGMLKKPVNGGSDI
Colinc2  211  GVFHNFAIKLDFTANTTEVFYSQGNSALKSQGAAVANNIAGQGQYHFGMLKKPVNGGADI
Coltof2  211  GVFHNFAIKLDFTANTTEVFYSQGNSALKSQGAPVANNIAGQGQYHFGILKKPVNGGDDI
Colgra2  195  GVFHNFAVKLDFTANTTQVFYSQGNSALKSQGAPVANNIAGKGQFHFGVLKKPVNGGADI
Colorb2  214  GVFHNFAVKLDFTKNTTEVFYSTDNSALKSQGAAVANNIAGQGQYHFGVLKKPVNAGSDM
Lompro2  196  GVFHNFGITLDFDALTTQVFYSTGDDPLEQQTDAIANDVSGQGQFHFGVLKKPTDSAGDI
Sceapi2  195  GVFHNFGVVLDFDALTTQVLYSTGDDPLEAQTDAIDNDVSGEGQFHFGVLKKPTDSAGDI
Rosnec2  197  DVFHNFAVTLDFDALTTQVFYSQGVDALVAQTEALANDVSGQGQFHFGVLKKGLNGGDDI
Conlig2  198  GVFHNFGVMLDFDKLTSQVFFSTGTTALKAVTQALANDVSGQGQFHFGVLKKGI-GGNNI
Grocla2  196  DVFHNFAVTMDFNNLTTQVFFSTGNSSLKAVTQALSNDISGQGEFHFGMLKKPIDGGSDI
Ophpic2  195  GVFNNFAVTLDFNNLTSQVFFSTGKSPLKPVTQAISNDVSGQGQFHFGMLKKPIDGGNDI
Spobra2  196  GVFHNFAVTLDFNKLTSQVFFSTGTTALKSVTQALANDVSGQGQFHFGVLKKPVNPGADI
Spoins2  196  GVFQNFAVTLDFDKLTSQVFFSTGTSPLKAQTQALSNDVSGQGQFHFGVLKKGLDGGDDI
Phamin2  201  GAFHNFGVTLDFNNLTTQVFFSAGTDALEAVTQPIANDVSGQGQFHFGVLKKGLNGGDDI
Podans2  192  GVFHNFAVTLDFDTLTTQVFFSAGGDPLEVVNQVISNDVSGQGQFHFGLLKKPTDAVGDI
Diaamp2  198  GVFHNFGLTLDFDANTTQVFYSTGNDPLAQVTEPLPNDISGQGQYHFGVLKKGVNGGDDI
Diahel2  198  GVFHNFGLTLDFNANTTQVFYSTGNEPLAQVTDALPNDISGQGQYHFGVLKKGVNGGSDI
Psevex2  200  GVFHNFAVTLDFDKNTTQVFYSQDSSALEAQTEPLANDVSGQGQYHFGVLKKGIGGDVDI
Thiter2  194  GVFHNFALKLDFDQSTTQVFFSAGEDPLKAVTGVLENDLSGQGQFHFGVLKKPVGGQGDI
Gaetri2  201  NGFHNFGITMDFNKNTVQVLFSTGAQPLKAVTQPIANDLSGGGQYHFGMLKKGLGGGADI
Magory2  202  DGFHNFAMTLDFTKNTTQVFFSKGNEPLKAVSKAEPNDVSGGGQFHFGCLKKGVGKVKDV
Maggri2  213  EGFHNFAIKMDFDKNTTQVFFSKGSEALKAVSKEESNDVSGGGQFHFGILKKGVGNAKDI
Cerpla2  193  GTFHNFGLKLDFTANTTQVFYSTGNCELTQQTEPINNDVSGQGQFHFGILKKPTGETTDV
Thipun2  194  GKFHNFALKLDFNANTTQVFYSTGNCSLSQQTEAIANDVSGQGQFHFGILKKPTGNFTDI
Colglo2  201  GVFHNFALTLDFTKNMTQIYYSQGSAPLVAQGKPVENDISGQGQFHFGVLKKSINGTGDT
Colnym2  199  GVFHNFALTLDFGKNLTQVYYSQGNAPLVAQGQPIENDISGQGQFHFGVLKKSINGTGDL
Stacha2  195  GVIHNFALRLDYETGTTQIFYSQDQAQLQSVTDILANDLSGQGQYHIGLLKKPTGSDGDI
Stachl2  195  GVIHNFALRLDYETGTTQIFYSQDQAQLQSVTDILANDLSGQGQYHIGLLKKPTGSDGDI
Clapur2  207  GTFHNFALRLDFTRNTTEVRYSTGLHALKSVTGPRANDISGQGQYHFGLLKKGLNGNGD-

Colhig2  271  TKSGDQPADIDEGLIYAGIFQEDSANGCISLSP
Colchl2  269  TKSGDQPSGINEGIIYGGIFQEDSSSGCISLSP
Colfio2  272  TKSGDQPTGINEGIIYGGIFQEDSSAGCISLSP
Colsim2  272  TKSGDQPTGINEGIIYGGIFQEDSSAGCISLSP
Colinc2  271  TKSGDQPSGINEGIIYAGIFQEDSANGCISLSP
Coltof2  271  TKSGDQPSGINEGIVYAGIFQEDSSTGCISLSP
Colgra2  255  SKSGEQPANINEGVIYGGIFEEDSSTGCISLSP
Colorb2  274  TKSGDQPANIDEGIIYGGIFQEDSSSGCISLSP
Lompro2  256  TKDGFQEAGIDEGLIYAGIFQEDSSDGCISLSL
Sceapi2  255  TKDGFQEAGIDEGLIYAGIFQEDSSDGCISLSL
Rosnec2  257  VKNGEQEPGIDEGIIFGGIFEEDSSDGCITLSV
Conlig2  257  VKDGVQEAGIDEGIIFGGIFEEDSSAGCVSLSP
Grocla2  256  VHNGTQESDIDEGIIYGGIFEEDSSTGCVSLSP
Ophpic2  255  VHNGTQEDGIDEGIIYGGIFEEDSSTGCISLCA
Spobra2  256  VHSGTQEPGINEGVIFGGIFEEDSSTGCISLSP
Spoins2  256  VHDGIQEPGINEGIIFGGVFEEDSSTGCVSLSP
Phamin2  261  VKNGEQEAGINEGIIFGGIFEEDSSTGCISLSP
Podans2  252  TRNGFQEDGIDEGIIFGGIFQEDSSAGCVSLRP
Diaamp2  258  TKDGQQPAGIDEGIIYGGIFQEDSAASSITLSP(40)
Diahel2  258  TRDAQQPSGISEGIIYGGIFLEDSSASTITLSP(34)
Psevex2  260  TRLAFQPAGIDEGIIYGGIFQEDSADGCISLSA
Thiter2  254  TKNGFQEAGINEGIILGGIFEEDSAVGCITLAG(3)
Gaetri2  261  VRNGVQPAGINEGLIYGGIFMEDSSTGCVSLAP(16)
Magory2  262  VREGIQEPNINEGVIYGGIFEEDSANGCISLSA(56)
Maggri2  273  IRDGIQEANINEGLIYSGIFMEDSADGCVSLST(22)
Cerpla2  253  TKSGFQPADIDEGIIYSGIFSEDSADGCVSLSP(4)
Thipun2  254  TKSGFQESGINEGIVYSGIFMEDSADDCLSLSR(4)
Colglo2  261  TKTGFHESGINEGIIFGGIFEEDSADGCVSLSP
Colnym2  259  TKSGFHESGINEGIIFGSIFEEDSIDGCVTLSL
Stacha2  255  TREGFQSSGINEAVLYGGIFLEDSADGCLSLGP(15)
```

```
Stachl2  255 TREGFQSSGINEAVLYGGIFLEDSADGCLSLGP(15
Clapur2  266 SKKGTQETGINEGIIYGGIFMEDSSENCRCNY-
```

<u>Sordariomycetes</u>
Hypocreomycetidae - Glomerellales (Colletotrichum but not Verticillium)
Colchl2 = OLN97937.1 hypothetical protein CCHL11_02648 [Colletotrichum chlorophyti]
Colfio2 = EXF80808.1 hypothetical protein CFIO01_01179 [Colletotrichum fioriniae PJ7]
Colglo2 = KAF3811743.1 hypothetical protein GCG54_00014494 [Colletotrichum gloeosporioides]
Colgra2 = XP_008098621.1 hypothetical protein GLRG_09745 [Colletotrichum graminicola M1.001]
Colhig2 = TID06827.1 hypothetical protein CH35J_000821 [Colletotrichum higginsianum], which is equivalent to CH063_00647 described by Anasontzis et al. (2019) except the signal peptide sequence is correct
Colinc2 = KZL82458.1 hypothetical protein CI238_03137, partial [Colletotrichum incanum]
Colnym2 = KXH25668.1 hypothetical protein CNYM01_07213 [Colletotrichum nymphaeae SA-01]
Colorb2 = TDZ16663.1 hypothetical protein Cob_v010277 [Colletotrichum orbiculare MAFF 240422]
Colsim2 = KXH47399.1 hypothetical protein CSIM01_05336 [Colletotrichum simmondsii]
Coltof2 = KZL71646.1 hypothetical protein CT0861_07364 [Colletotrichum tofieldiae]
Hypocreomycetidae - Hypocreales - Clavicipitaceae (in Claviceps purpurea and other Claviceps species but not Metarhizium, Moelleriella libera, Pochonia chlamydosporia or Torrubiella hemipterigena)
Clapur2 = KAG6202730.1 hypothetical protein E4U50_006098 [Claviceps purpurea]
Hypocreomycetidae - Hypocreales - Cordycipitaceae - no orthologues found
Hypocreomycetidae - Hypocreales - Hypocreaceae - no orthologues found
Hypocreomycetidae - Hypocreales - Nectriaceae - no orthologues found
Hypocreomycetidae - Hypocreales - Ophiocordycipitaceae - no orthologues found
Hypocreomycetidae - Hypocreales - Stachybotryaceae
Stacha2 = KFA47799.1 hypothetical protein S40293_09048 [Stachybotrys chartarum IBT 40293]
Stachl2 = KFA69030.1 hypothetical protein S40285_08453 [Stachybotrys chlorohalonata IBT 40285]
Hypocreomycetidae - Microascales
Cerpla2 = KKF95434.1 hypothetical protein CFO_g2225 [Ceratocystis platani]
Thipun2 = KKA28951.1 hypothetical protein TD95_005422 [Thielaviopsis punctulata]
Lompro2 = PKS05998.1 hypothetical protein jhhlp_007831 [Lomentospora prolificans]
Sceapi2 = XP_016645553.1 hypothetical protein SAPIO_CDS1553 [Scedosporium apiospermum]
Sordariomycetidae - Coniochaetales
Conlig2 = OIW32631.1 hypothetical protein CONLIGDRAFT_699488 [Coniochaeta ligniaria NRRL 30616]
Sordariomycetidae - Togniniales
Phamin2 = XP_007911498.1 hypothetical protein UCRPA7_713 [Phaeoacremonium minimum UCRPA7]
Sordariomycetidae - Diaporthales
Coniella lustricola - no orthologue found
Diaamp2 = KKY30855.1 hypothetical protein UCDDA912_g09201 [Diaporthe ampelina]
Diahel2 = POS74227.1 hypothetical protein DHEL01_v207386 [Diaporthe helianthi]
Sordariomycetidae - Magnaporthales
Gaetri2 = XP_009219288.1 hypothetical protein GGTG_03245 [Gaeumannomyces tritici R3-111a-1]
Magory2 = QBZ65172.1 hypothetical protein PoMZ_06877 [Pyricularia oryzae] (syn. Magnaporthe oryzae)
Maggri2 = XP_030976736.1 hypothetical protein PgNI_11158 [Pyricularia grisea]
Sordariomycetidae - Ophiostomataceae
Grocla2 = XP_014171689.1 hypothetical protein CMQ_2256 [Grosmannia clavigera kw1407]
Ophpic2 = EPE10934.1 hypothetical protein F503_06029 [Ophiostoma piceae UAMH 11346]
Spobra2 = XP_040620669.1 uncharacterized protein SPBR_02939 [Sporothrix brasiliensis 5110]
Spoins2 = OAA67092.1 hypothetical protein SPI_01668 [Sporothrix insectorum RCEF 264]
Sposch2 = XP_016591024.1 hypothetical protein SPSK_07672 [Sporothrix schenckii 1099-18]
Sordariomycetidae - Sordariales - Sordariales incertae sedis
Madurella mycetomatis - no orthologue found
Sordariomycetidae - Sordariales - Chaetomiaceae
Chaetomium thermophilum var. thermophilum - no orthologue found
Podans2 = XP_001908978.1 uncharacterized protein PODANS_3_2140 [Podospora anserina S mat+] corrected for upstream methionine start and unrecognised intron
Thermothelomyces thermophilus - no orthologue found
Thiter2 = SPQ19874.1 44879d5e-d898-4d54-8381-2fd604ce673d [Thermothielavioides terrestris](syn. Thielavia terrestris)with corrected N-terminal sequence in place of erroneous starting methionine
Sordariomycetidae - Sordariales - Sordariaceae - no orthologues found
Xylariomycetidae - Xylariales
Eutypa lata - no orthologue found

Daldinia sp. - no orthologue found
Hypoxylon sp. - no orthologue found
Microdochium bolleyi - no orthologue found
Pestalotiopsis fici - no orthologue found
Psevex2 = XP_040720469.1 uncharacterized protein BCR38DRAFT_334322 [Pseudomassariella vexata]
Rosnec2 = GAP88082.1 hypothetical protein SAMD00023353_3201390 [Rosellinia necatrix]

Supplementary Figure S7. ChGluc131A alignment. Multiple protein-sequence alignment of ChGluc131A orthologues in the Fol-EC4 clade of Sordariomycete GH131 (glycosyl-hydrolase 131) sequences. All sequences shown were the highest blastP matches to ChGluc131A (= Colhig2 highlighted in green in the alignment) obtained for each genome-sequenced species represented. Sequences were aligned using ClustalW at https://www.ebi.ac.uk/Tools/msa/mafft/. Amino-acid identities were highlighted in black and similarities in grey using BoxShade at https://embnet.vital-it.ch/software/BOX_form.html. Conserved cysteines have been highlighted manually in blue, residues conserved with the catalytic residues of PaGluc131A and CcGH131A in red and non-conserved residues in teal. The number of C-terminal residues removed manually for the purposes of alignment are shown in brackets at the end of the alignment.
