# Supplementary material for: Transcriptome Analysis of Fusarium–Tomato Interaction Based on an Updated Genome Annotation of Fusarium oxysporum f. sp. lycopersici Identifies Novel Effector Candidates That Suppress or Induce Cell Death in Nicotiana benthamiana
Source: J Fungi (Basel). 2022 Jun 26;8(7):672. doi: 10.3390/jof8070672 (PMC9316272; doi:10.3390/jof8070672)
Supplement: Supplementary file 1 [file jof-08-00672-s001.zip › Supplementary Materials/Supplementary Figures/Supplementary Figure S8.pdf]

```
Colhig3    1 MILPHAAVRSLQALSLNAARQPLQSSSIQCPIVLDGRIPQNFTLQVFDTNESPFNPGFTK
Colinc3    1 MMVPHMAVRSLQALSLNSALQSLQ-SSVQCPIVLDGRIPQNFSLQVFDTNESPFNPGFTK
Coltof3    1 MVLPHAAVRSLQALSLNSALQSLQ-LPVQCPIVLDGRIPQNFSLQVFDTNDSPFNPGFTK
Colfio3    1 MILPHAAVRSLQALSLNAPRASFD-SPVKCPVVLDGRIPQNFTLQVFDTYASPFNPGYTK
Colnym3    1 MILPHAAVRSLQALSLNAPRASLD-SPVKCPVVLDGRIPQNFTLQVFDTYASPFNPGYTK
Colsim3    1 MILPHAAVRSLQALSLNAPRASLD-SPIKCPVVLDGRIPQNFTLQVFDTYDSPFNPGYTK
Colsal3    1 MILPHAAVRSLQALALNVPRASLD-SPVKCPVVLDGRIPQNFTMEVFDTSTSPFNPGFTK
Colchl3    1 MILPHTAVRSLQALSLNAPFQSFQ-SPVRCPVVLDGRIPQNFTLEIFDTNASPFNPGFTK
Colglo3    1 MILPHLAVRAL-S------------ASVQCPVVLDGRISQNFTLENFDTNVSPYNPGFTK
Colorb3    1 MILPQVAARALLGLS----------SAVKCPIIHDGRIPQNFTLDDFDTNASPYNPGFTK

Colhig3   61 GPAPWSEILLFPAGVTPSRFDVVSEP---TATSFKPLEVTIDDKSIFD-PSGNNPQLGFR
Colinc3   60 GPAPWSDILLFPTNTTPSRFDVSN-----STGNFKPVEVTINDQSIFD-PSGNNPQVGFR
Coltof3   60 GPAPWSEILLFPAITTPSRFDVSS-----STGDSKPVEVTINDQSIFD-PSGNNPQIGFR
Colfio3   60 GPAKWSEILLFPTVPTSSRFDASN-----ATGSFKPLEVTINDQSIFN-PSGNSPQLGFR
Colnym3   60 GPAKWSEILLFPTVPTPSRFDTSN-----ATGNFKPLEVTINDQSIFN-PSGNSPQLGFR
Colsim3   60 GPAKWSEILLFPTVPTPSRFDTSN-----ATGNFKPLEVTINDQSIFN-PSGNSPQLGFR
Colsal3   60 GPAPWSEILLFPTVPTPSRFDASN-----ATGNFKPLEVTINDQSIFN-PSGNSPQLGFR
Colchl3   60 GPALWSEILLFPNISTTSRFDVSS-----QTGDFKPLEVTINDQSIFNPPPGNNPQLGFR
Colglo3   48 GSDPWSKIILFPTVAAPSRFDAAASANATSGSGFKPLEVTINDRSIFNPPPGKNPQLGFR
Colorb3   51 GPDPWSKILLFPPVAQTSRFDVAGR----SPANTKPLEVTINDDSIFNPPPGKNPQLGFR

Colhig3  117 RAGLLLGNGSDASNQGVMTFHWSSRQDPARKLNLTHEYLTSFHETNDSGGAQFSLQVGTP
Colinc3  114 RAGLLLGNGSDASNQGVMTFHWSSMQDPTRKLNLTHEYLVSFHETNDSGGAQFALQIGTP
Coltof3  114 RAGLLLGNGSDASNQGVKTFHWSSRQDSTRKLNLTHEYLTSFHETNDSGGAQFALQIGTP
Colfio3  114 RAGLLLGNGSDASNQGVMTTHWSSMQDSTRKLNLTHEYLTSFHETNDSGGAQFALQIGTP
Colnym3  114 RAGLLLGNGSDASNQGVMTTHWSSMQDSTRKLNLTHEYLTSFHETNDSGGAQFALQIGTP
Colsim3  114 RAGLLLGNGSDASNQGVMTTHWSSMQDSTRKLNLTHEYLTSFHETNDSGGAQFALQIGTP
Colsal3  114 RAGLLLGNGSDASNQGVMTTHWSSMQDSTRKLNLTHEYLTSFHETNDSGGAQFALQIGTP
Colchl3  115 RAGLLLGNGSDASNEGVMTFHWSSRQDSARKLNLTHEYLTAFHETNDSGGAQFALQLGTP
Colglo3  108 RAGLLLGNGSDASNQGVMTFHWSAMQDSARKLNLTHEYLTSFHETNDSGGAQFALQVGLP
Colorb3  107 RAGLLLGNGSDAANEGAVTIHWSSRQDPARRLNLTHEYATAFHETNDSGGAQFTLQLGLP

Colhig3  177 LGQDGAAGKPAASDWKMLNRNNTVVFSTPLKFDAWQNFAVTLDVPKNTMQVYFSEGNDPL
Colinc3  174 LGQNGTAGRPSPTDWKMLDRNNTVVFSTPLKFDAWQNFAVTLDVPKNTMQVFFSEGNDPL
Coltof3  174 LGQNSTAGRPSLSDWKMLDRNNTVVFSTPLKFDAWQNFAVTLDVPKNTMQVFFSEGNEPL
Colfio3  174 LGQNSTAGKPLSNDWKMLDRNNSVIFSTALKFDVWQNWAVTLDVPKNTIQVFFSEGNDPL
Colnym3  174 LGQNSTAGKPLSTDWKMLDRNNSVIFSTALKFDVWQNWAVTLDVPKNTIQVFFSEGNDPL
Colsim3  174 LGQNSTVGKPLSTDWKMLDRNNSVIFSTALKFDVWQNWAVTLDVPKNTIQVFFSEENDPL
Colsal3  174 LGQNSTTGGPSSADWKMLDRNNSVIFSTALKFDVWQNWAVTLDVPKNTIQVFFSEGNDPL
Colchl3  175 LGQNSTLSGPASSDWKMLDRDNKVVFSTPLKFDAWQNFGVTLDIPNNTLQVYFSEGDDPL
Colglo3  168 LGQNGT--EPPKENWKMLDRNNSVVFTTPLKFDTWQNWAVTLDVPQNTMQVFFSEGNEPL
Colorb3  167 LGQNKT--EPPAANWKMLNRNNSLVFTTPLQFGKWQNFAVTLDVPKNTMQVYYSEGNEPL

Colhig3  237 KPVTEILPNDNSGGGALQIGLLKKPTETKTVVFDGFQESNIDEGQILGSIFVEDSANGCV
Colinc3  234 KPVTEVLPNDNSGGGALQIGILKKPTETKTVVFDGFQESNLNEGQILGGIFVENSANGCI
Coltof3  234 KPVTEVFPNDNSGGGVLQIGILKKPTETKTAVFDGFQESNLNEGQILGGIFVEDSANGCV
Colfio3  234 KSVTEVLPNNNAGGGALQIGLLKKPTETKTVVNDGFQESNLNEGQILGGIFVENSANGCV
Colnym3  234 KSVTEVLPNNNVGGGALQIGLLKKPTETKTVVNDGFQETNLNEGQILGGIFVENSANGCV
Colsim3  234 KSVTEVLPNNNVGGGALQIGLLKKPTETKTVVNDGFQESNLNEGQILGGIFVENSANGCV
Colsal3  234 KSVTEVLPNNNAGGGALQIGLLKKPTETKTVVNDGFQESNLNEGQILGGIFVENSANGCI
Colchl3  235 KPVTEVLPNNNAGGGALQIGMLKKPTETTTAVFDGFQENNLNEGQILGGIFVENSANGCV
Colglo3  226 KAVTKILPNNNAGGGALQIGMLKKPTETKTVANDGFQESNLNEGQILGGIFVENSANGCI
Colorb3  225 SAVTGVLPNNNAGGGALQIGILKKPTETKTVVFDGFQESNVNEGQILGGIFIENSSDGCV

Colhig3  297 SL
Colinc3  294 SL
Coltof3  294 SL
Colfio3  294 SL
Colnym3  294 SL
Colsim3  294 SL
```

```
Colsal3  294 SL
Colchl3  295 SL
Colglo3  286 SL
Colorb3  285 SL
```

<u>Sordariomycetes</u>
Hypocreomycetidae - Glomerellales (Colletotrichum but not Verticillium)
Colchl3 = OLN81946.1 hypothetical protein CCHL11_08641 [Colletotrichum chlorophyti]
Colfio3 = EXF72940.1 hypothetical protein CFIO01_04646 [Colletotrichum fioriniae PJ7]
Colglo3 = KAF3801482.1 hypothetical protein GCG54_00014696 [Colletotrichum gloeosporioides]
Colletotrichum graminicola - no orthologue
Colhig3 = CCF43865.1 hypothetical protein CH063_03177 [Colletotrichum higginsianum] which is equivalent to CH063_00647 described by Anasontzis et al. (2019)
Colinc3 = KZL79966.1 hypothetical protein CI238_09667 [Colletotrichum incanum]
Colnym3 = KXH53449.1 hypothetical protein CNYM01_01194 [Colletotrichum nymphaeae SA-01]
Colorb3 = TDZ24818.1 hypothetical protein Cob_v002377 [Colletotrichum orbiculare MAFF 240422]
Colsal3 = KXH68549.1 hypothetical protein CSAL01_02206 [Colletotrichum salicis]
Colsim3 = KXH27864.1 hypothetical protein CSIM01_06783 [Colletotrichum simmondsii]
Coltof3 = KZL72700.1 hypothetical protein CT0861_06503 [Colletotrichum tofieldiae]
Hypocreomycetidae - Hypocreales - Clavicipitaceae -no orthologues
Hypocreomycetidae - Hypocreales - Cordycipitaceae - no orthologues
Hypocreomycetidae - Hypocreales - Hypocreaceae - no orthologues
Hypocreomycetidae - Hypocreales - Nectriaceae - no orthologues
Hypocreomycetidae - Hypocreales - Ophiocordycipitaceae - no orthologues
Hypocreomycetidae - Hypocreales - Stachybotryaceae - no orthologues
Hypocreomycetidae - Microascales - no orthologues
Sordariomycetidae - Coniochaetales - no orthologues
Sordariomycetidae - Togniniales - no orthologues
Sordariomycetidae - Diaporthales - no orthologues
Sordariomycetidae - Magnaporthales - no orthologues
Sordariomycetidae - Ophiostomataceae - no orthologues
Sordariomycetidae - Sordariales - Sordariales incertae sedis - no orthologues
Sordariomycetidae - Sordariales - Chaetomiaceae - no orthologues
Sordariomycetidae - Sordariales - Sordariaceae - no orthologues
Xylariomycetidae - Xylariales - no orthologues

Supplementary Figure S8. ChGluc131B alignment. Multiple protein-sequence alignment of ChGluc131B orthologues in the Fol25 clade of Sordariomycete GH131 (glycosyl-hydrolase 131) sequences. All sequences shown were the highest blastP matches to ChGluc131B (= Colhig3 highlighted in green in the alignment) obtained for each genome-sequenced species represented. Sequences were aligned using ClustalW at https://www.ebi.ac.uk/Tools/msa/mafft/. Amino-acid identities were highlighted in black and similarities in grey using BoxShade at https://embnet.vital-it.ch/software/BOX_form.html. Conserved cysteines have been highlighted manually in blue, residues conserved with the catalytic residues of PaGluc131A and CcGH131A in red and non-conserved residues in teal. None of these sequences were found to have predicted signal peptide sequences when analysed using SignalP 5.0 (http://www.cbs.dtu.dk/services/SignalP/) or SignalP 4.1 (http://www.cbs.dtu.dk/services/SignalP-4.1/) but Colorb3 and Colsal3 had predicted signal peptide sequences when analysed using SignalP 3.0 (http://www.cbs.dtu.dk/services/SignalP-3.0/) and all were predicted to be secreted proteins when analysed using Secretome P 2.0 (http://www.cbs.dtu.dk/services/SecretomeP/).
