# Supplementary material for: Transcriptome Analysis of Fusarium–Tomato Interaction Based on an Updated Genome Annotation of Fusarium oxysporum f. sp. lycopersici Identifies Novel Effector Candidates That Suppress or Induce Cell Death in Nicotiana benthamiana
Source: J Fungi (Basel). 2022 Jun 26;8(7):672. doi: 10.3390/jof8070672 (PMC9316272; doi:10.3390/jof8070672)
Supplement: Supplementary file 1 [file jof-08-00672-s001.zip › Supplementary Materials/Supplementary Tables/Supplementary tables S1 S2 and S4.docx]

**Table S1 Primers used in this study.**

| **Primer name** | **Sequence** |
| --- | --- |
| ACTIN-qPCR-F | TCACCACCTTCAACTCCATCA |
| ACTIN-qPCR-R | AACGATTGAGGGACCGCTCT |
| FOXG_11745-qPCR-F | ACACCGTGACGGATCAACTC |
| FOXG_11745-qPCR-R | GCAGCCATCAGAAGTCCAGT |
| FOXGR_021626-qPCR-F | ACCTATCGCTTCAAGGGGAA |
| FOXGR_021626-qPCR-R | AGATGTCGATGGCGGTCATA |
| FOXG_04805-qPCR-F | CAAAATGGTCAGAACGGCGG |
| FOXG_04805-qPCR-R | TGTTACCACCGGGAACACAG |
| FOXG_18699-qPCR-F | GCTACTCGTGCACTCAGGTT |
| FOXG_18699-qPCR-R | TAGTTCCGACAGCAGAGCAC |
| FOXG_08899-qPCR-F | CAAGCCCACTGCCAGGTAAT |
| FOXG_08899-qPCR-R | TCCAAGATGTACTTCGCACCA |
| PSL1-qPCR-F | CCTGCGTTCTGCCCAACTAT |
| PSL1-qPCR-R | TAACCAACGGACCAGACGTG |
| SIX9-qPCR-F | TTCTTAACCCATCTGCGCGT |
| SIX9-qPCR-R | ACTTGTTGCAGCACTTTCGC |
| FOXGR_015533-qPCR-F | GCTAGACCCTCAGCTGGAAC |
| FOXGR_015533-qPCR-R | GGTCCCGCGATACGGTAAAT |
| FOXG_10949-qPCR-F | GTACTCTGACGGAGGCGTTT |
| FOXG_10949-qPCR-R | TTTTGGGCGCAGCAAACAAT |
| FOXG_10950-qPCR-F | TCGTCGAATGACCAGACAGC |
| FOXG_10950-qPCR-R | ATTGATGGTGACGGGCATGT |
| Fol-EC1-F | ATCTTCTCACTCTACGCTGCGCCGAGGATGCAAA |
| Fol-EC1-R | TCCTCTCCAAATTACGTTAGTTCCAGCTGCAGGGA |
| Fol-EC2-F | ATCTTCTCACTCTACGGAAGACTGGGACCAGTGCCGAT |
| Fol-EC2-R | TCCTCTCCAAATTACGTTACTTGAAGCATCCATCCGATGC |
| Fol-EC3-F | ATCTTCTCACTCTACGTCTGCTCAGACCTGTGCAATCG |
| Fol-EC3-R | TCCTCTCCAAATTACGTTATTCTTTATACCAAGCAGCATTTGTC |
| Fol-EC4-F | ATCTTCTCACTCTACGGAAGACTGGGATCGGTGCCG |
| Fol-EC4-R | TCCTCTCCAAATTACGCTACTTGAAACAGCCATCTGACTGC |
| Fol-EC5-F | ATCTTCTCACTCTACGGCTCCTGGTAACCGTGGTGG |
| Fol-EC5-R | TCCTCTCCAAATTACGTTAGACATAGATAACCTTCTGGTTGCC |
| Fol-EC6-F | ATCTTCTCACTCTACGTCTCCGGTTGGTAACGTAGCTG |
| Fol-EC6-R | TCCTCTCCAAATTACGCTACTTCCCGCCCTTCTTGCC |
| Fol-EC7-F | ATCTTCTCACTCTACGGCTCCGGAAGCAGCACCA |
| Fol-EC7-R | TCCTCTCCAAATTACGTTAGAAGAGAGAGTTGATGAACTCCTTG |
| Fol-EC8-F | ATCTTCTCACTCTACGAAACCCTACGTGGTTACCG |
| Fol-EC8-R | TCCTCTCCAAATTACGCTAACCCTCAATAGGTGTAGAAATG |
| Fol-EC9-F | ATCTTCTCACTCTACGTGCAAGAGAACCTGCTCTGCC |
| Fol-EC9-R | TCCTCTCCAAATTACGCTAGCAGCCACTGCCGCAT |
| Fol-EC10-F | ATCTTCTCACTCTACGTCACTTGTCCGCCGAGTCGATG |
| Fol-EC10-R | TCCTCTCCAAATTACGTTATTGCTTCTGCTGCTGCTTCTG |
| Fol-EC11-F | ATCTTCTCACTCTACGCTTCCCACCGAGGTTAAGC |
| Fol-EC11-R | TCCTCTCCAAATTACGTTAGCTCTGGTACAGACAGTTGA |
| Fol-EC12-F | ATCTTCTCACTCTACGGGCATCGTCGACACTGAGGG |
| Fol-EC12-R | TCCTCTCCAAATTACGTTAGTTGACCTTAGCCTGGCTAAGG |
| Fol-EC13-F | ATCTTCTCACTCTACGGCGCCCTCCTCCCCAAGCG |
| Fol-EC13-R | TCCTCTCCAAATTACGTTACTCCTCACCCATAAGGTGCGAC |
| Fol-EC14-F | ATCTTCTCACTCTACGACTCCCTTCGGCCAACGC |
| Fol-EC14-R | TCCTCTCCAAATTACGTTAGAGAGAAACACATCCATCTGCG |
| Fol-EC15-F | ATCTTCTCACTCTACGAGCCCACTGCCAGGTAATGC |
| Fol-EC15-R | TCCTCTCCAAATTACGTCAAGCCCCACTTTCAGGCTC |
| Fol-EC16-F | ATCTTCTCACTCTACGCTCCCTACAGGCGAAGATGCTG |
| Fol-EC16-R | TCCTCTCCAAATTACGTTAGTCAAGACGAGGCAGCTCG |
| Fol-EC17-F | ATCTTCTCACTCTACGGCTCCCGTGGTCCGTGGCC |
| Fol-EC17-R | TCCTCTCCAAATTACGTTATTTTGACGAGAACGGATCATCTTCG |
| Fol-EC18-F | ATCTTCTCACTCTACGAGCCCCATCAGCAAGCGC |
| Fol-EC18-R | TCCTCTCCAAATTACGTTAGCTTGTAGTCGCATCAAAAGCAAG |
| Fol-EC19-F | ATCTTCTCACTCTACGGGCCCCATTGAGAGCCGTCAG |
| Fol-EC19-R | TCCTCTCCAAATTACGTTAGTTAGTGCCGCTGCATCCAACAA |
| Fol-EC20-F | ATCTTCTCACTCTACGGCTCCTCAGGAGATCCCCAACAT |
| Fol-EC20-R | TCCTCTCCAAATTACGTTAAGCGTAGGTGTCAATGAAAGAGC |
| Fol-EC21-F | ATCTTCTCACTCTACGATTCCTCCTCGCTGCGCCAT |
| Fol-EC21-R | TCCTCTCCAAATTACGTTACAGGGGAGAGTTGCCAAAAGCAG |
| Fol-EC22-F | ATCTTCTCACTCTACGGACCAAAGGGCTCAACTTCGTG |
| Fol-EC22-R | TCCTCTCCAAATTACGTCAAGGCTTGCATGTATCATAAGGC |
| Tobacco-PR1a-SP-F | CGGCGGAATTCATGGGATTTGTTCTCTTTTCACAATTG |
| Tobacco-PR1a-SP-R | GCCGCCTCGAGTGGATTTTGGGCACGGCAAG |
| Fol-EC14-SP-F | CGGCGGAATTCATGCGTACCGCTGCATTCATCA |
| Fol-EC14-SP-R | CGGCGCTCGAGGGCAGAGACCACGGAGGCCA |
| Fol-EC19-SP-F | CGGCGGAATTCATGCTCTTCTTCAAGTCTATCGCTTCTC |
| Fol-EC19-SP-R | CGGCGCTCGAGGGCGACAGCCAGGCTGAC |
| Fol-EC20-SP-F | CGGCGGAATTCATGGTCAAGTTCGCTTCCGTCGTTG |
| Fol-EC20-SP-R | CGGCGCTCGAGGGCAGCAGCCAGGGGAGC |

**Table S2. Summary of RNA-seq statistics for three replicates of three infection time points (2, 4 and 6 dpi) and mycelium grown *in vitro*.**

| RNA-seq sample | Number of raw reads | Number of clean reads | GC content(%) | Clean reads ratio | Mapped reads | Mapping ratio |
| --- | --- | --- | --- | --- | --- | --- |
| AF2_1 | 84884546 | 83079414 | 43.36 | 97.87% | 555014 | 0.65% |
| AF2_2 | 97408224 | 95210844 | 43.62 | 97.74% | 1318324 | 1.35% |
| AF2_3 | 80258070 | 78588610 | 43.66 | 97.92% | 831734 | 1.04% |
| AF4_1 | 90516752 | 88533756 | 43.22 | 97.81% | 352032 | 0.39% |
| AF4_2 | 84728224 | 83485070 | 43.63 | 98.53% | 593482 | 0.70% |
| AF4_3 | 80166762 | 78429552 | 43.49 | 97.83% | 425126 | 0.53% |
| AF6_1 | 84212910 | 82855476 | 43.43 | 98.39% | 431914 | 0.51% |
| AF6_2 | 89148004 | 87395088 | 43.38 | 98.03% | 491022 | 0.55% |
| AF6_3 | 91606426 | 89749870 | 43.01 | 97.97% | 566268 | 0.62% |
| MY1 | 97931516 | 96012910 | 52.96 | 98.04% | 95214648 | 97.23% |
| MY2 | 105534326 | 102278050 | 52.83 | 96.91% | 101412200 | 96.09% |
| MY3 | 99235754 | 96520570 | 52.92 | 97.26% | 95963480 | 96.70% |

**Table S4. Summary comparison of the Fol4287 genome assemblies and annotations obtained with Sanger and PacBio sequencing platforms.**

| Genome | *Fol* 2010 | *Fol* 2020 |
| --- | --- | --- |
| GeneBank Accession number | GCA_000149955.2 | GCA_001703175.2 |
| Sequencing technology | Sanger | PacBio RSII |
| Number of contigs | 1362 | 47 |
| Genome size (Mb) | 61.47 | 56.21 |
| GC_content(%) | 48 | 48 |
| Contig N50 (kb) | 95.4 | 4140.2 |
| Number of protein-coding genes | 20925 | 26826 |
| Predicted genome assembly completeness (%)^#^ | 99.2 | 99.6 |
| Predicted proteome completeness (%)^#^ | 51.2 | 99.5 |
|  |  |  |

^#^ Completeness = (total BUSCOs − missing BUSCOs)/total BUSCOs × 100

BUSCO version: 5.2.1

Lineage dataset: fungi_odb10

Number of genomes: 549

Number of BUSCOs: 758.

Creation date: 2021-06-28
